# *Aethionema arabicum* dimorphic seed trait resetting during transition to seedlings

**DOI:** 10.3389/fpls.2024.1358312

**Published:** 2024-03-08

**Authors:** Waheed Arshad, Tina Steinbrecher, Per K.I. Wilhelmsson, Noe Fernandez-Pozo, Marta Pérez, Zsuzsanna Mérai, Stefan A. Rensing, Jake O. Chandler, Gerhard Leubner-Metzger

**Affiliations:** ^1^ Seed Biology and Technology Group, Department of Biological Sciences, Royal Holloway University of London, Egham, United Kingdom; ^2^ Plant Cell Biology, Faculty of Biology, University of Marburg, Marburg, Germany; ^3^ Department Plant Breeding and Physiology, Institute for Mediterranean and Subtropical Horticulture “La Mayora” (IHSM-CSIC-UMA), Málaga, Spain; ^4^ Gregor Mendel Institute of Molecular Plant Biology, Austrian Academy of Sciences, Vienna Biocenter (VBC), Vienna, Austria; ^5^ Centre for Biological Signalling Studies (BIOSS), University of Freiburg, Freiburg, Germany; ^6^ Faculty of Chemistry and Pharmacy, University of Freiburg, Freiburg, Germany; ^7^ Laboratory of Growth Regulators, Faculty of Science, Palacký University and Institute of Experimental Botany, Czech Academy of Sciences, Olomouc, Czechia

**Keywords:** fruit and seed heteromorphism, bet-hedging strategy, diaspore dimorphism, seed seedling transition, transcriptome resetting, seedling stress resilience, pre-emergence growth, pericarp-imposed dormancy

## Abstract

The transition from germinating seeds to emerging seedlings is one of the most vulnerable plant life cycle stages. Heteromorphic diaspores (seed and fruit dispersal units) are an adaptive bet-hedging strategy to cope with spatiotemporally variable environments. While the roles and mechanisms of seedling traits have been studied in monomorphic species, which produce one type of diaspore, very little is known about seedlings in heteromorphic species. Using the dimorphic diaspore model *Aethionema arabicum* (Brassicaceae), we identified contrasting mechanisms in the germination responses to different temperatures of the mucilaginous seeds (M^+^ seed morphs), the dispersed indehiscent fruits (IND fruit morphs), and the bare non-mucilaginous M^−^ seeds obtained from IND fruits by pericarp (fruit coat) removal. What follows the completion of germination is the pre-emergence seedling growth phase, which we investigated by comparative growth assays of early seedlings derived from the M^+^ seeds, bare M^−^ seeds, and IND fruits. The dimorphic seedlings derived from M^+^ and M^−^ seeds did not differ in their responses to ambient temperature and water potential. The phenotype of seedlings derived from IND fruits differed in that they had bent hypocotyls and their shoot and root growth was slower, but the biomechanical hypocotyl properties of 15-day-old seedlings did not differ between seedlings derived from germinated M^+^ seeds, M^−^ seeds, or IND fruits. Comparison of the transcriptomes of the natural dimorphic diaspores, M^+^ seeds and IND fruits, identified 2,682 differentially expressed genes (DEGs) during late germination. During the subsequent 3 days of seedling pre-emergence growth, the number of DEGs was reduced 10-fold to 277 root DEGs and 16-fold to 164 shoot DEGs. Among the DEGs in early seedlings were hormonal regulators, in particular for auxin, ethylene, and gibberellins. Furthermore, DEGs were identified for water and ion transporters, nitrate transporter and assimilation enzymes, and cell wall remodeling protein genes encoding enzymes targeting xyloglucan and pectin. We conclude that the transcriptomes of seedlings derived from the dimorphic diaspores, M^+^ seeds and IND fruits, undergo transcriptional resetting during the post-germination pre-emergence growth transition phase from germinated diaspores to growing seedlings.

## 1 Introduction

The transition from germinating seeds to emerging seedlings is one of the most vulnerable plant life cycle stages, which depends on seed and seedling phenotypic plasticity and complex interactions with environmental cues (Fenner, 1987; Walck et al., 2011; Gardarin et al., 2016). Seed germination and fruit germination depend on basic requirements for water, oxygen, and an appropriate temperature and are generally considered to be completed by radicle protrusion (visible germination). Germination is further spread over time by dormancy mechanisms, which block germination under favorable conditions so that germination occurs when conditions for establishing a new plant generation are likely to be suitable (Finch-Savage and Leubner-Metzger, 2006; Baskin and Baskin, 2014; Finch-Savage and Footitt, 2017). Successful seedling establishment and spreading seedling emergence over time, therefore, depend primarily on germination timing; in addition, post-germination seedling traits are of major importance. What follows the completion of germination is the pre-emergence seedling growth phase, which may be prone to increasing post-germination stress in the soil environment, and consequently, seeds/seedlings are often lost and fail to establish during this stage (Moles and Westoby, 2006; Finch-Savage and Bassel, 2016; Gardarin et al., 2016). Pre-emergence seedling growth is heterotrophic growth in which the seeds’ storage reserves are used to establish a root and to fuel shoot elongation until the seedling emerges from the soil and switches to greening and self-nourishing autotrophic growth (Fenner, 1987; Finch-Savage and Bassel, 2016; Ha et al., 2017; Smolikova et al., 2022). While the roles and mechanisms of seedling traits have been studied in monomorphic species, which produce one type of diaspore (dispersed seeds or fruits), very little is known about seedlings in heteromorphic species.

Diaspore (seed/fruit) heteromorphism is the production by an individual plant of two (dimorphism) or more distinct kinds of seeds and/or fruits that differ in morphological (e.g., mass, shape, and color), dispersal ability (e.g., dormancy and mode of dispersal), and other diaspore properties (Imbert, 2002; Baskin and Baskin, 2014; Baskin et al., 2014). Heteromorphic diaspore traits have been proposed to be an adaptive bet-hedging strategy to cope with spatiotemporally variable environments. Distinct dormancy breaking requirements of the morphs cause differences in germination timing, and consequently, seedling emergence is spread over time and space (Maun and Payne, 1989; Lu et al., 2017a; b; Arshad et al., 2019; Lu et al., 2020). For example, comparison of seedlings derived from the dimorphic seeds of the cold desert halophyte *Suaeda corniculata* demonstrated that seedlings from black seeds emerged in July and August, and those from brown seeds emerged in May, and these dimorphic seedlings also differed in size and root/shoot ratio (Cao et al., 2012; Yang et al., 2015). Seedlings derived from dimorphic seeds of *Atriplex centralasiatica* and other *Suaeda* species differed in salinity tolerance (Xu et al., 2011; Zhang et al., 2021; Cao et al., 2022; Song et al., 2023). Whether seedlings derived from dimorphic seeds also differ in responsiveness to other abiotic stress factors such as heat or drought has not been investigated.

**Figure 1:**
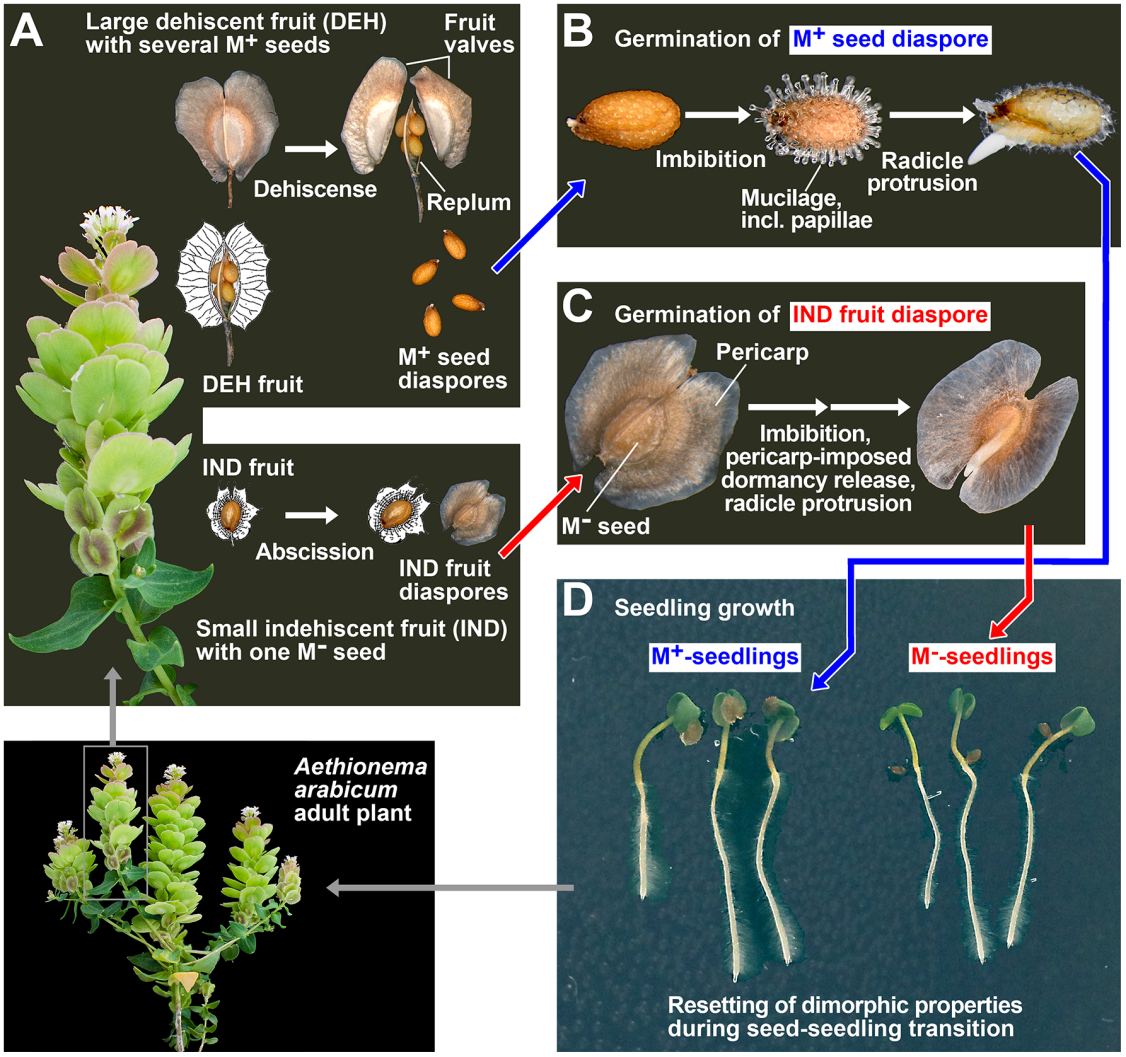
Annual life cycle of the dimorphic diaspore model *Aethionema arabicum*. **(A)** Dimorphic fruit and seed development and dispersal of the M^+^ seed and IND fruit diaspores. **(B)** Germination of the M^+^ seed diaspore. **(C)** Germination of the IND fruit diaspore. **(D)** Seedling growth of M^+^ and M^−^ seedlings derived from the M^+^ and M^−^ seeds, respectively. Resetting of the dimorphism during pre-emergence growth leads to adult plants that are indistinguishable regarding their M^+^ or IND origin. These plants restart producing dimorphic diaspores during reproduction. Rearranged and redrawn using parts from Arshad et al. (2019).

Here, we exploit the diaspore dimorphism of *Aethionema arabicum* (**Figure 1**), an annual member of the earliest diverging sister tribe within the Brassicaceae, in which seed and fruit dimorphism was associated with a switch to an annual life history (Lenser et al., 2016; Mohammadin et al., 2017; Chandler et al., 2024). *Ae. arabicum* is adapted to arid and semiarid environments, its life-history strategy appears to be a blend of bet-hedging and phenotypic plasticity (Bhattacharya et al., 2019b), and it exhibits true seed and fruit dimorphism with no intermediate morphs (Lenser et al., 2016). Two distinct fruit types are produced on the same infructescence: dehiscent (DEH) fruits, with four to six mucilaginous (M^+^) seeds, and indehiscent (IND) fruits, each containing a single non-mucilaginous (M^−^) seed. Upon maturity, DEH fruits shatter, releasing the M^+^ seeds, while the dry IND fruits are dispersed in their entirety by abscission. Two very contrasting biophysical and ecophysiological dispersal mechanisms of the *Ae. arabicum* dimorphic diaspores were revealed (Arshad et al., 2019). Dehiscence of large fruits leads to the release of M^+^ seed diaspores, which adhere to substrata via seed coat mucilage, thereby preventing dispersal (anti-telechory). IND fruit diaspores (containing non-mucilaginous seeds) disperse by wind or water currents, promoting dispersal (telechory) over a longer range. The pericarp properties confer enhanced dispersal ability and degree of dormancy to the IND fruit morph to support telechory, while the M^+^ seed morph supports anti-telechory. The germination of M^+^ seeds of some *Ae. arabicum* accessions is inhibited by light, while other *Ae. arabicum* accessions, including the widely used TUR (Turkey) accession, germinate equally well in continuous light and darkness (Merai et al., 2019). Dimorphic fruits and seeds of the *Ae. arabicum* TUR accession differ in their molecular mechanisms throughout their development on the mother plant, in the mature dry state upon dispersal, and in dormancy and germination properties during imbibition (Lenser et al., 2018; Arshad et al., 2019; Wilhelmsson et al., 2019; Nichols et al., 2020; Arshad et al., 2021; Steinbrecher and Leubner-Metzger, 2022; Chandler et al., 2024). We demonstrate here that the seedlings of the *Ae. arabicum* TUR accession derived from the dimorphic diaspores (M^+^ seeds and IND fruits) differ during their pre-emergence growth and undergo resetting of their transcriptomes during the transition from germinated diaspores to early seedlings.

## 2 Results

### 2.1 Resetting of distinct *Ae. arabicum* dimorphic diaspore responses to abiotic stresses during the seed-to-seedling transition phase

**Figure 1** shows the life cycle of the annual *Ae. arabicum*, which is characterized by the production and dispersal of dimorphic diaspores. The M^+^ seed diaspore is dispersed by dehiscence (pod shatter), and upon imbibition, it produces a mucilaginous layer during germination (**Figure 1B**). The IND fruit diaspore is dispersed by abscission and constitutes an indehiscent fruit in which the single M^−^ seed is covered by the pericarp (fruit coat), which confers coat dormancy and prevents or delays germination (**Figure 1C**). Earlier work with *Ae. arabicum* TUR (Chandler et al., 2024) revealed the molecular and morphological mechanisms underpinning the distinct dormancy and germination responses of the dimorphic diaspores to different imbibition temperatures. A comparison of the maximal germination percentages (G_max_) and the germination rates (speed) of germination (GR_50_; i.e., the inverse of the time of the diaspore population to reach 50% radicle protrusion) identified 14°C as the optimal temperature for the highest germination speed of the M^+^ seed morphs, the bare M^−^ seeds (manually extracted from IND fruits by pericarp removal), and the IND fruit morphs (**Supplementary Figure 1**). The IND fruit morph, however, exhibited a degree of pericarp-imposed dormancy across the entire temperature window. Thus, our initial physiological experiments investigating the effects of abiotic stress factors on seedling growth focused on comparing M^+^ and M^−^ seedlings derived from germinated M^+^ and bare M^−^ seeds, respectively. These experiments, involving the removal of the pericarp to obtain exposed M^−^ seeds, align with dimorphic systems (see the Introduction) characterized by true seeds of distinct colors as diaspores. In such systems, the resulting seedlings exhibit differential responses to salinity, with the unique seedling reactions having already been established during dimorphic seed development (Xu et al., 2011; Zhang et al., 2021; Cao et al., 2022; Song et al., 2023). In our subsequent comparative biomechanical and transcriptome analyses with *Ae. arabicum* TUR, we compared the seed–seedling transition phase and early growth of seedlings derived from germinated M^+^ seeds, bare M^−^ seeds, and IND fruits. Adult plants grown from M^+^ and M^−^ seedlings are indistinguishable from each other and produce dimorphic diaspores (**Figure 1**).

**Figure 2:**
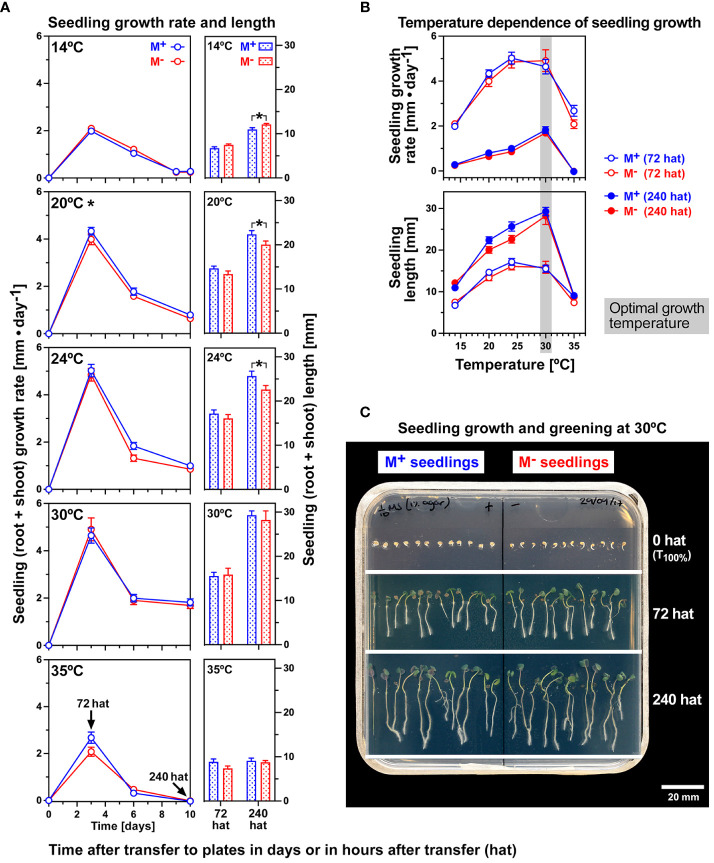
The effect of a range of constant temperatures on the growth of *Aethionema arabicum* M^+^ and M^−^ seedlings. **(A)** M^+^ and M^−^ seedlings were grown on vertical agar plates at constant temperatures as indicated. Seedling growth was scored over time starting at 0 hat (hours after transfer). The mean ± SEM (N = 3 plates, each with seven seedlings) of seedling growth rates over time and seedling lengths at 72 and 240 hat are presented; for further details, see **Supplementary Figure 2**. ANOVA of growth rates revealed that morph had no effect overall at 14°C (p = 0.114), 24°C (p = 0.089), 30°C (p = 0.959), or 35°C (p = 0.217), while at 20°C (p = 0.027), M^+^ seedlings grew at a faster rate than M^−^ seedlings. Statistical analysis (unpaired t-test) of day 10 M^+^ and M^−^ seedlings demonstrated that the slightly different lengths between M^+^ and M^−^ seedlings were significant (*) at 14°C (M^−^ seedlings slightly longer, p = 0.012), 20°C (M^−^ seedlings slightly shorter, p = 0.049), and 24°C (M^−^ seedlings slightly shorter, p = 0.040), while no significant length difference was obtained at 30°C and 35°C. **(B)** Temperature dependence of seedling growth rate and seedling length at 72 and 240 hat. The optimal seedling growth temperature (30°C) is indicated. ANOVA of 72 and 240-hat seedling growth rates and seedling length across the entire temperature range revealed no significant differences between the morphs (M^+^ versus M^−^). **(C)** M^+^ and M^−^ seedlings were grown on vertical agar plates in continuous white light (170 µmol·m^−2^·s^−1^). Seedling growth assays were conducted with seedlings derived from germinated M^+^ and M^−^ seeds, which were selected for transfer to agar plates containing media based on 1-mm protrusion of the radicle (0 hat).

To study the effects of thermal stress on M^+^ and M^−^ seedling growth independently from the temperature effects on dimorphic diaspore germination (**Supplementary Figure 1**), M^+^ and M^−^ seeds were imbibed at an optimal germination temperature, and seeds that had just completed germination (1-mm radicle protrusion visible) were transferred to agar plates for conducting the seedling growth assay (**Figure 2**). M^+^ and M^−^ seedlings grown on vertical agar plates at 14°C, 20°C, 24°C, 30°C, and 35°C were compared for their root and shoot lengths at the times indicated [in “hat” (hours after transfer)], and seedling growth rates were calculated. Though the total seedling length differed significantly in several cases between the morphs, the difference was very small, and there was no overall morph-specific physiological response. Similarly, seedling growth rates did not differ between the M^+^ and M^−^ morphs (**Figure 2A**). The fastest seedling growth rate was observed at 72 hat, and 30°C was identified as the temperature for fastest growth leading to the longest seedlings at 240 hat (**Figure 2B**; **Supplementary Figure 2**). Fresh and dry weights of separated root and shoot tissue at the end of the experiment (10 days) revealed a strong temperature response (p < 0.001), with the highest masses observed at 30°C (**Supplementary Figure 2**). Together with seedling lengths, growth rates, and fresh weights, the most optimal seedling growth condition considered was thus 30°C, likely corresponding with the maximum climatic temperatures of sites in Turkey from which *Ae. arabicum* TUR was collected (Arshad et al., 2019). At 35°C, there were slight indications that M^+^ seedlings had greater vigor, with a higher mean shoot fresh and dry weight than M^−^ seedlings (**Supplementary Figure 2**). Temperature, therefore, had a profound effect on M^+^ and M^−^ seedling growth, in particular on post-germination pre-emergence growth until 72 hat (**Figure 2**).

To study the effect of osmotic stress on the growth of *Ae. arabicum* M^+^ and M^−^ seedlings, their growth was analyzed at lowered water potentials using high-molecular-weight polyethylene glycol (PEG). After more than 3 weeks of vertical growth, seedling morphs did not differ in their total length and growth rates under three different concentrations of PEG (**Supplementary Figure 3**). Taken together, no differences in the physiological responses of M^+^ and M^−^ seedlings derived from M^+^ seeds and bare M^−^ seeds to temperature and reduced water potential were identified. This suggests that during the seed–seedling transition, the observed dimorphic diaspore trait differences observed in abiotic stress responses (Lenser et al., 2016, 2018; Arshad et al., 2019; Wilhelmsson et al., 2019; Bhattacharya et al., 2019a, b; Arshad et al., 2020, 2021; Fernandez-Pozo et al., 2021; Chandler et al., 2024) may be reset to a large extent. To test this hypothesis, we conducted transcriptome analysis.

### 2.2 Comparative RNA-seq analysis of late germination and post-germination pre-emergence seedling growth reveals gradual resetting of transcriptomes

**Figure 3:**
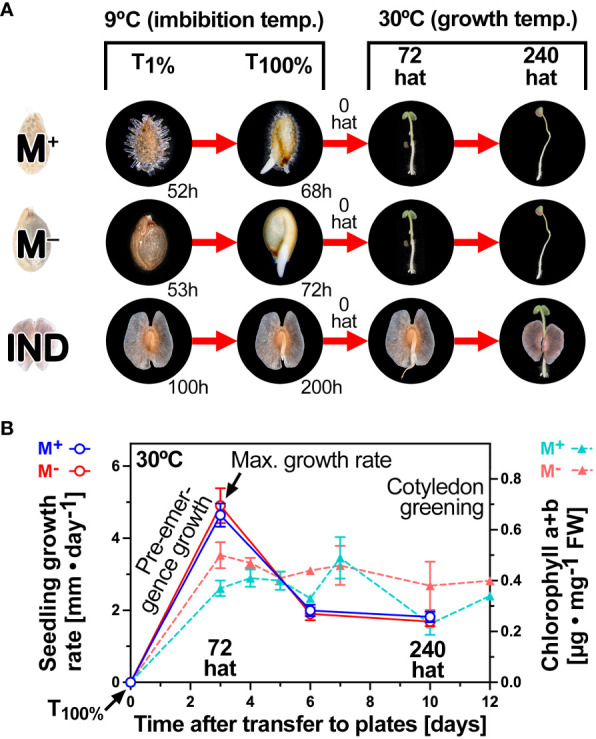
Diaspore germination and seedling growth phases for the comparative transcriptome analysis. **(A)** Overall experimental design for comparative RNA-seq analysis of diaspore germination and seedling establishment. Time points were selected during completion of germination (T_1%_ and T_100%_; times of these are indicated), early [72 hat (hours after transfer)], and late (240 hat) seedling growth. Within-root and within-shoot tissue pairwise comparisons were based on the effect of the seed morph (M^+^ seed vs. bare M^−^ seed), the effect of the pericarp (M^−^ seed vs. IND fruit), and the ecological dispersal unit (M^+^ seed vs. IND fruit). **(B)** Key events and phases of seedling growth at the optimal temperature (30°C) and time course analysis of chlorophyll accumulation during cotyledon greening. Error bars = ± 1 SEM. N = 3, each with 10 replicate seedlings.

**Figure 3A** depicts the experimental design for the RNA-seq analysis in which we compared the transcriptomes during the late germination phase with those during early seedling growth. Samples were collected from imbibed *Ae. arabicum* M^+^ seeds, M^−^ seeds, and IND fruits at T_1%_, i.e., the time for the onset of the completion of germination of a seed population, and at T_100%_, i.e., the time when the entire population had completed germination by visible radicle protrusion (**Figure 3A**). It is known from previous work (Wilhelmsson et al., 2019; Arshad et al., 2021; Chandler et al., 2024) that the pericarp of imbibed IND fruits is dead tissue that does not contain any RNA and that dry seed and germination transcriptomes until T_1%_ differ considerably between the dimorphic diaspores. The physiological sampling times for T_1%_ and T_100%_ of M^+^ seeds, bare M^−^ seeds, and IND fruits are indicated in **Figure 3A**. Diaspores that had just completed germination (1-mm radicle protrusion) were transferred to agar plates at T_100%_ (0 hat) for conducting the seedling growth assay. Root and shoot tissues were harvested from seedling samples at key physiological stages (72 hat and 240 hat) during early seedling growth (**Figure 3**).

**Figure 4:**
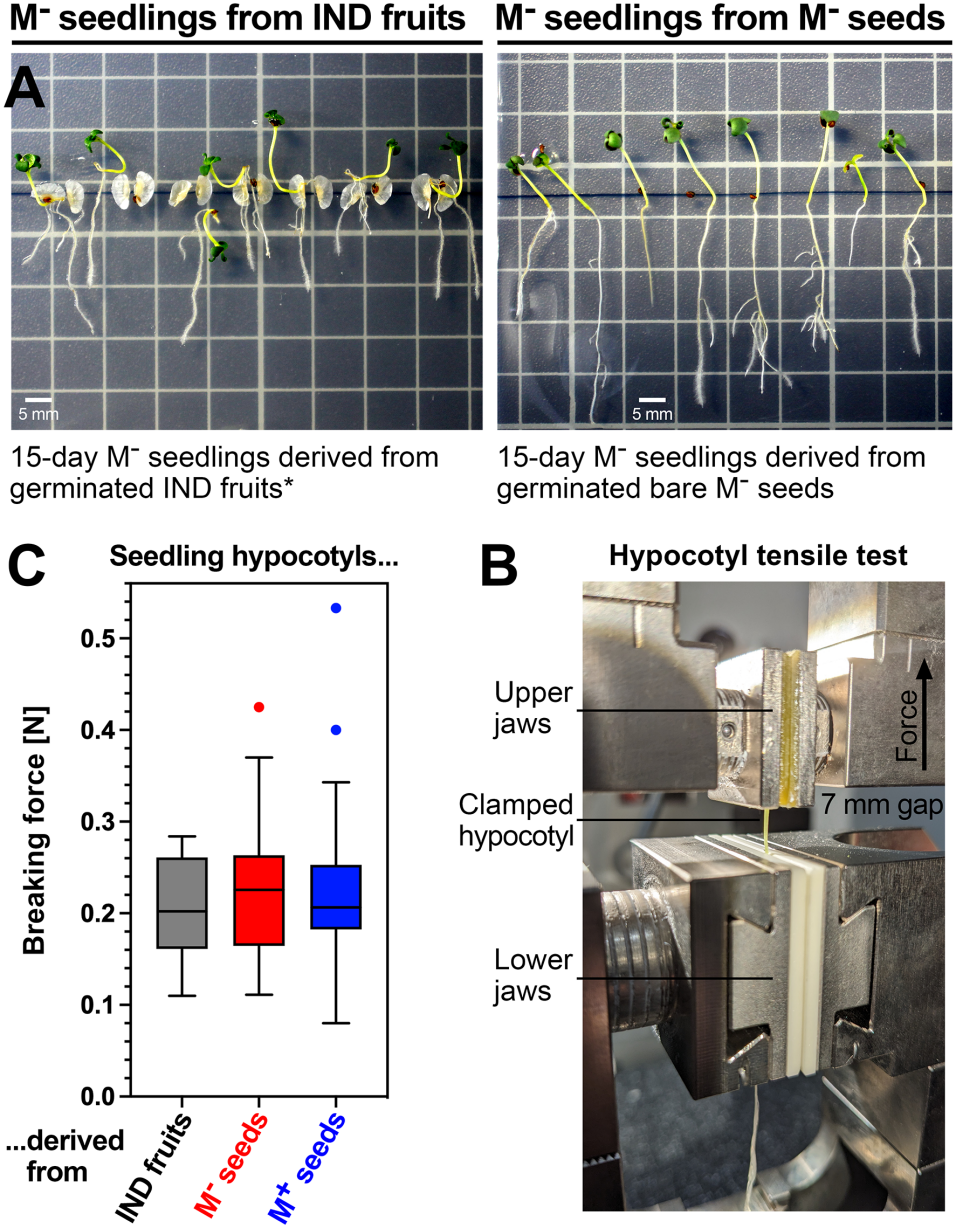
Comparative biomechanical analysis of *Aethionema arabicum* seedlings derived from M^+^ seeds, M^−^ seeds, and IND fruits. **(A)** Fifteen-day-old seedlings from germinated (0 hat) IND fruits (*left panel*) and bare M^−^ seeds (*right panel*). * IND pericarps of germinated fruits (at 0 hat) were manually split open to aid seedling growth. Note that seedlings derived from IND fruits often had bent lower hypocotyls and, in general, grew slower compared to seedlings derived from M^−^ seeds, which had straight hypocotyls. **(B)** Hypocotyl tensile test. **(C)** Hypocotyl breaking forces of 15-day-old seedlings derived from IND fruits, bare M^−^ seeds, and M^+^ seeds. Box plots with Tukey’s whiskers of hypocotyl breaking forces are presented from force-displacement data obtained using N = 42 (M^+^), N = 38 (M^−^), and N = 15 (IND) seedlings. The hypocotyls show no significant difference in their breaking force.

The 72-hat pre-emergence growth samples correspond to the maximal growth rate, and 72 hat was associated with the onset of cotyledon greening by chlorophyll accumulation (**Figure 3B**). Between 6 and 10 days, the growth rates of M^+^ and M^−^ seedlings derived from germinated M^+^ seeds and bare M^−^ seeds, respectively, remained roughly equal. M^+^ and M^−^ seedlings derived from germinated M^+^ seeds and bare M^−^ seeds had straight hypocotyls. In contrast to this, seedlings derived from IND fruits often exhibited a bent lower hypocotyl connected with an overall slower shoot and root growth (**Figure 4A**). To investigate if morph caused a long-lasting effect on the biomechanical properties of the hypocotyls, we conducted comparative hypocotyl tensile tests of 15-day-old seedlings (**Figure 4B**). No significant differences in the hypocotyl breaking force were evident between the seedlings derived from germinated M^+^ seeds, bare M^−^ seeds, and IND fruits (**Figure 4C**). The observed difference in hypocotyl shape (bent versus straight) and the slower shoot and root growth of IND fruit-derived seedlings, therefore, did not affect the hypocotyl biomechanical properties at this stage of seedling growth.

**Figure 5:**
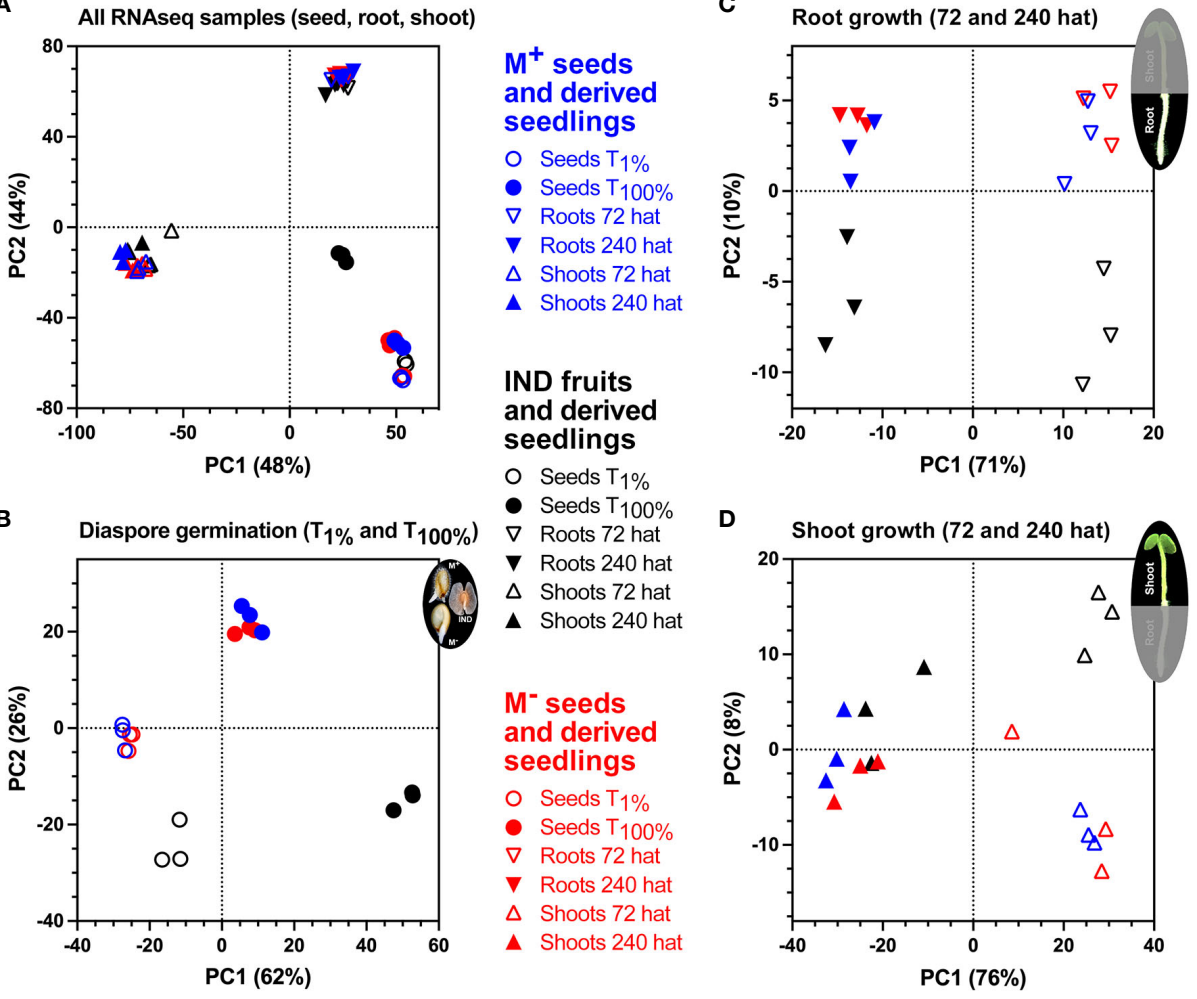
Principal component analysis (PCA) of *Aethionema arabicum* seed and seedling RNA-seq samples. **(A)** PCA of RNA-seq samples obtained during M^+^ seed, M^−^ seed, and IND fruit germination and seedling establishment. Colors indicate morph, while symbols indicate seed, root, or shoot tissue at 1% germination (T_1%_), 100% germination (T_100%_), or 72 or 240 hours after transfer (hat) to seedling growth plates. **(B)** PCA of RNA-seq samples during diaspore germination. **(C)** PCA of RNA-seq samples during seedling root growth at 72 and 240 hat. **(D)** PCA of RNA-seq samples during seedling shoot growth at 72 and 240 hat.

To provide insights into the association between RNA-seq samples, datasets were visualized using principal component analysis (PCA), based on the 500 genes with the highest variance. Replicate RNA-seq samples clustered tightly by diaspore and by organ (root and shoot) type (**Figure 5A**). As expected, three distinct clusters were separated primarily by the derivation of the samples from seed, root, or shoot tissue based on the first two components, explaining 48% and 44% of the variability. In this combined analysis (**Figure 5A**), IND samples at T_100%_ (germinated diaspores) remain distinct from all other tissues and time points. Separation of samples involved in key processes of germination (T_1%_ and T_100%_), root growth (72 and 240 hat), and shoot growth (72 and 240 hat) revealed comparative transcriptome profiles between the morphs in greater detail (**Figure 5**). Clear differences were observed with IND samples, such that both T_1%_ and T_100%_ samples clustered separately from M^+^ and M^−^ seed samples. M^+^ and M^−^ seed samples, however, showed tight correlations throughout the course of germination (**Figure 5B**). As seedlings, differences between morphs appear to be smaller. M^+^ and M^−^ samples clustered together during the two time points during root (**Figure 5C**) and shoot (**Figure 5D**) growth. IND root samples remain distinct from M^+^ and M^−^ samples. However, transcriptional profiles of IND shoot tissue suggest that while samples at 72 hat remain as a separate cluster, by 240 hat, there is a tendency toward greater similarity of M^+^ and M^−^ samples.

**Figure 6:**
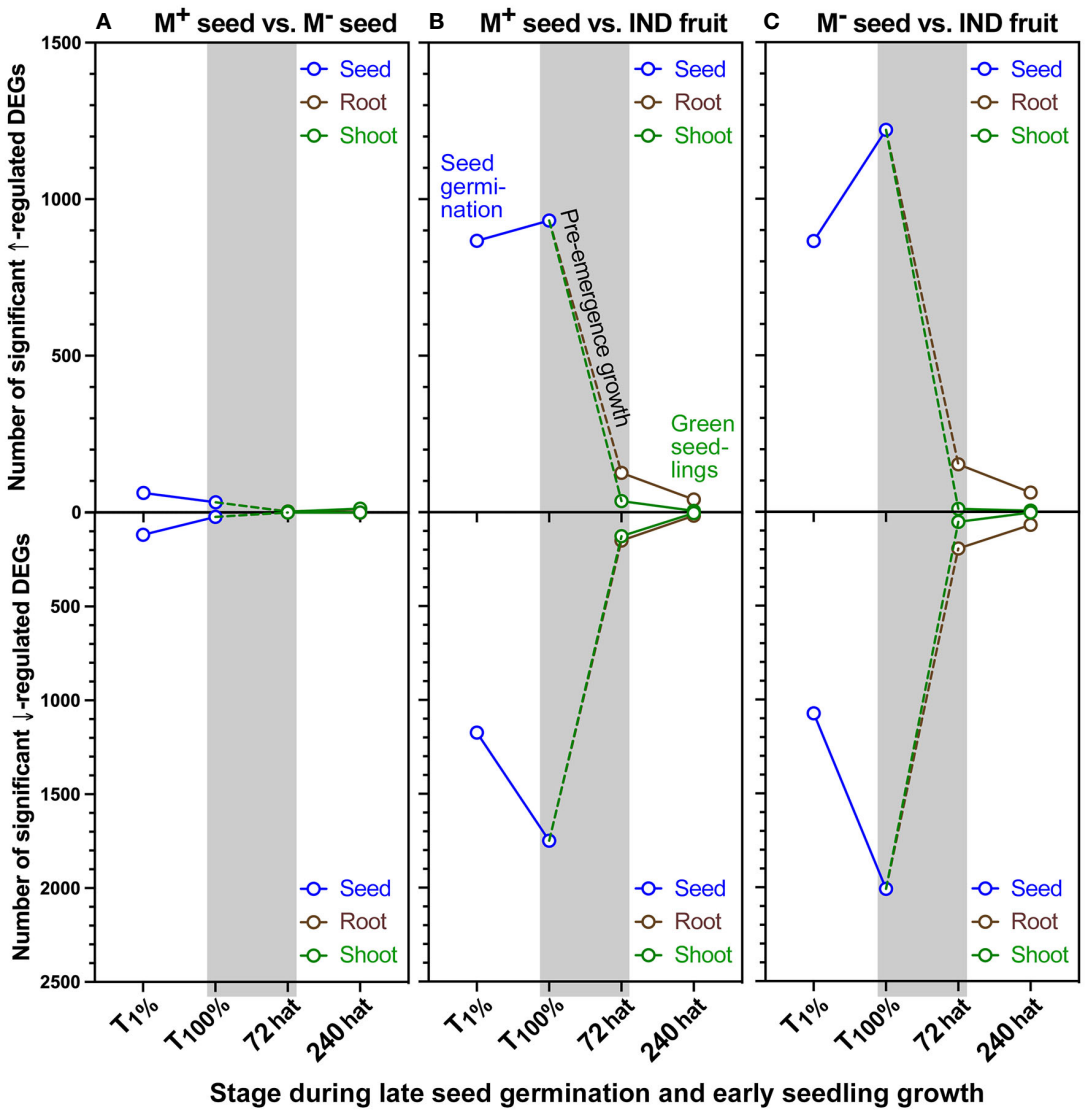
Number of differentially expressed genes (DEGs) identified during the developmental transition from germination to seedling establishment of *Aethionema arabicum*. Total number of DEGs detected during the developmental transition from germination to seedling establishment. Shown are DEGs upregulated (↑) and downregulated (↓) based on pairwise comparisons of **(A)** M^+^ seed vs. M^−^ seed (M^+^/M^−^), **(B)** M^+^ seed vs. IND fruit (M^+^/IND), and **(C)** M^−^ seed vs. IND fruit (M^+^/IND). In all comparisons, the second treatment type was the baseline to which the first treatment was compared as transcript abundance ratio, i.e., M^+^/M^−^, M^+^/IND, and M^−^/IND. The dashed line indicates a hypothetical trajectory of DEGs for the corresponding tissues. DEG detection was performed using the pipeline-adjusted p-value cutoff set to 0.001 as previously described by Wilhelmsson et al. (2019). Symbols indicate seed, root, or shoot tissue at 1% germination (T_1%_), 100% germination (T_100%_), or 72 or 240 hours after transfer (hat) to seedling growth plates. For further details, see **Supplementary Table 1** and **Supplementary Dataset 1**.

Cleaned RNA-seq reads mapped to the *Ae. arabicum* genome were obtained for 23,594 genes (**Supplementary Dataset 1**). To make the transcript abundance data easily and publicly accessible, a gene expression atlas was generated, which was implemented in the *Ae. arabicum* genome database (DB) (Fernandez-Pozo et al., 2021) at https://plantcode.cup.uni-freiburg.de/aetar_db/index.php. The *Ae. arabicum* gene expression atlas includes the transcriptome results of this work and work published earlier (Merai et al., 2019; Wilhelmsson et al., 2019; Arshad et al., 2021; Chandler et al., 2024) and allows adding future transcriptome datasets. The transcript abundance data for the 23,594 *Ae. arabicum* genes were further investigated, and differentially expressed genes (DEGs; **Supplementary Dataset 1**) were detected in a strict consensus (overlap) approach using an adjusted p-value cutoff set to 0.001 (Wilhelmsson et al., 2019). Pairwise comparisons of M^+^ seeds vs. bare M^−^ seeds (seeds only), M^+^ seeds vs. IND fruits (natural dispersal units), and M^−^ vs. IND (pericarp effect) allowed transcriptome exploration of the dimorphic syndrome during seed germination and seedling growth. Comparisons showed that M^+^ and M^−^ seed transcriptomes became remarkably similar during the completion of germination (**Figure 6A**; **Supplementary Table 1**). A total of 180 and 55 DEGs were detected at T_1%_ and T_100%_, respectively, while after 72 hat, the seedling transcriptomes were almost identical in the root (three DEGs) and shoot (two DEGs). By contrast, comparisons between the natural diaspores, M^+^ seed vs. IND fruit, showed a much higher number of DEGs during germination. A total of 2,041 DEGs during T_1%_ increased to 2,682 by T_100%_, thereafter reducing 10-fold (277 root DEGs) and 16-fold (164 shoot DEGs) by 72 hat. By 240 hat, the time of true leaf emergence for seedlings, differences between M^+^ seed vs. IND fruit-derived seedlings were only evident from 60 DEGs in root samples and 10 DEGs in shoot samples (**Figure 6B**; **Supplementary Table 1**).

Interestingly, a similar but more pronounced pattern of DEGs was observed in the comparison of the pericarp effect (bare M^−^ seed vs. IND fruit) on M^−^ seed germination and seedling growth (**Figure 6C**; **Supplementary Table 1**). The number of DEGs at T_1%_ was similar to that detected in the M^+^ vs. IND comparison (2,041 vs. 1,938). The greatest differences were observed at T_100%_ (3,228 DEGs) at the onset of pre-emergence growth. Here, the number of upregulated DEGs in M^−^ seeds increased 1.4-fold (to 1,220 DEGs), while the number of downregulated DEGs increased 1.9-fold (to 2,008 DEGs). As seedlings progressed through root and shoot growth, the pericarp imposed a total of 347 root-specific DEGs at 72 hat, while shoot-specific DEGs were lower (64). By 240 hat, differences in shoot samples comprised four up- and four downregulated DEGs, while 62 up- and 69 downregulated DEGs were detected in the root tissues (**Figure 6C**; **Supplementary Table 1**). Taken together, these results suggest the tendency toward transcriptional “resetting” of seedling morphs mainly during the post-germination pre-emergence growth phase.

**Figure 7:**
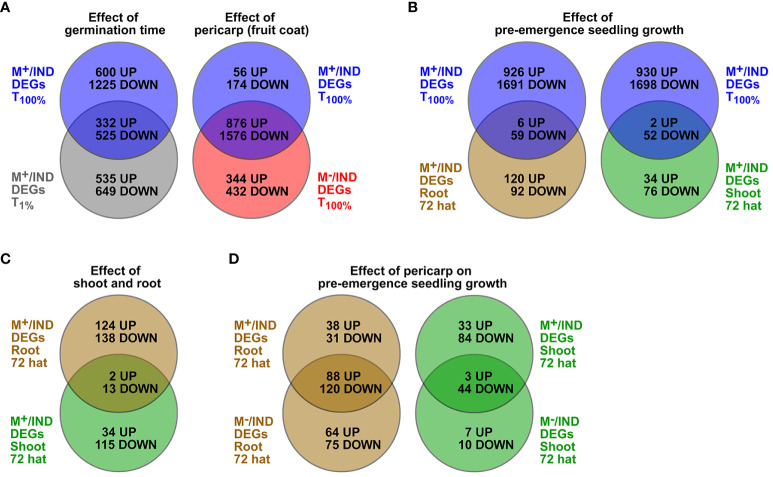
Comparative analysis of *Aethionema arabicum* differentially expressed gene (DEG) lists presented as Venn diagrams. **(A)** Effects of germination time and pericarp as comparisons of the list of M^+^/IND DEGs at T_100%_ (*blue circle*) to either M^+^/IND at T_1%_ (*grey circle*) or M^−^/IND at T_100%_ (*red circle*). **(B)** Effect of pre-emergence growth by comparisons of the list of M^+^/IND DEGs at T_100%_ (blue circle) M^+^/IND 72-hat DEG lists of roots (*brown circle*) or shoots (*green circle*). **(C)** Effect of shoot and root. **(D)** Effect of pericarp on pre-emergence seedling growth by comparing M^+^/IND 72-hat DEG lists with M^−^/IND 72-hat DEG lists for roots (*brown circle*) and shoots (*green circle*). Seed, root, or shoot tissue at 1% germination (T_1%_), 100% germination (T_100%_), or 72 or 240 hours after transfer (hat) to seedling growth plates was compared. For gene lists of overlapping and unique DEGs, see **Supplementary Dataset 1**.

In the ecologically relevant comparison (M^+^ seed vs. IND fruit), shoot resetting occurred earlier (by 72 hat), and root resetting occurred later (differences still evident at 240 hat). Comparison of M^+^/IND DEG lists (**Supplementary Dataset 1**) between the T_100%_ (germinated diaspores) and the T_1%_ (ungerminated diaspores) time points revealed that about one-third of the DEGs are overlapping and two-thirds are unique to either T_100%_ or T_1%_ (**Figure 7A**). Comparison of the M^+^/IND and M^−^/IND DEG lists at T_100%_ revealed that while the majority of the DEGs are overlapping, there is also a considerable number of DEGs unique to either M^+^/IND or M^−^/IND (**Figure 7A**). For the ecologically relevant comparison (M^+^/IND), the comparison between the T_100%_ seed and the 72-hat seedling state delivered root and shoot DEGs common and unique for pre-emergence seedling growth (**Figure 7B**). Comparison of M^+^/IND DEG lists during pre-emergence seedling growth revealed that most of the DEGs at 72 hat are unique for root and shoot, a finding that strongly suggests that the two compartments are distinct (**Figure 7C**). To investigate the effect of the pericarp on the pre-emergence seedling growth at M^+^/IND and M^−^/IND, DEG lists for root and shoot at 72 hat were compared and delivered DEG lists representing pericarp-dependent and pericarp-independent mechanisms (**Figure 7B**; **Supplementary Dataset 1**). These comparative DEG lists (M^+^/IND vs. M^−^/IND) contained for roots a total of 190 up- and 226 downregulated genes and for shoots a total of 43 up- and 138 downregulated genes.

**Figure 8:**
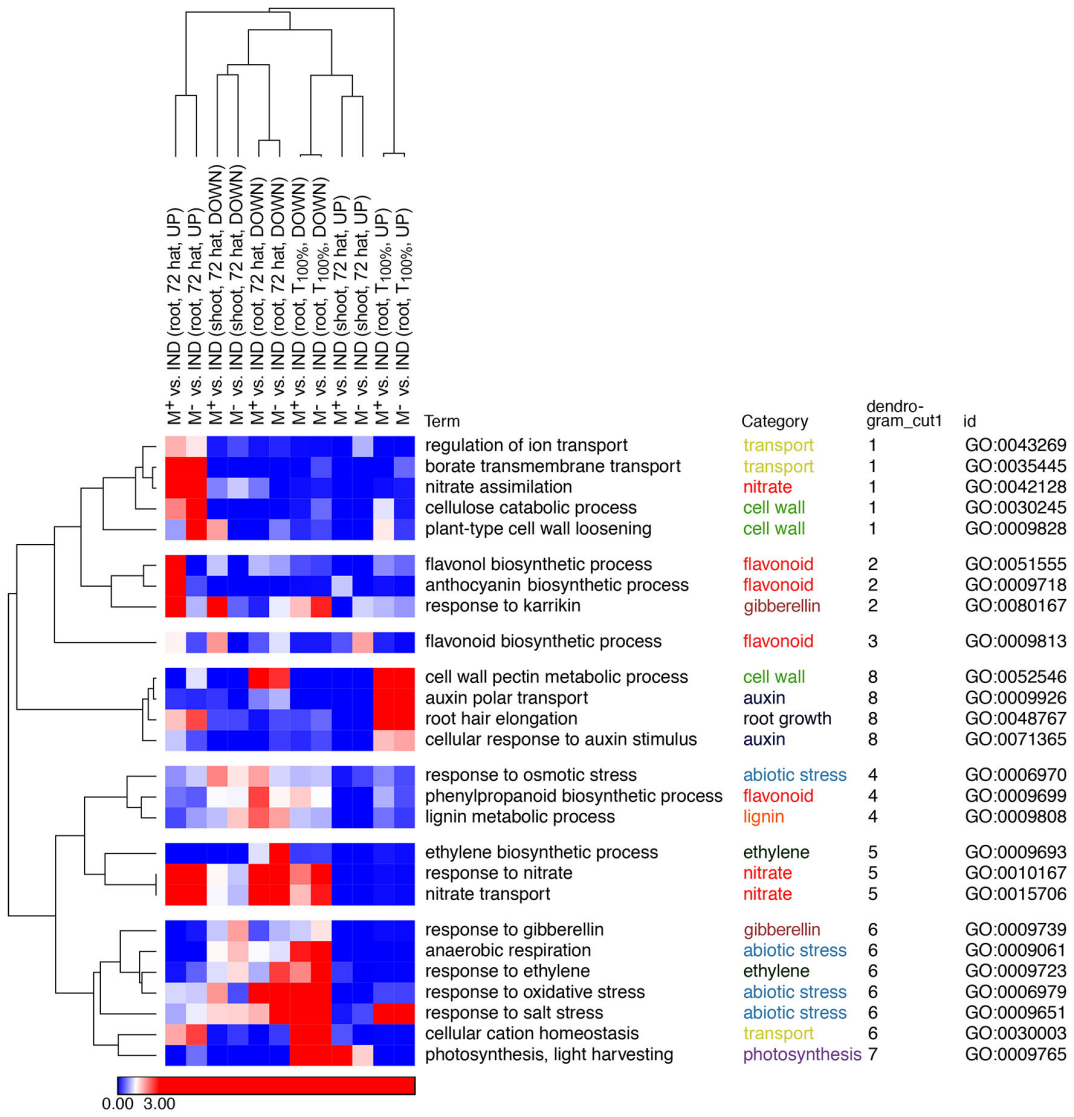
Gene Ontology (GO) term enrichment in differentially expressed gene (DEG) lists. GO terms were selected and assigned categories, and enrichment scores [log(1/p-value)] were clustered hierarchically by 1-Pearson correlation using Morpheus (https://software.broadinstitute.org/morpheus/). GO terms representative of each cluster are shown here, with all selected GO terms shown in **Supplementary Figure 4**, and full GO term enrichment p-values for all DEG lists can be found in **Supplementary Dataset 2**. Blue indicates non-significantly enriched values (p > 0.05), with white representing the significant cutoff (p = 0.05) and red indicating where GO terms are significantly enriched in the DEG lists (p < 0.05), saturated at p = 0.001. Hierarchical clustering was redone with selected GO terms, and the original cluster was based on dendrogram cut **Supplementary Figure 4** as indicated.

To gain insight into the processes differing during the transition from germinated diaspores to early seedlings derived from M^+^, M^−^, and IND, Gene Ontology (GO) term enrichment analysis of DEG lists was performed comparing M^+^ seeds versus IND fruits and M^−^ seeds versus IND for the T_100%_ (germinated diaspores) and the 72 hat (root and shoot separately) stages (**Figure 8**; **Supplementary Figure 4**, **Supplementary Dataset 2**). Broadly, selected identified GO terms were categorized by key processes identified as differing between seedlings originating from the different diaspores (**Figure 8**; **Supplementary Figure 4**). For example, genes up in roots of seedlings 72 hat derived from M^+^ or M^−^ seeds compared to IND diaspores were significantly enriched in GO terms related to nitrates (e.g., nitrate assimilation), cell wall (e.g., cellulose catabolic process), and transport (e.g., regulation of ion transport) (clusters 1 and 5). Interestingly, some cell wall and nitrate-related terms (e.g., cell wall pectin metabolic process and nitrate transport) were enriched in genes more highly expressed in 72-hat seedlings derived from IND compared to M^+^ and M^−^ (clusters 8 and 5). A pronounced effect of pericarp at the T_100%_ stage was evident in the enrichment of abiotic stress-related GO terms “response to oxidative stress”, “response to salt stress”, and “anaerobic respiration”. Terms identifying hormone signaling (e.g., ethylene, auxin, gibberellin, and ABA) were also identified (**Figure 8**; **Supplementary Figure 4**; **Supplementary Dataset 2**). Overall, it was evident that pericarp presence at the diaspore stage was a driver of differences in gene expression, with similar differences evident when comparing M^+^ or M^−^ seeds against IND diaspores. However, some contrasts were more evident when comparing 72-hat seedlings derived from M^+^ to IND (rather than M^−^ to IND), suggesting seed morph-specific responses that were not dependent on pericarp presence at imbibition, particularly evident in the enrichment of GO terms “flavonol biosynthetic process” and “anthocyanin-containing compound biosynthetic process”. The pre-emergence seedling growth DEGs from these lists of enriched GO categories (**Figure 8**; **Supplementary Figure 4**) and a comparison to early seed germination and dormancy (Chandler et al., 2024) are the focus of the following analysis into the resetting of dimorphic expression patterns.

### 2.3 Resetting of dimorphic expression patterns of hypoxia and hormone-related genes

**Figure 9:**
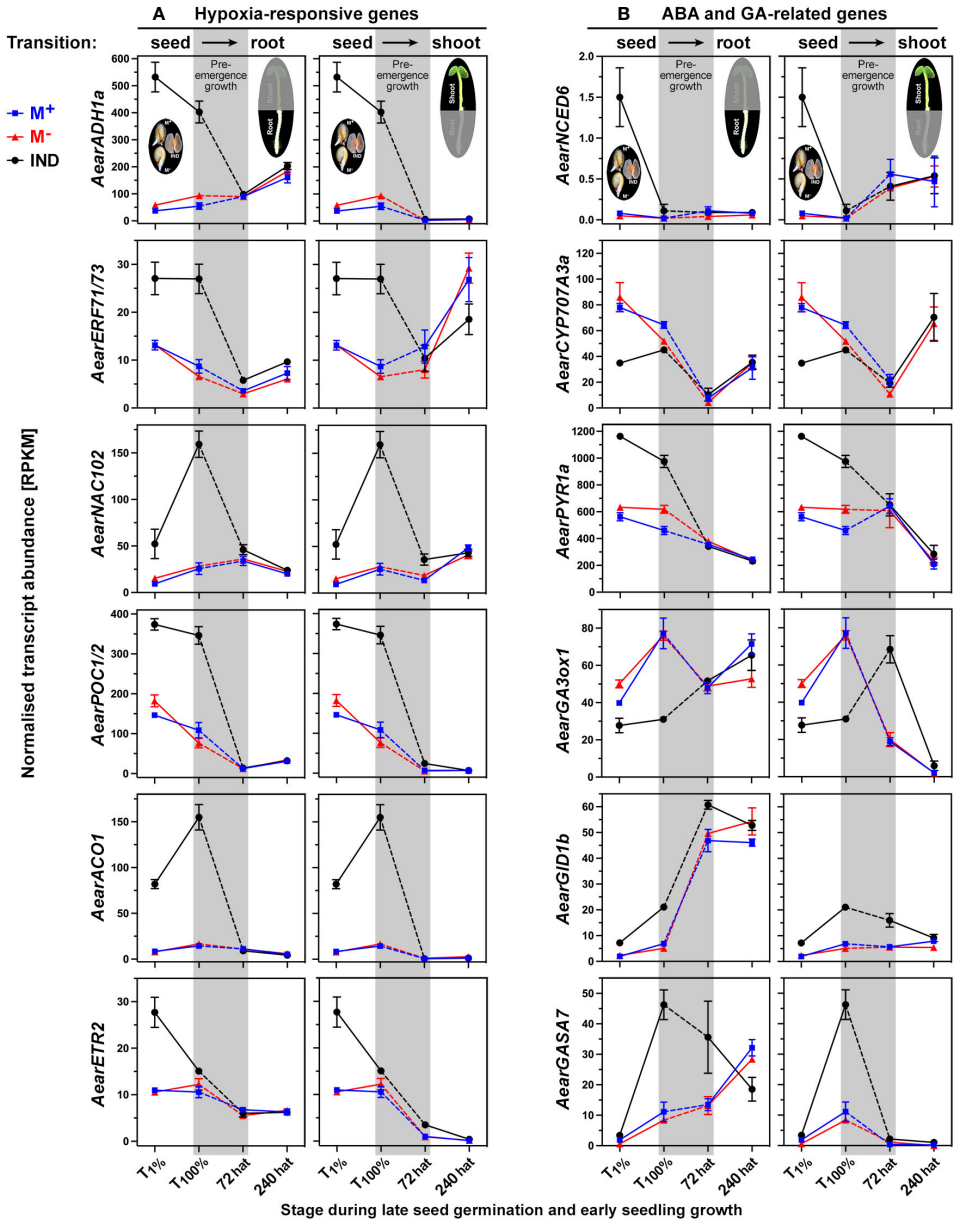
Comparative spatiotemporal analysis of transcript abundance patterns of selected *Aethionema arabicum* differentially expressed genes (DEGs). **(A)** Hypoxia-responsive genes. **(B)** Abscisic acid (ABA) and gibberellin (GA)-related genes. M^+^ seeds, M^−^ seeds, and IND fruits were imbibed in dH_2_O under darkness at 9°C, sampled, and harvested at T_1%_ and T_100%_. Diaspores that had completed germination (1-mm radicle protrusion) were transferred (at 0 hat, hours after transfer) to vertical plates for the seedling growth assay at 30°C in continuous white light (for details, see **Figure 2**). RNA-seq mean ± SEM values of three biological replicates are presented, and each replicate consisted of 90 seeds or tissue (root or shoot) from 12 seedlings. The pre-emergence growth phase leading from germinated diaspores (T_100%_) to seedlings at 72 and 240 hours after transfer (hat) is shaded gray; seed–seedling transition RNA-seq values for roots (*left panels*) and shoots (*right panels*) are presented. *AearPOC1/2* is the cumulative sum of *AearPOC1* plus *AearPOC2* transcript abundances. Seed, root, or shoot tissue at 1% germination (T_1%_), 100% germination (T_100%_), or 72 or 240 hours after transfer (hat) to seedling growth plates was compared. For gene abbreviations and IDs, see **Supplementary Table 2**.

In earlier work, we identified morph-specific expression patterns of hormone-related genes during seed and fruit development (Arshad et al., 2021) and dormancy and germination of the dimorphic diaspores (Chandler et al., 2024). This revealed the importance of ABA metabolism and signaling in dimorphic diaspore development and germination, and key roles for ABA and hypoxia in imposing pericarp-imposed dormancy in IND fruits. **Figure 9A** shows that when IND fruits were compared to M^+^ and M^−^ seeds, the distinct expression patterns of hypoxia-responsive genes in seeds were reset during the transition from the germination (T_100%_) to the early seedling (72 hat) phase. This was evident for the hypoxia-regulated transcription factor (TF) genes, such as *AearNAC102* and *AearERF71/73*, and downstream genes, such as the ethanolic fermentation enzyme gene *AearADH1a* (Chandler et al., 2024). The *Ae. arabicum* genes presented in **Figure 9A** were identified as part of the core hypoxia-responsive gene list derived from hypoxia-treated *Arabidopsis thaliana* seedlings (Christianson et al., 2009; Gasch et al., 2016; Lee and Bailey-Serres, 2019), and the resetting of their expression patterns, therefore, also indicates the absence of hypoxia once the M^−^ seedlings have emerged from the IND pericarp. Most genes involved in ethylene biosynthesis, 1-aminocyclopropane-1-carboxylic acid (ACC) oxidase (ACO) and ACC synthase (ACS), and many genes encoding ethylene response factor (ERF) TFs also exhibited resetting during pre-emergence seedling growth (**Figure 9A**; **Supplementary Figure 5A**). However, for genes encoding the ACC oxidases AearACO2 and AearACO4, the ethylene receptor AearETR2, and several ERF TFs (AearERF2, AearERF11, AearERF113/RAP2.6, and AearRAP2.11), distinct transcript abundances were retained in 72-hat shoots or roots (**Supplementary Figure 5A**). The *A. thaliana* homologs of these ERF TFs are known to be involved in the control of seedling growth by modulating ABA, ethylene, gibberellin, and auxin signaling (Kim et al., 2012; Zhou et al., 2016; Lorrai et al., 2018; Zhu et al., 2020; Templalexis et al., 2022). AtRAP2.11 is, in addition, known as a major regulator of potassium and nitrate transporters in responses to low-nutrient conditions (Kim et al., 2012; Meng et al., 2016; Templalexis et al., 2022).

In contrast to ethylene-related genes, for all ABA-related metabolism and signaling genes, as well as for the PYR/PYL/RCAR-type ABA receptor genes, resetting of their expression was complete in 72-hat seedlings (**Figure 9B**; **Supplementary Figure 5B**). An exception was the ABA-responsive element (ABRE)-binding protein/factor (ABF) *AearAREB3b* for which higher transcript abundances prevailed in 72-hat M^−^ seedling shoots, but this difference disappeared in 240-hat seedlings (**Supplementary Figure 5B**). Morph-specific expression patterns for ABA biosynthesis [e.g., 9-*cis*-epoxycarotenoid dioxygenase (NCED) genes] and ABF-type TFs, and ABA contents were a hallmark during the dimorphic seed/fruit development (Lenser et al., 2018; Arshad et al., 2021) and in imbibed dimorphic diaspores (Chandler et al., 2024). Differences in ABA relations were, therefore, most important between dimorphic diaspores but became less important between M^+^ and M^−^ seedlings since they were reset during pre-emergence seedling growth. In contrast to this, distinct expression patterns for gibberellin (GA)-related genes prevailed in 72-hat seedlings (**Figure 9B**; **Supplementary Figure 6A**). The transcript abundances for the GA 3-oxidase (biosynthesis of bioactive GA) gene *AearGA3ox1* and the *AearGID1b* gene encoding a GA receptor were higher in 72-hat M^−^ seedling shoots derived from IND fruits compared to M^+^ seedling shoots, suggesting that GA biosynthesis and sensitivity differ between the morphs during early seedling growth. Similarly, the transcript abundances of GASA (“GA-stimulated *Arabidopsis*”) genes known to be stimulated by GA and regulated by DELLA repressor proteins (Zhang and Wang, 2017) were higher in 72-hat M^−^ seedling roots derived from IND fruits compared to M^+^ seedling roots, while genes for DELLA repressor proteins did not differ between the morphs (**Figure 9B**; **Supplementary Figure 6A**). In general, ABA has an inhibitory and GA has a promoting role in the complex hormonal control of seedling shoot and root growth (Ahammed et al., 2020). We conclude from the *Ae. arabicum* results that for most genes, resetting occurs during the post-germination pre-emergence growth phase (T_100%_ seeds to 72-hat seedlings) but also that several ethylene and GA-related genes involved in hormonal interactions are among the DEGs in 72-hat seedlings for which resetting occurs later during seedling growth (**Figure 7**).

**Figure 10:**
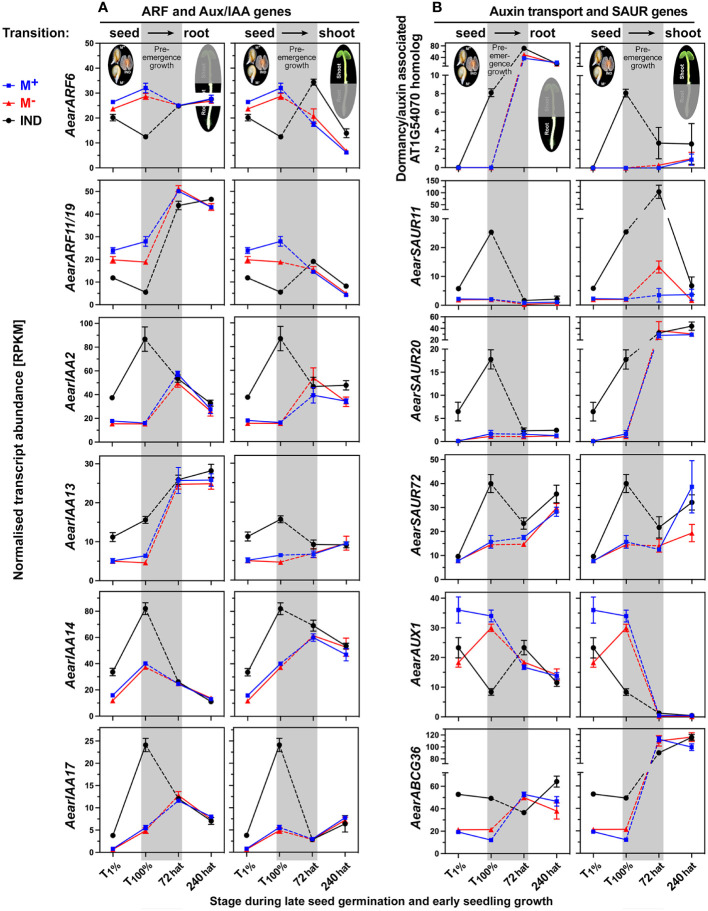
Comparative spatiotemporal analysis of transcript abundance patterns of auxin-related *Aethionema arabicum* differentially expressed genes (DEGs). **(A)** Auxin response factor (ARF) and auxin/indole-3-acetic acid (Aux/IAA) genes. **(B)** Auxin/dormancy-associated and small auxin-upregulated RNA (SAUR) genes. RNA-seq mean ± SEM values of three biological replicates are presented; for details, see **Figure 9** and main text. For gene abbreviations and IDs, see **Supplementary Table 2**.

That *Ae. arabicum* shoot and root development changed during pre-emergence seedling, which was also evident from the chlorophyll accumulation (**Figure 3**), the DEG list comparison (**Figure 7D**), and the shoot-specific induction of chlorophyll-related genes (**Supplementary Figure 7A**). Interestingly, these chlorophyll-related genes were DEGs in 72-hat shoots with lower expression in M^−^ seedlings derived from IND fruits. Ethylene, GA, and ABA interact with auxin to regulate seedling growth differently in shoots and roots (Belin et al., 2009; Hu et al., 2017; Ahammed et al., 2020). Auxin/indole-3-acetic acid (Aux/IAA) proteins (IAAs) repress auxin-inducible genes by inhibiting auxin response TFs (ARFs). In *Ae. arabicum*, several ARFs and Aux/IAA proteins were DEGs during late germination with lower (ARFs) and higher (IAAs) transcript abundances in germinated IND fruits (T_100%_) when compared with germinated M^+^ and M^−^ seeds (**Figure 10A** and **Supplementary Figure 6B**). With the exception of *AearARF6*, which was more highly expressed in IND fruit-derived 72-hat and 240-hat seedling shoots, these expression differences had disappeared in 72-hat seedlings. The *A. thaliana* dormancy/auxin-associated protein is known to be involved in the auxin sensitivity of seedlings (Johnson et al., 2015); its *Ae. arabicum* homolog was more highly expressed in germinated IND fruits (**Figure 10B**). Small auxin-upregulated RNA (SAUR) genes are auxin-responsive genes involved in cell elongation growth and other processes (Stortenbeker and Bemer, 2019). An example in *A. thaliana* seeds is *AtSAUR11*, which accumulates eightfold in the endosperm as compared to the embryo (Dekkers et al., 2013). In general, the differential expression of *Ae. arabicum* SAUR genes during late germination (T_100%_) was reset in 72-hat seedlings (**Figure 10B**; **Supplementary Figure 6B**). A notable exception was *AearSAUR11* for which the transcript abundance was high in germinated IND fruits (T_100%_) and increased further >10-fold in 72-hat M^−^ seedling shoots derived from IND fruit as compared to M^+^ seedling shoots (**Figure 10B**).

### 2.4 Dimorphic expression of transporter genes for water, auxin, nitrate, and flavonoids

**Figure 11:**
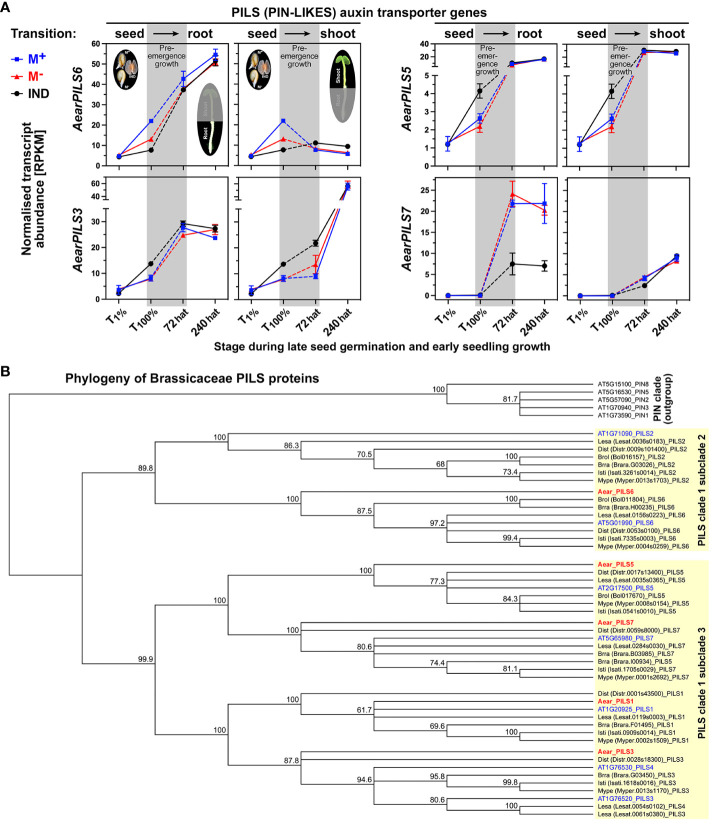
Comparative spatiotemporal analysis of transcript abundance patterns and phylogeny of PILS auxin transporter genes. **(A)** Expression patterns of *Aethionema arabicum PILS* genes. RNA-seq mean ± SEM values of three biological replicates are presented; for details, see **Figure 9**. **(B)** Phylogenetic tree of the predicted amino acid sequences of Brassicaceae PILS (PIN-FORMED-LIKES) auxin efflux carrier. Known and putative amino acid PILS sequences were aligned using ClustalW, and Neighbor-Joining trees were built as described in the Materials and Methods. Naming of PILS sequences was as follows: species as four-letter code (Brra, *Brassica rapa*; Brol, *Brassica oleracea*; Dist, *Diptychocarpus strictus*; Isti, *Isatis tinctoria*; Lesa, *Lepidium sativum*; Mype, *Myagrum perfoliatum*), gene identifier in brackets, and naming based on highest sequence similarity with the *Arabidopsis thaliana* PILS sequences (*in blue*). For *Aethionema arabicum PILS* gene (*in red*) identifier, see **Supplementary Table 2**. Species selection was based on Brassicaceae phylogeny in which *Aethionema* is the sister to all Brassicaceae, *Arabidopsis* and *Lepidium* represent core Brassicaceae lineage I, the two *Brassica* species represent the lineage II Brassicaceae, and *Isatis* and *Myagrum* represent lineage II Isatideae (Franzke et al., 2011).

Polar auxin transport and auxin homeostasis are key to seedling shoot and root growth and are facilitated by tightly regulated efflux [PIN (PIN-FORMED proteins) and PILS (PIN-LIKES)] and influx (AUX1) carriers, which coordinate cell type-specific asymmetric subcellular auxin localization and local auxin gradients across tissues (Belin et al., 2009; Hu et al., 2017; Yi et al., 2021; Bogaert et al., 2022; Feraru et al., 2022; Nakabayashi et al., 2022; Waidmann et al., 2023). Several auxin carriers including *AearAUX1*, *AearABCG36* (**Figure 10B**), *AearPILS5*, and *AearPILS6* (**Figure 11A**) were identified as DEGs during late germination (T_100%_), but their expression difference was reset during seedling pre-emergence growth. In contrast to these auxin transporters, *AearPILS3*, which was also a DEG in germinated diaspores (T_100%_), remained as a DEG in shoots at 72 hat (**Figure 11A**). *AearPILS7* was not expressed during diaspore germination, but it was induced afterward and identified as a DEG in roots during pre-emergence seedling growth. The transcript abundances for *AearPILS7* were lower in 72-hat and 240-hat seedling roots derived from germinated IND fruits as compared to seedling roots derived from germinated M^+^ and M^−^ seeds (**Figure 11A**). In *A. thaliana* seedlings, *AtPILS7* is involved in fine-tuning stress-responsive root auxin signaling in response to phosphate availability and regulation of phosphate transporter gene expression (Yi et al., 2021). In phylogenies across the plant kingdom, the PILS protein sequences of embryophytes plus green algae form a clade distinct from the PIN proteins, and the seven *A. thaliana* and other embryophyte PILS protein sequences are distributed over two PILS subclades 2 and 3 (Feraru et al., 2012; Bogaert et al., 2022). **Figure 11B** shows a family-wide phylogenetic analysis of Brassicaceae PILS protein sequences with the five identified *Ae. arabicum* protein sequences distributed across the two subclades (one in subclade 2 and four in subclade 3). In addition to PIN and PILS efflux carriers, which are specific for indole-3-acetic acid (IAA) transport, ABCG36 is known to act as a plasma membrane located exporter for the IAA precursor indole-3-butyric acid (Geisler et al., 2017; Aryal et al., 2019; Nakabayashi et al., 2022). Based on the *AearABCG36* expression patterns, it is a germination DEG that is reset during pre-emergence growth (**Figure 10B**).

**Figure 12:**
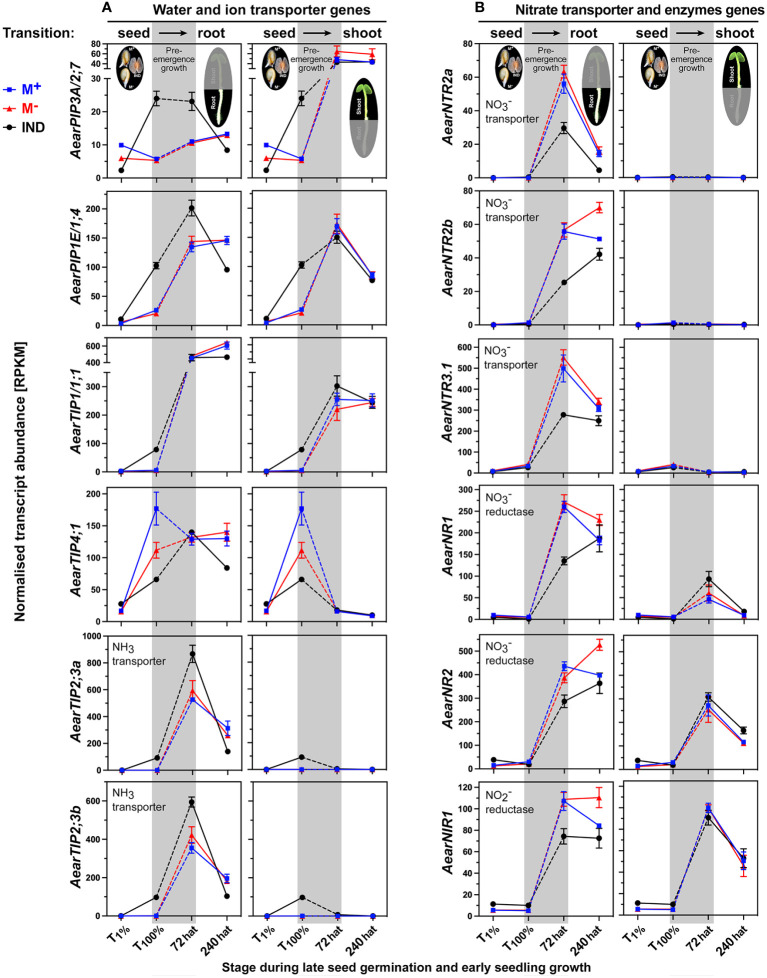
Comparative spatiotemporal analysis of transcript abundance patterns of *Aethionema arabicum* transporter and enzyme differentially expressed genes (DEGs). **(A)** Water and ion transporter genes. **(B)** Nitrate transporter and enzyme genes. RNA-seq mean ± SEM values of three biological replicates are presented; for details, see **Figure 9** and main text. For gene abbreviations and IDs, see **Supplementary Table 2**.

Auxin and other hormones regulate the transition from germination to seedling growth by affecting the expression patterns of water and ion transporters (Yi et al., 2021; Tan et al., 2022; Templalexis et al., 2022). This includes aquaporins, e.g., plasma membrane intrinsic proteins (PIPs) and tonoplast intrinsic proteins (TIPs), transporting water, ammonia (NH_3_), and other solutes (Loqué et al., 2005; Footitt et al., 2019; Hoai et al., 2020). **Figure 12A** shows that the transcript abundances of *AearPIP3A/2;7* and *AearPIP1E/1;4* were more highly expressed in IND fruits during late germination (T_100%_) and remained higher in 72-hat seedling roots derived from IND fruits as compared to seedling roots derived from M^+^ and M^−^ seeds. *AearTIP1,4*, for which the *A. thaliana* homolog is regulated by ABA in seeds (Footitt et al., 2019), and *AearTIP1/1,1* were reset during pre-emergence growth (**Figure 12A**). In contrast to this, expression of the NH_3_ transporter genes *AearTIP2;3a* and *AearTIP2;3b* was higher in IND fruits during late germination (T_100%_) and remained higher in 72-hat seedling roots derived from IND fruits as compared to seedling roots derived from M^+^ and M^−^ seeds (**Figure 12A**). In *A. thaliana* seedlings, root high-affinity nitrate (NO_3_^−^) transporters such as NRT2 interact with polar auxin transport (Wang et al., 2023a). *AearNTR2* and *AearNTR3.1* were not expressed during *Ae. arabicum* germination, and their root-specific induction in 72-hat seedlings was lower in roots derived from IND fruits (**Figure 12B**).

Nitrate and potassium transporter gene expression in *A. thaliana* seedlings is mediated by auxin and the ERF TF RAP2.11 in responses to low-nutrient conditions (Kim et al., 2012; Meng et al., 2016; Templalexis et al., 2022). The expression patterns of *AearRAP2.11* (**Supplementary Figure 5A**) and the transporters for nitrate *AearNTR2* (**Figure 12B**) and potassium *AearKUP3* (**Supplementary Figure 7A**) showed reduced transcript abundances in 72-hat roots derived from IND fruits, which suggest a role of RAP2.11 in the regulation of potassium and nitrate relations during pre-emergence seedling growth. Key enzymes for nitrate assimilation include nitrate reductase (NR) and nitrite reductase (NRI) for which the expression is regulated by hormones and abiotic stresses (Tang et al., 2022). As for the nitrate transporters, also the genes for nitrate assimilation enzymes *AearNR1*, *AearNR2*, and *AearNIR1* were not expressed during germination, and their induction in 72-hat seedling roots was lower in roots derived from IND fruits (**Figure 12B**). This suggests that nitrate transport and assimilation differ in 72-hat roots of seedlings derived from IND fruits and M^+^ seeds and that instead of resetting, a distinct reprogramming occurred.

**Figure 13:**
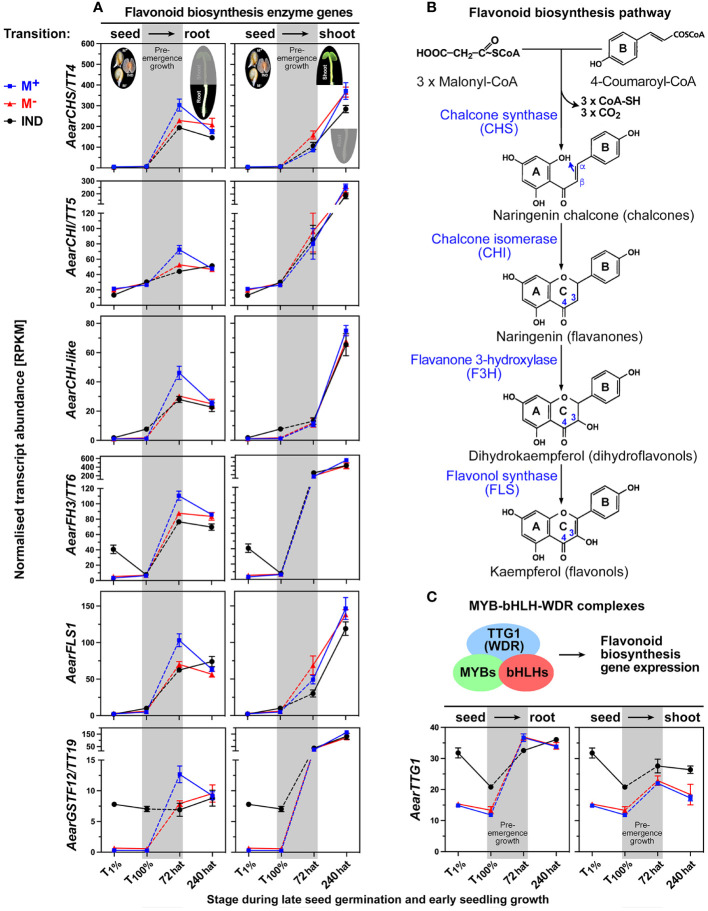
Comparative spatiotemporal analysis of transcript abundance patterns of *Aethionema arabicum* flavonoid biosynthesis pathway genes. **(A)** Flavonoid biosynthesis enzymes and the *GSTF12/TT19* transporter gene for proanthocyanidin precursor molecules. RNA-seq mean ± SEM values of three biological replicates are presented; for details, see **Figure 9** and main text. **(B)** Simplified scheme of the flavonoid biosynthesis pathway. **(C)** Transcriptional control of the flavonoid biosynthesis pathway by MYB-bHLH-WDR complexes. For abbreviations, see main text. For gene abbreviations and IDs, see **Supplementary Table 2**.

The dimorphic expression patterns of genes encoding enzymes of the flavonoid biosynthesis pathway and proanthocyanidin (PA) accumulation are examples of root-specific DEGs during *Ae. arabicum* pre-emergence growth at 72 hat for which the transcript abundances were higher in seedlings derived from M^+^ seeds as compared to seedlings derived from M^−^ seeds or IND fruits (**Figure 13**). In *A. thaliana*, mutations in many of the flavonoid biosynthetic genes as well as in genes of the MYB-bHLH-WDR (MBW) protein complex regulating flavonoid biosynthesis lead to “transparent testa (tt)” mutant phenotypes with reduced seed dormancy (Lepiniec et al., 2006; Macgregor et al., 2015; Xu et al., 2015). *A. thaliana TT19* encodes a glutathione-*S*-transferase (GST)-like protein that functions as a carrier to transport anthocyanin and PA precursors and is involved in the accumulation of PAs in the seed coat (Kitamura et al., 2010; Sun et al., 2012). The *AearGSTF12/TT19* transcript abundances during seed germination were high in IND fruits, while there was no expression during M^+^ and M^−^ seed germination (**Figure 13A**). This expression pattern changed during seedling pre-emergence growth associated with the induction of the flavonoid biosynthetic pathway enzymes, which led to higher expression in 72-hat roots of seedlings derived from M^+^ seeds.

The induction of the flavonoid biosynthesis pathway (**Figure 13B**) genes by the MBW complex was associated with enhanced expression of the WDR protein AearTTG1 (**Figure 13C**). Its expression pattern during germination supports a role in the enhanced expression of *AearGSTF12/TT19* in IND fruits, but it does not explain the higher expression of *AearGSTF12/TT19* and the flavonoid biosynthesis pathway enzymes in M^+^ seed-derived seedling roots at 72 hat (**Figure 13A**). No bHLH and no MYB TF with enhanced expression in M^+^ seed-derived seedling roots at 72 hat were identified. Several *Ae. arabicum* MYB TFs were identified as DEGs during late seed germination (T_100%_) with higher expression in IND fruits compared to imbibed M^+^ and M^−^ seeds, but for all of them, resetting occurred during pre-emergence growth (**Supplementary Figures 8A, B**). Among these DEGs is MYB30, which is a key TF in *A. thaliana* seeds and seedlings integrating ABA, ethylene, and reactive oxygen species (ROS) signaling (Mabuchi et al., 2018; Khedia et al., 2019; Maki et al., 2019; Nie et al., 2022; Zhang et al., 2023). Other DEGs are presented and discussed in **Supplementary Figure 7B**.

### 2.5 Resetting of maturation and dormancy genes, TFs, and seed–seedling transition markers

**Figure 14:**
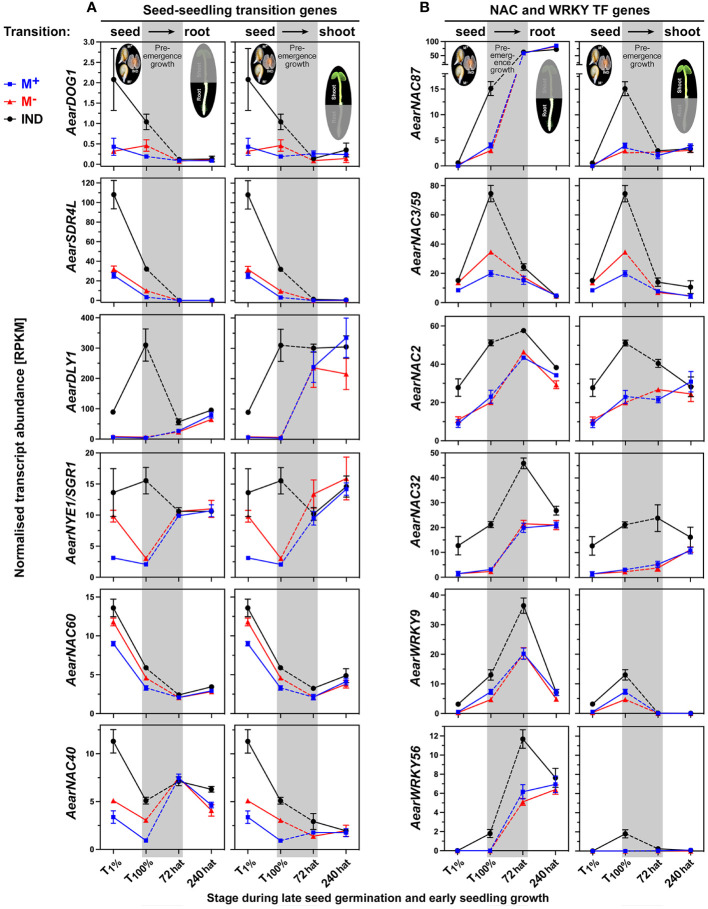
Comparative spatiotemporal analysis of transcript abundance patterns of *Aethionema arabicum* differentially expressed genes (DEGs). **(A)** Seed–seedling transition genes. **(B)** NAC and WRKY transcription factor (TF) genes. RNA-seq mean ± SEM values of three biological replicates are presented; for details, see **Figure 9** and main text. For gene abbreviations and IDs, see **Supplementary Table 2**.

Dormancy and maturation gene expression usually decline during the germination of non-dormant seeds. In *A. thaliana* and other species, the transcript abundances for the *Delay of Germination 1* (*DOG1*) and *Seed Dormancy 4-Like* (*SDR4L*) decline in imbibed non-dormant seeds (Graeber et al., 2014; Wu et al., 2022), while *Dormancy-associated protein Like 1* (*DLY1*) and *Non-Yellowing 1/Stay-Green 1* (*NYE1/SGR1*) exhibit more complex expression patterns during seed imbibition (Rae et al., 2014; Wilhelmsson et al., 2019). The transcript abundances of *AearDOG1*, *AearSDR4L*, *Aear DLY1*, and *Aear NYE1/SGR1* were higher in imbibed IND fruits as compared to imbibed M^+^ and M^−^ seeds and declined for *AearDOG1* and *AearSDR4L* (**Figure 14A**). The observed expression difference during germination for all four genes disappeared during pre-emergence growth, and this resetting led to roughly equal transcript abundances in 72-hat seedlings.

Germin (GER) and germin-like proteins are expressed in seeds, but their functions are largely unknown (Membre et al., 2000). In *Ae. arabicum*, *AearGER3* is a DEG during late germination, but not in seedlings, where it is expressed in a shoot-specific manner (**Supplementary Figure 9A**). Late embryogenesis-abundant (LEA) proteins accumulate late in seed development and play major roles in desiccation tolerance (Hundertmark and Hincha, 2008; Zinsmeister et al., 2020; Smolikova et al., 2022). Most genes encoding LEA proteins are known to be ABA-induced, and their transcript abundances decline during seed germination. Consistent with the ABA inducibility and higher ABA contents in IND fruits (Chandler et al., 2024), the transcript abundances of *Ae. arabicum LEA* genes were higher in imbibed IND fruits as compared to M^+^ and M^−^ seeds (**Supplementary Figure 9**). Expression patterns of specific LEA and Heat Shock Protein (HSP) genes are presented in detail in **Supplementary Figure 9**.

For most *Ae. arabicum* TFs, which were identified as DEGs between IND fruit and M^+^ seed diaspores during the germination phase, resetting of the expression pattern was observed during the post-germination pre-emergence phase (**Supplementary Figure 8**). Examples where the expression differences during late germination (T_100%_) persisted or were even intensified into the seedling phase include the auxin and ethylene-related TF genes *AearARF6* and *AearERF113* presented earlier (**Figure 10A**; **Supplementary Figure 5A**). Resetting during the post-germination pre-emergence phase was also observed for NAC (NAM, ATAF, and CUC) TFs involved in hypoxia responses (*AearNAC102*, **Figure 9A**) and for *AearNAC40* and *AearNAC60* (**Figure 14A**), which, in *A. thaliana*, are functionally redundant in the inhibition of dormancy (Song et al., 2022). Homologs of *AearNAC3/59* (**Figure 14B**) and *AearNAC87* (**Figure 14B**) are known for being involved in the primary root growth of *A. thaliana* seedlings, for being expressed in the endosperm during germination (Dekkers et al., 2013), and for controlling programmed cell death during root growth (Huysmans et al., 2018). In contrast to these NAC TFs, higher transcript abundances in IND fruit diaspores during germination were maintained for *AearNAC2* and *AearNAC32*, and this difference was further intensified at the 72-hat seedling state (**Figure 14B**). In *A. thaliana*, NAC2 has a role in integrating environmental and hormone (auxin and ethylene) stimuli during seedling root growth (He et al., 2005) and integration of auxin signaling (Park et al., 2011), and NAC32 has a role in upstream TF in the control of seedling root elongation and ROS signaling (Maki et al., 2019).

The WRKY9 and WRKY40 TFs are known as central repressors of ABA signaling during *A. thaliana* seed germination and seedling growth (Wang et al., 2021) and of ROS and stress signaling (Shin and Schachtman, 2004; Van Aken et al., 2013; Arjmand et al., 2023). In *Ae. arabicum*, *AearWRKY9* and *AearWRKY40* were identified as DEGs more highly expressed in germinating IND fruits as compared to M^+^ seeds, and this expression pattern was retained in 72-hat seedlings (**Figure 14B**, **Supplementary Figure 8C**). WRKY51 and WRKY56 belong to a subgroup of WRKY TFs involved in controlling auxin transport during *A. thaliana* root development (Templalexis et al., 2022). *AearWRKY51* and *AearWRKY56* are DEGs with higher expression in IND fruits during the germination of the *Ae. arabicum* dimorphic diaspores (**Figure 14A**, **Supplementary Figure 8C**). While resetting of this expression difference during pre-emergence growth was observed for *AearWRKY51*, it was retained and further intensified for *AearWRKY56* in 72-hat seedling roots (**Figure 14B**). Expression patterns of other TFs including homeobox TFs controlling seed-to-seedling phase transition node regulators and the plant-specific AT-rich sequence zinc-binding protein (PLATZ) TFs are presented in detail in **Supplementary Figure 8**. Taken together, both resetting and intensification of differential expression patterns were observed during the seed-to-seedling transition for TFs from several distinct gene families. For the TFs that were identified as DEGs at the seedling stage expression, the transcript abundances were always higher in seedlings derived from IND fruits and lower in seedlings derived from M^+^ seeds.

### 2.6 Resetting of cell wall remodeling protein gene expression during seed–seedling transition

**Figure 15:**
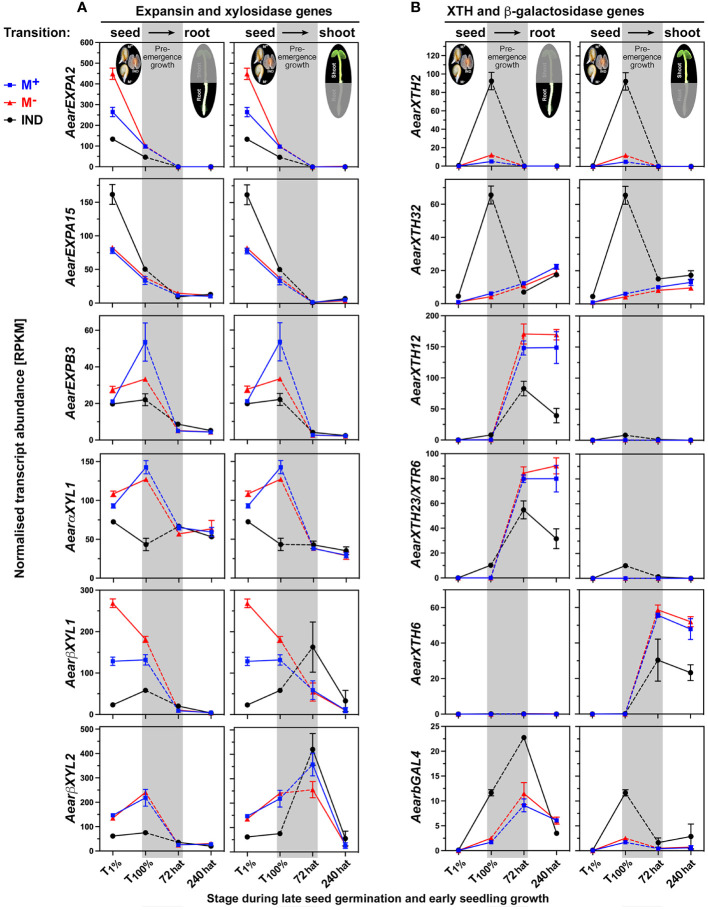
Comparative spatiotemporal analysis of transcript abundance patterns of *Aethionema arabicum* differentially expressed genes (DEGs) encoding cell wall remodeling proteins. **(A)** Expansin and xylosidase genes. **(B)** Xyloglucan endotransglucosylase/hydrolase (XTH) and β-galactosidase genes. RNA-seq mean ± SEM values of three biological replicates are presented; for details, see **Figure 9** and main text. For gene abbreviations and IDs, see **Supplementary Table 2**.

Cell expansion growth is driven by water uptake, which is restricted unless cell wall loosening is achieved by the action of cell wall remodeling proteins (CWRPs) or apoplastic ROS (Finch-Savage and Leubner-Metzger, 2006; Steinbrecher and Leubner-Metzger, 2017; 2018). Expansins are CWRPs that disrupt non-covalent bonds that tether cell wall matrix polysaccharides to the surface of cellulose microfibrils or each other. Expansins are required for endosperm weakening and embryo elongation during germination and seedling growth (Voegele et al., 2011; Graeber et al., 2014; Boron et al., 2015; Cosgrove, 2016; Ilias et al., 2019). In agreement with the slower germination of *Ae. arabicum* IND fruits as compared to M^+^ seeds, the expression of *AearEXPA2* and most other expansins was lower in imbibed IND fruits (**Figure 15A**; **Supplementary Figure 10A**). Resetting during post-germination pre-emergence growth was observed for most expansins, which were DEGs during germination, but other expansins also exhibited differential expression in seedling roots. Xyloglucan remodeling enzymes involved in seed germination include α-xylosidase (αXYL) (Shigeyama et al., 2016), β-xylosidase (βXYL) (Arsovski et al., 2009), and xyloglucan endotransglucosylase/hydrolase (XTH) (Voegele et al., 2011; Endo et al., 2012; Graeber et al., 2014; Steinbrecher and Leubner-Metzger, 2017; Holloway et al., 2021). In agreement with roles in imbibed *Ae. arabicum* dimorphic diaspores, *AearαXYL1*, *AearβXYL1*, and *AearβXYL2* are DEGs during germination with lower expression in IND fruits (**Figure 15A**). Resetting during post-germination pre-emergence growth leads to roughly similar expression in seedlings derived from germinated IND fruits and M^+^ seeds.

Earlier work by Chandler et al. (2024) demonstrated that during early germination (until T_1%_), the cumulative expression of all XTHs in imbibed *Ae. arabicum* dimorphic diaspores is lower in IND fruits compared to M^+^ and M^−^ seeds. Interestingly, the transcript abundances of many *AearXTH* genes increased in IND fruits during the late germination (until T_100%_), and these were, therefore, identified as germination DEGs (**Figure 15B**; **Supplementary Figure 10A**). In general, resetting during post-germination pre-emergence growth was observed for XTH genes, but *AearXTH12*, *AearXTH23*, *AearXTH31*, and *AearXTH26* were DEGs during seedling growth, and their transcript abundances were lower in 72-hat seedling roots derived from germinated IND fruits compared to seedling roots derived from M^+^ or M^−^ seeds. In *A. thaliana* seedlings, *AtXTH23* is known to be involved in root development and adaptation to salt stress (Xu et al., 2020), gene induction is induced by touch and darkness (Lee et al., 2005), and *AtXTH12* and *AtXTH26* exhibit only endotransglucosylase activity (Maris et al., 2010). In contrast to *AearXTH2*, *AearXTH23*, *AearXTH26*, and *AearXTH31*, the transcript abundances of *AearXTH24* were higher in 72-hat seedling roots derived from germinated IND fruits (**Figure 15B**; **Supplementary Figure 10A**). In *A. thaliana* seedlings, overexpression of AtXTH24 is known for its role in promoting hypocotyl growth of etiolated seedlings (Dhar et al., 2022). In *A. thaliana* seedlings, *AtXTH6* is known to be regulated by ABA and auxins (Overvoorde et al., 2005; Huang et al., 2007). In *Ae. arabicum* seedlings, *AearXTH6* is specifically expressed in seedling shoots and lower in 72-hat and 240-hat seedling shoots derived from germinated IND fruits (**Figure 15B**).

β-Galactosidases have β-1,4-galactose in xyloglucan side chains as targets (Steinbrecher and Leubner-Metzger, 2017; Moneo-Sanchez et al., 2019; Steinbrecher and Leubner-Metzger, 2022). In *Ae. arabicum*, resetting during post-germination pre-emergence growth was not observed for *AearβGAL4*, and the expression remained higher in 72-hat seedlings derived from IND fruits (**Figure 15B**). In *A. thaliana* seedlings, βGAL4’s involvement in cell wall changes is associated with the cessation of cell elongation and increased rigidity (Moneo-Sánchez et al., 2016). β-Galactosidases also have β-1,4-galactan in cell wall pectin (rhamnogalacturonan) as a target. Pectins are complex cell wall matrix polysaccharides characterized by α-1,4-linked galacturonic acid and a dynamic degree of methylesterification, and β-galactosidase and various other cell wall remodeling enzymes target pectin (Cao, 2012; Daher and Braybrook, 2015; Scheler et al., 2015). Their expression patterns in *Ae. arabicum* are presented in detail in **Supplementary Figure 10**. Taken together, CWRP gene expression in the dimorphic diaspore model *Ae. arabicum* revealed either resetting or distinct expression patterns (as DEG in seedlings) during the seed–seedling transition.

## 3 Discussion

### 3.1 The dimorphic diaspore syndrome and distinct seedling responses to abiotic stresses

The hormonal interactions during seed germination/dormancy (GA-ABA antagonism dominant) and early seedling growth (auxin-ethylene interactions dominant) differ fundamentally (Finch-Savage and Leubner-Metzger, 2006; Linkies and Leubner-Metzger, 2012; Hu et al., 2017; Ahammed et al., 2020; Smolikova et al., 2022; Wang et al., 2023b). Seedlings during pre-emergence growth may encounter increasing post-germination stress in the soil environment, and consequently, seeds/seedlings often fail to establish during this stage (Moles and Westoby, 2006; Finch-Savage and Bassel, 2016; Gardarin et al., 2016). It is clear from many dimorphic diaspore systems that the germination/dormancy traits of the two distinct morphs differ in their responses to environmental cues including abiotic stresses and that the underpinning molecular (hormonal, transcriptomic, and epigenetic) mechanisms differ (Xu et al., 2011; Zhou et al., 2015; Lu et al., 2015a, b; Lenser et al., 2016; Wilhelmsson et al., 2019; Arshad et al., 2021; Gianella et al., 2021; Zhang et al., 2021; Cao et al., 2022; Loades et al., 2023; Song et al., 2023; Chandler et al., 2024). Whether or not seedlings derived from dimorphic diaspores differ in their molecular responses has been far less investigated. Very little is known about if and when these differences disappear by resetting during seedling growth and whether or not these seedling differences were already induced during the dimorphic seed development on the mother plant and then retained during imbibition.

Comparative analysis of seedlings derived from *Atriplex* and *Suaeda* dimorphic seeds that were distinct in seed color revealed that seedlings were also distinct in responses to salinity and certain molecular features (Xu et al., 2011; Zhang et al., 2021; Cao et al., 2022; Song et al., 2023). Among the enriched functional gene categories that differed in the seedling transcriptomes were genes encoding inorganic ion transport, hormone metabolism, transport and signaling, TFs and signal transduction components, CWRPs, lipid metabolism, redox, and osmotic homeostasis. In these systems, dimorphic seeds differing in color, size, and dormancy were dispersed by pod shattering from dehiscent fruits. A conclusion from these publications is that the observed differences in salinity tolerance of the dimorphic seedlings were already initiated during seed development on the mother plant. Compared to this, the situation in the *Ae. arabicum* dimorphic diaspore system is different in that seedlings derived from either M^+^ or bare M^−^ seeds did not show any obvious differences in a range of constant temperature or osmotic stress (**Figure 2**; **Supplementary Figures 3**, **4**). What is different in the *Ae. arabicum* M^+^ seed and the IND fruit morph system is the presence of the pericarp, which is known to impose coat dormancy and delay the germination of imbibed IND fruits (Lenser et al., 2016; Chandler et al., 2024).

Pericarp removal experiments with monocarpic species demonstrated that beyond seed traits, the pericarp can also affect seedling establishment and performance (Hu et al., 2009; Mamut et al., 2014; Zhou et al., 2015; Lu et al., 2015b; Lu et al., 2017b; a; Ignatz et al., 2019). Interestingly, in *Ae. arabicum*, the pericarp restraint led to different growth of hypocotyls derived from imbibed IND fruits as compared to M^+^ and M^−^ seedlings derived from M^+^ and bare M^−^ seeds (**Figure 4**). This pericarp effect on pre-emergence seedling growth delivered a 5–20-fold higher number of DEGs from the M^+^/IND and M^−^/IND comparisons (**Figures 7**, **8**) as compared to the 23 DEGs obtained in the M^+^/M^−^ seedling comparison. The pericarp, therefore, plays an important role in the phenotypic plasticity of the *Ae. arabicum* dimorphic diaspores and has a significant downstream effect on the root and shoot transcriptomes of M^−^ seedlings derived from IND fruits. Details about the identified *Ae. arabicum* DEGs were already described in the Results section. The following discussion, therefore, focuses on general aspects and selected major functional DEG categories of the transcriptome resetting process.

### 3.2 Transcriptome resetting during pre-emergence growth and DEG persistence in seedlings

For the majority of the 1,900–2,000 M^+^/IND and M^−^/IND DEGs at the completion of germination (T_100%_), resetting occurred during pre-emergence seedling growth and is completed in 72-hat seedlings (**Figures 7**, **8**). Examples of this include all hypoxia-responsive and many hormone-related genes (see Section 2.3), as well as dormancy, maturation, LEA, and HSP genes (see Section 2.5). However, a considerable number of ethylene, GA, and auxin-related genes either persisted as a DEG in 72-hat seedling or developed into a DEG during pre-emergence seedling growth (T_100%_ to 72-hat seedling roots or shoots). This includes enhanced transcript expression for the ethylene-forming enzyme gene *AearACO2* in 72-hat seedlings derived from IND fruits (**Supplementary Figure 5**), GA-related genes including encoding the bioactive GA_4_-forming enzyme *AearGA3ox1*, the GA receptor *AearGID1b* (**Figure 9B**), and several auxin-related genes including *AearSAUR11* (**Figure 10B**). Ethylene, GA, and ABA interact with auxin to regulate seedling growth differently in shoots and roots (Belin et al., 2009; Hu et al., 2017; Ahammed et al., 2020; Wang et al., 2023b). ACO2 is known for its role in counteracting ABA effects in seeds (Linkies et al., 2009; Linkies and Leubner-Metzger, 2012) and in promoting apical hook formation in seedling shoots (Wang et al., 2023b), and ethylene mediates the ABA inhibition on seedling root growth (Ahammed et al., 2020). Flavonoid biosynthesis pathway genes (**Figure 13**) exhibited a distinct expression pattern from most other DEGs with a root-specific upregulation in 72-hat M^+^ seedlings derived from germinated M^+^ seeds. The role of flavonoids in M^+^ seedlings as compared to M^−^ seedlings and the distinct regulation by MBW protein complexes remain to be elucidated by future research.

ERF and ARF TFs are involved in the control of seedling growth by modulating ABA, ethylene, gibberellin, and auxin signaling (Kim et al., 2012; Zhou et al., 2016; Lorrai et al., 2018; Zhu et al., 2020; Templalexis et al., 2022). In agreement with the role of distinct hormonal signaling in M^+^ and M^−^ *Ae. arabicum* seedlings, transcripts for *AearARF6* and several ERF TFs are DEGs in 72-hat seedling roots or shoots (**Figure 9**; **Supplementary Figure 5**). ARF and ERF TFs were also identified as DEGs in seedlings derived from the dimorphic (black versus brown) seeds of *Suaeda aralocaspica* (Cao et al., 2022). Resetting for the majority of the *Ae. arabicum* TF DEGs during pre-emergence seedling growth was completed at 72 hat (**Figure 14**; **Supplementary Figure 8**). Notable exceptions were two NAC (*AearNAC2* and *AearNAC32*) and two WRKY (*AearWRKY9* and *AearWRKY40*) TF genes for which higher transcript abundances in IND fruit diaspores during germination persisted at the 72-hat seedling state (**Figure 14B**). In *A. thaliana* seedling growth, these TFs are involved in integrating environmental and hormonal stimuli (see Section 2.5). Taken together, transcriptomes of M^+^ and M^−^ *Ae. arabicum* seedlings derived from germinated M^+^ seeds and IND fruits differ in the expression of genes involved in ethylene and GA metabolism, hormone signaling, and for various TFs with roles in integrating environmental and hormonal stimuli.

### 3.3 Dimorphic expression patterns of auxin/ion transporter and CWRP genes in seedlings

Precise auxin distribution patterns and polar transport are required for the control of root and hypocotyl growth and development (Teale et al., 2006; Hu et al., 2017; Wang et al., 2023b). The PILS auxin carriers are known to be involved in intracellular auxin homeostasis (Feraru et al., 2012, 2019; Bogaert et al., 2022; Feraru et al., 2022). In *Ae. arabicum*, several auxin carriers were DEGs during late germination (T_100%_), but their expression difference was reset during seedling pre-emergence growth (**Figures 9**, **10**). *AearPILS3*, in addition, remained a DEG at 72 hat where it was more highly expressed only in seedling shoots derived from IND fruits. *AearPILS7* became a root-specific DEG at 72 and 240 hat, where it was more highly expressed only in seedling roots derived from IND fruits. Seedlings derived from IND fruits differed from M^+^ and M^−^ seedlings derived from germinated M^+^ seeds and bare M^−^ seeds, respectively, in that their lower hypocotyls were often bent and not straight and that their hypocotyl and root growth was slower (**Figure 4**). We speculate that differences in auxin biosynthesis, signaling, and transport play a major role in this altered seedling growth phenotype. This phenotype was, however, not connected with a difference in hypocotyl mechanics (fracture force) of 15-day-old *Ae. arabicum* seedlings (**Figure 4**). Microtensile measurements of *A. thaliana* seedlings (Saxe et al., 2016) demonstrated that hypocotyls differed mechanically between early (day 4) and later (day 5–7) seedlings, but there were no differences among the later seedlings (Saxe et al., 2016). It seems, therefore, that despite the slower growth and the bent hypocotyls of IND fruit-derived *Ae. arabicum* seedlings, possible mechanical differences between pre-emergence (72 hat) have already disappeared in later (>240 hat) seedlings.

Downstream of the hormonal regulation are distinct expression patterns in seedlings derived from IND fruits for water and ion transporters (**Figure 12**) potentially affecting the turgor pressure and for CWRP genes (**Figure 15**) potentially affecting the cell wall extensibility. The rapid and uniform seedling growth depends on cell expansion, which requires cell wall loosening by the action of CWRPs or apoplastic ROS (Finch-Savage and Leubner-Metzger, 2006; Steinbrecher and Leubner-Metzger, 2017; 2018). Among the CWRP DEGs in *Ae. arabicum* seedlings were genes encoding enzymes that target xyloglucan (βGAL4 and XTHs) and pectin (**Figure 15**, **Supplementary Figure 10**). *AearXTH12*, *AearXTH23*, and *AearXTH6* were less expressed in 72-hat and 240-hat seedlings derived from IND fruits as compared to M^+^ and M^−^ seedlings derived from germinated M^+^ and M^−^ seeds. XTHs are known to affect seedling vigor (Ducatti et al., 2022) and hypocotyl growth (Miedes et al., 2013; Dhar et al., 2022), and *AtXTH6* is known to be regulated by ABA and auxins in seedlings (Overvoorde et al., 2005; Huang et al., 2007). Morph-specific differential expression of other xyloglucan remodeling enzymes was observed during *Ae. arabicum* fruit and seed development (Steinbrecher and Leubner-Metzger, 2022), dimorphic diaspore germination (Chandler et al., 2024), and dimorphic seedling growth (this work). CWRP gene expression in the dimorphic diaspore model *Ae. arabicum*, therefore, revealed either resetting or distinct expression patterns (as DEG in seedlings) during seed–seedling transition. Taken together, we conclude that the transcriptomes of seedlings derived from the dimorphic diaspores, M^+^ seeds and IND fruits, undergo transcriptional resetting during the post-germination pre-emergence growth transition phase from germinated diaspores to growing seedlings.

## 4 Materials and methods

### 4.1 Plant material and germination assays

Plants of *Ae. arabicum* (L.) Andrz. ex DC. were grown from accessions TUR ES1020 (from Turkey) as described by Chandler et al. (2024). Mature M^+^ seeds and IND fruits were harvested, further dried over silica gel for 1 week, and stored for a few months at −20°C in air-tight containers. For germination assays, dry mature seeds (M^+^ or M^−^) or IND fruits were placed in 3-cm Petri dishes containing two layers of filter paper, 3 mL deionized water (dH_2_O), and 0.1% Plant Preservative Mixture (Plant Cell Technology, Washington, DC, USA). Temperature response profiles (**Supplementary Figure 1**) were obtained by incubating plates on a GRD1-LH temperature gradient plate device (Grant Instruments Ltd., Cambridge, UK). Subsequent germination assays were conducted by incubating plates in MLR-350 Versatile Environmental Test Chambers (Sanyo-Panasonic, Bracknell, UK) at the indicated imbibition temperature as described by Chandler et al. (2024). Seed germination, scored as radicle emergence, of three biological replicates of 30 seeds or fruits was analyzed.

### 4.2 Seedling growth assays

Seedling growth assays were conducted using just-germinated seeds (1-mm radicle protrusion visible; obtained from surface-sterilized seeds germinated in darkness at 9°C), which were selected for transfer to 12 cm × 12 cm plates containing media based on 1-mm protrusion of the radicle. As medium autoclaved 1% (w/v) agar in 1/10 Murashige and Skoog (MS) basal medium (M5519, Sigma, Darmstadt, Germany) was used. For the seedling growth assays, plates were incubated vertically in constant white light (170 µmol·m^−2^·s^−1^) in MLR-350 Versatile Environmental Test Chambers (Sanyo-Panasonic, Bracknell, UK) at a constant temperature, as indicated (**Figure 2**; **Supplementary Figure 2**). For seedling growth assays during osmotic stress (**Supplementary Figure 3**), water potentials were lowered using high-molecular-weight polyethylene glycol (PEG6000; 26603.293, VWR, Radnor, PA, USA) using an overlay method (Van Der Weele et al., 2000; Verslues and Bray, 2004). Seedling growth assay constant temperatures were 14°C for osmotic stress, and temperatures for the thermal stress experiments were between 14°C and 35°C.

### 4.3 Biochemical and biomechanical analyses

The chlorophyll content of seedlings grown horizontally at 30°C was determined after extracting pigments from leaf tissues homogenized in methanol at room temperature for 15 min while shaking at 1,000 rpm on a thermomixer (S8012-0000, Starlab, Milton Keynes, UK). The obtained extracts were centrifuged for 5 min at 14,000 *g*. The absorbance of the supernatants was determined at 750 nm, 665 nm, and 652 nm using a microplate reader (Spark^®^ 10M, Tecan, Zürich, Switzerland) and subsequently used to calculate chlorophyll contents (Porra et al., 1989). For the biomechanical analyses (Steinbrecher and Leubner-Metzger, 2017), just-germinated *Ae. arabicum* seeds (M^+^ and M^−^) and IND fruits were transferred to agar plates as described above and grown for 15 days under constant white light at 30°C. IND fruits were manually split open just after the completion of germination (radicle protruding the pericarp) at 0 hat. To conduct biomechanical analyses of hypocotyls, seedlings were clamped, leaving a 7-mm gap between the jaws of a single-column tensile testing machine (Zwick Roell ZwickiLine Z0.5, Ulm, Germany). A constant speed for separation was set at 5 mm/min. Force-displacement data were obtained, and hypocotyl breaking forces were calculated (**Figure 4**).

### 4.4 RNA extraction for RNA-seq transcriptome analysis

A sampling of imbibed M^+^ seeds, M^−^ seeds, and IND fruits for molecular analyses was described by Chandler et al. (2024). Three biological replicates of samples each corresponding to 20-mg dry weight of seed material were pulverized in liquid N_2_ using mortar and pestle. Extraction of total RNA was performed as described by Graeber et al. (2011). Sampling and RNA extraction were performed of root and shoot tissue from seedlings grown at 30°C (grown from pre-germinated seeds at 9°C in darkness). Tissue was homogenized at 6,500 rpm using a Precellys 24 (Bertin Instruments, CNIM Group, Paris, France). Seed and shoot total RNA was isolated using a protocol modified by Chang et al. (1993). After the addition of RNA extraction buffer [2% (w/v) hexadecyltrimethylammonium bromide (CTAB), 2% (w/v) polyvinylpolypyrrolidone (PVP), 100 mM Tris-HCl pH 8.0, 25 mM ethylenediaminetetraacetic acid (EDTA), 2 M NaCl, and 2% (v/v) β-mercaptoethanol], samples were incubated at 65°C for 10 min with intermittent vortexing. Chloroform:isoamylalcohol (24:1) extractions were repeated three times. After the addition of 10 M LiCl to a final concentration of 2 M, RNA was precipitated overnight at 4°C and then dissolved in NaCl-Tris-EDTA (STE) buffer [1 M NaCl, 0.5% (w/v) sodium dodecyl sulfate (SDS), 10 mM Tris-HCl pH 8.0, and 1 mM EDTA]. Three further chloroform:isoamylalcohol (24:1) extractions were then performed before precipitation in 100% (v/v) ethanol overnight at −80°C. Samples were then centrifuged for 20 min at 4°C. After the removal of the aqueous phase, the RNA pellets were washed with 70% (v/v) ethanol. Samples were centrifuged for 20 min, the ethanol was carefully removed, and the RNA was subsequently dissolved in RNase-free water. Genomic DNA was removed by DNase-I (QIAGEN, Valencia, CA, USA) digestion in solution, followed by additional purification using columns (QIAGEN RNeasy Kit). Shoot tissue RNA was isolated using the RNeasy Plant Mini Kit (QIAGEN) and manufacturer’s instructions. RNA quantity and purity were determined using a NanoDrop™ spectrophotometer (ND-1000, ThermoScientific™, Wilmington, DE, USA) and an Agilent 2100 Bioanalyzer with the RNA 6000 Nano Kit (Agilent Technologies, Santa Clara, CA, USA) using the 2100 Expert Software to calculate RNA Integrity Number (RIN) values.

### 4.5 Analysis of RNA-seq transcriptome data

Transcriptome assembly, data trimming, filtering, read mapping, feature counting, and DEG detection were performed using the pipeline previously described by Wilhelmsson et al. (2019). PCA was performed using R (R Core Team, 2021) and the Bioconductor package DESeq2 (Love et al., 2014) and plotPCA on log(x + 1)-transformed RPKM values with non-zero values in at least one sample. GO term enrichment in DEG lists was calculated with R package topGO using the elim method with Fisher’s exact test (Alexa and Rahnenfuhrer, 2021). Gene identifiers and symbols are according to earlier publications of the *Ae. arabicum* genome (version 2.5) and transcriptome (Wilhelmsson et al., 2019; Arshad et al., 2021; Chandler et al., 2024), and the *Ae. arabicum* web portal (https://plantcode.cup.uni-freiburg.de/aetar_db/index.php) links this to the current (Fernandez-Pozo et al., 2021) and future genome DB and gene expression atlas.

### 4.6 Phylogenetic analysis

To identify Brassicaceae PILS genes (**Figure 11**), the *A. thaliana* sequences (Yi et al., 2021; Bogaert et al., 2022; Waidmann et al., 2023) were used, and BLAST analyses were conducted via Phytozome (Goodstein et al., 2012). The combined information of the BLAST analyses was used to conduct the phylogenetic analysis with known and putative PILS amino acid sequences aligned using ClustalW (BLOSUM cost matrix, Gap open cost 10, Gap extend cost 0.1), and Neighbor-Joining trees (Saitou and Nei, 1987) were built using Geneious 8.1.9 Tree Builder (Geneious, San Diego, CA, USA) using Jukes-Cantor distance. Consensus support (minimum 20%) was determined using bootstrap (1,000).

### 4.7 Statistical analysis

Data are expressed as mean ± 1 SEM. Statistical analysis of experiments was performed using the GraphPad Prism software (v.7.0a; San Diego, CA, USA) for the analysis of variance (ANOVA) and unpaired t-test procedures. For studies examining abiotic stress effects, data were analyzed by two-way ANOVA, with seedling morph and seedling age (time) as between-group factors. Multiple comparisons were performed using Sidak’s *post-hoc* correction in GraphPad Prism. Results were considered statistically significant if the p-value was less than 0.05.

## Data availability statement

The RNAseq data are deposited at the NCBI Sequencing Read Archive (SRA), BioProject PRJNA639399, https://www.ncbi.nlm.nih.gov/bioproject/PRJNA639399; metadata about the RNAseq samples are also available in Supplementary Dataset 1. Original germination and biomechanics datasets are available at figshare https://doi.org/10.17637/rh.24856305.v2. All other data presented or analyzed in this published article, including accession number(s), are available online through the **Supplementary Material**.

## Author contributions

WA: Conceptualization, Data curation, Formal analysis, Funding acquisition, Investigation, Methodology, Writing – original draft, Writing – review & editing. TS: Conceptualization, Data curation, Formal analysis, Funding acquisition, Investigation, Methodology, Supervision, Writing – review & editing PW: Data curation, Formal analysis, Investigation, Methodology, Software, Writing – review & editing. NF: Data curation, Formal analysis, Investigation, Methodology, Software, Visualization, Writing – review & editing. MP: Data curation, Formal analysis, Investigation, Methodology, Writing – review & editing. ZM: Data curation, Investigation, Methodology, Writing – review & editing. SR: Conceptualization, Data curation, Formal analysis, Funding acquisition, Investigation, Methodology, Resources, Supervision, Writing – review & editing. JC: Conceptualization, Data curation, Formal analysis, Investigation, Methodology, Supervision, Visualization, Writing – original draft, Writing – review & editing. GL: Conceptualization, Data curation, Formal analysis, Funding acquisition, Investigation, Methodology, Project administration, Resources, Supervision, Writing – original draft, Writing – review & editing.

## Supplementary material

Supplementary Data Sheet 1 RNA-seq RPKM values, gene annotations, DEGs, raw counts, and SRA accessions.

Supplementary Data Sheet 2 GO term enrichment analysis.

Supplementary Figure S1 Dimorphic diaspore responses of Aethionema arabicum to temperature.

Supplementary Figure S2 Effect of a range of constant temperatures on the growth of Aethionema arabicum M+ and M- seedlings.

Supplementary Figure S3 Effect of water potential (osmotica) on the growth of Aethionema arabicum M+ and M- seedlings.

Supplementary Figure S4 Enrichment scores for selected GO terms significantly enriched in at least one selected DEG list.

Supplementary Figure S5 Comparative spatiotemporal analysis of transcript abundance patterns of selected Aethionema arabicum ethylene and ABA-related DEGs.

Supplementary Figure S6 Comparative spatiotemporal analysis of transcript abundance patterns of selected Aethionema arabicum GA and auxin-related DEGs.

Supplementary Figure S7 Comparative spatiotemporal analysis of transcript abundance patterns of Aethionema arabicum chlorophyll-related and transporter DEGs.

Supplementary Figure S8 Comparative spatiotemporal analysis of transcript abundance patterns of Aethionema arabicum transcription factor (TF) DEGs.

Supplementary Figure S9 Comparative spatiotemporal analysis of transcript abundance patterns of Aethionema arabicum germin, LEA and HSP DEGs.

Supplementary Figure S10 Comparative spatiotemporal analysis of transcript abundance patterns of Aethionema arabicum cell wall remodelling protein (CWRP) DEGs.

Supplementary Table S1 Number of Aethionema arabicum DEGs identified.

Supplementary Table S2 Aethionema arabicum gene names and IDs.
