# Supplementary material for: Aethionema arabicum dimorphic seed trait resetting during transition to seedlings
Source: Front Plant Sci. 2024 Mar 8;15:1358312. doi: 10.3389/fpls.2024.1358312 (PMC10957558; doi:10.3389/fpls.2024.1358312)
Supplement: Supplementary Data Sheet 1 — RNA-seq RPKM values, gene annotations, DEGs, raw counts, and SRA accessions. [file DataSheet_1.zip › Supplementary Figures and Tables.pdf]

Supplementary Material

Aethionema arabicum dimorphic seed trait resetting during transition to seedlings

Waheed Arshad, Tina Steinbrecher, Per K.I. Wilhelmsson, Noe Fernandez-Pozo, Marta Pérez, Zsuzsanna, Stefan A. Rensing, Jake O. Chandler, Gerhard Leubner-Metzger

Frontiers in Plant Science 2024

Research Topic "Underlying Mechanisms Transitioning Seeds to Seedlings"

Supplementary Figure S1 | Dimorphic diaspore responses of *Aethionema arabicum* to temperature.

Supplementary Figure S2 | Effect of a range of constant temperatures on the growth of *Aethionema arabicum* M^+ and M^- seedlings.

Supplementary Figure S3 | Effect of water potential (osmotica) on the growth of *Aethionema arabicum* M^+ and M^- seedlings.

Supplementary Figure S4 | Enrichment scores for selected GO terms significantly enriched in at least one selected DEG list.

Supplementary Figure S5 | Comparative spatiotemporal analysis of transcript abundance patterns of selected *Aethionema arabicum* ethylene and ABA-related DEGs.

Supplementary Figure S6 | Comparative spatiotemporal analysis of transcript abundance patterns of selected *Aethionema arabicum* GA and auxin-related DEGs.

Supplementary Figure S7 | Comparative spatiotemporal analysis of transcript abundance patterns of *Aethionema arabicum* chlorophyll-related and transporter DEGs.

Supplementary Figure S8 | Comparative spatiotemporal analysis of transcript abundance patterns of *Aethionema arabicum* transcription factor (TF) DEGs.

Supplementary Figure S9 | Comparative spatiotemporal analysis of transcript abundance patterns of *Aethionema arabicum* germin, LEA and HSP DEGs.

Supplementary Figure S10 | Comparative spatiotemporal analysis of transcript abundance patterns of *Aethionema arabicum* cell wall remodelling protein (CWRP) DEGs.

Supplementary Table S1 | Number of *Aethionema arabicum* DEGs identified.

Supplementary Table S2 | *Aethionema arabicum* gene names and IDs.

Copyright © 2024 Arshad, Steinbrecher, Wilhelmsson, Fernandez-Pozo, Pérez, Mérai, Rensing, Chandler, Leubner-Metzger. This is an open-access article distributed under the terms of the Creative Commons Attribution License (CC BY). The use, distribution or reproduction in other forums is permitted, provided the original author(s) and the copyright owner(s) are credited and that the original publication in this journal is cited, in accordance with accepted academic practice. No use, distribution or reproduction is permitted which does not comply with these terms.

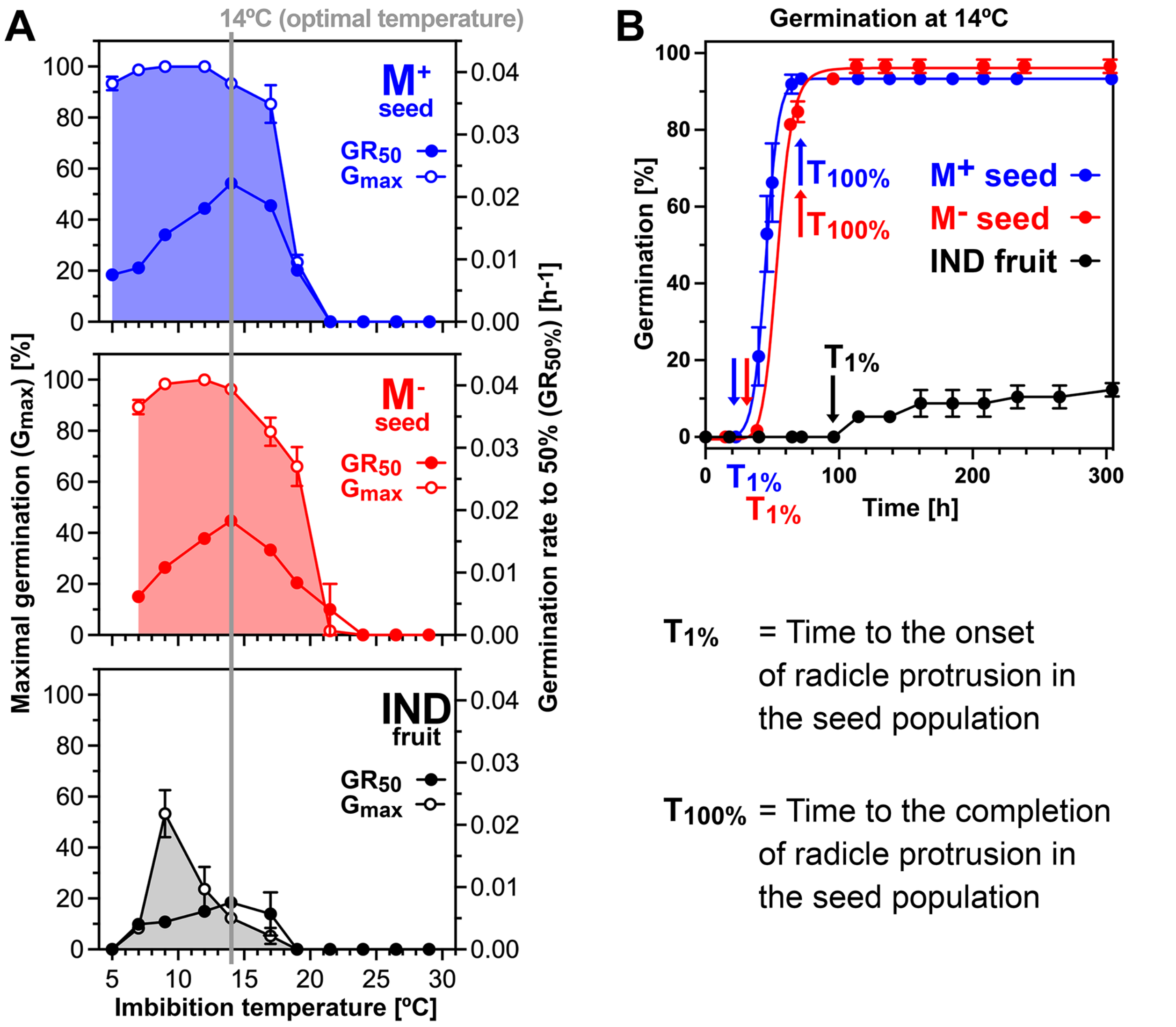

Supplementary Figure S1 | Dimorphic diaspore responses of *Aethionema arabicum* to ambient temperatures. (A) The effect of imbibition temperatures (constant white light 170 µmol • m^{-2} • s^{-1}) on the maximal germination percentages (G_{max}) and the speed of germination expressed as germination rate ($GR_{50\%} = 1/T_{50\%}$, a measure for the speed of germination with $T_{50\%}$ being the time required to reach 50% G_{max}) of the dimorphic diaspores (M^+ seeds, IND fruits), and for comparison of bare M^- seeds (extracted from IND fruits by pericarp removal). **(B)** 14ºC was identified as optimal temperature with the highest germination rate of freshly harvested mature diaspores. Mean ± SEM values of 3 replicate plates each with ca. 30 seeds.

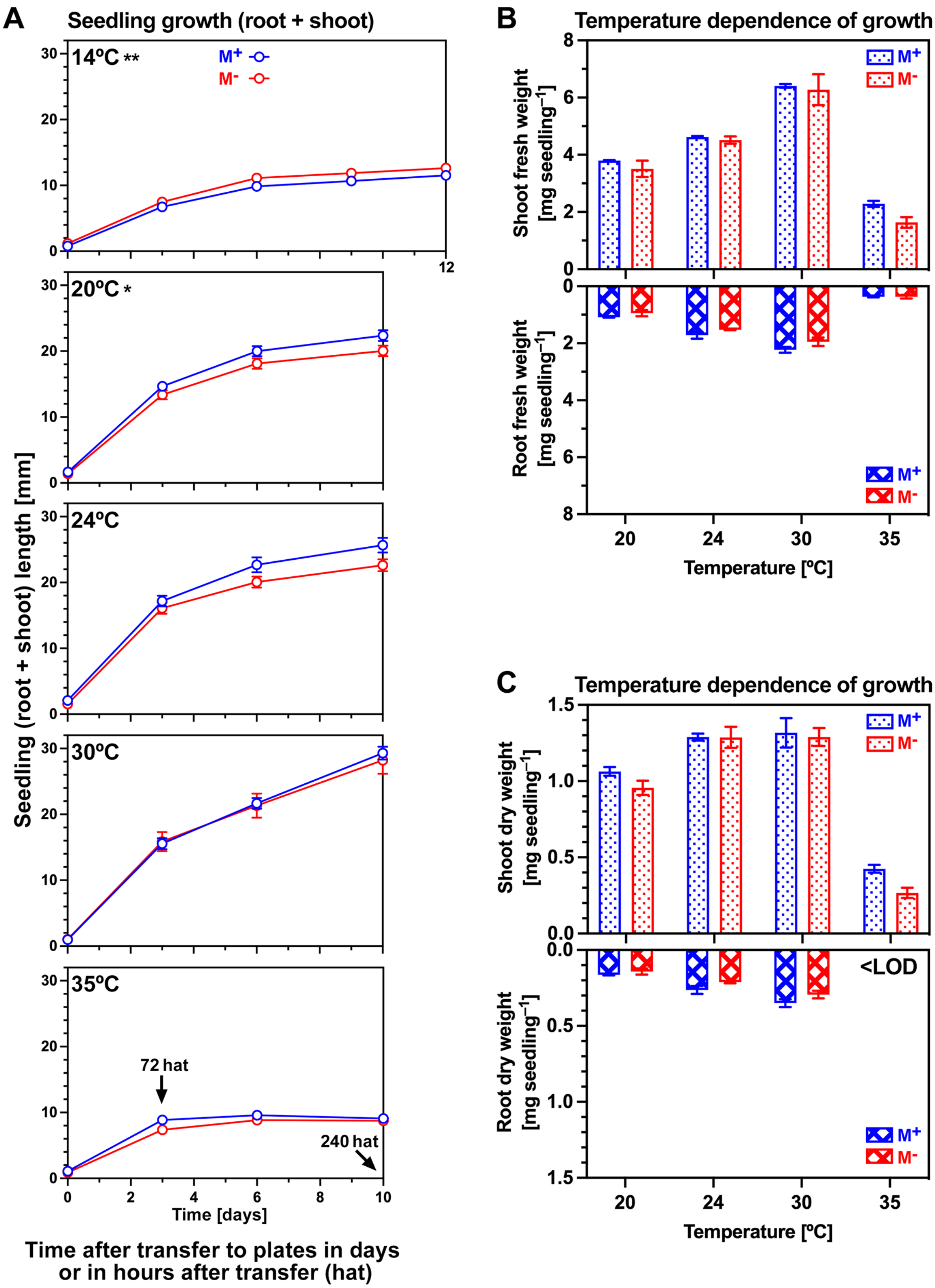

Supplementary Figure S2 | The effect of a range of constant temperatures on the growth of *Aethionema arabicum* M$^+$ and M$^-$ seedlings. (A) M$^+$ and M$^-$ seedlings derived from M$^+$ and M$^-$ seeds were grown on vertical agar plates in continuous white light (170 µmol • m^{-2} • s^{-1}) at constant

temperatures as indicated. Seedling growth assays were conducted with germinated seeds (surface-sterilised seeds germinated in darkness at 9ºC) which were selected for transfer to agar plates containing media based on 1 mm protrusion of the radicle (0 hat, hours after transfer). The mean ± SEM (N = 3 plates, each with 7 seedlings) of combined root and shoot lengths were calculated over an experimental period of 10 days. Although the total seedling length differed between morphs at 14ºC (P = 0.003, **) and 20ºC (P = 0.025, *), length under higher temperatures of 24ºC (P = 0.076), 30ºC (P = 0.834), and 35ºC (P = 0.123) did not elicit such a morph-specific response. For 14ºC seedling length at day 10 (presented in Figure 2), the values were derived from the presented growth curves as 10.96 ± 0.41 for M^+ and 12.13 ± 0.20 for M^- seedlings. M^+ and M^- seedling shoot and root fresh weights **(B)** and dry weights **(C)** after 10 days (240 hat) of growth under temperatures representative of thermal stress. Error bars = ± 1 SEM. N = 3, each with 7 seedlings. LOD = limit of detection. There were no morph-specific differences in fresh (P = 0.094) and dry (P = 0.068) shoot masses across growth temperatures. In root tissues, ANOVA revealed that seed morph accounted for 1.36% of the total variance with fresh weight (P = 0.026), and 1.71% of the total variance with dry weight (P = 0.019). Together with seedling lengths, growth rates (Figure 2), and fresh weights, the most optimal seedling growth conditions were thus considered as 30ºC.

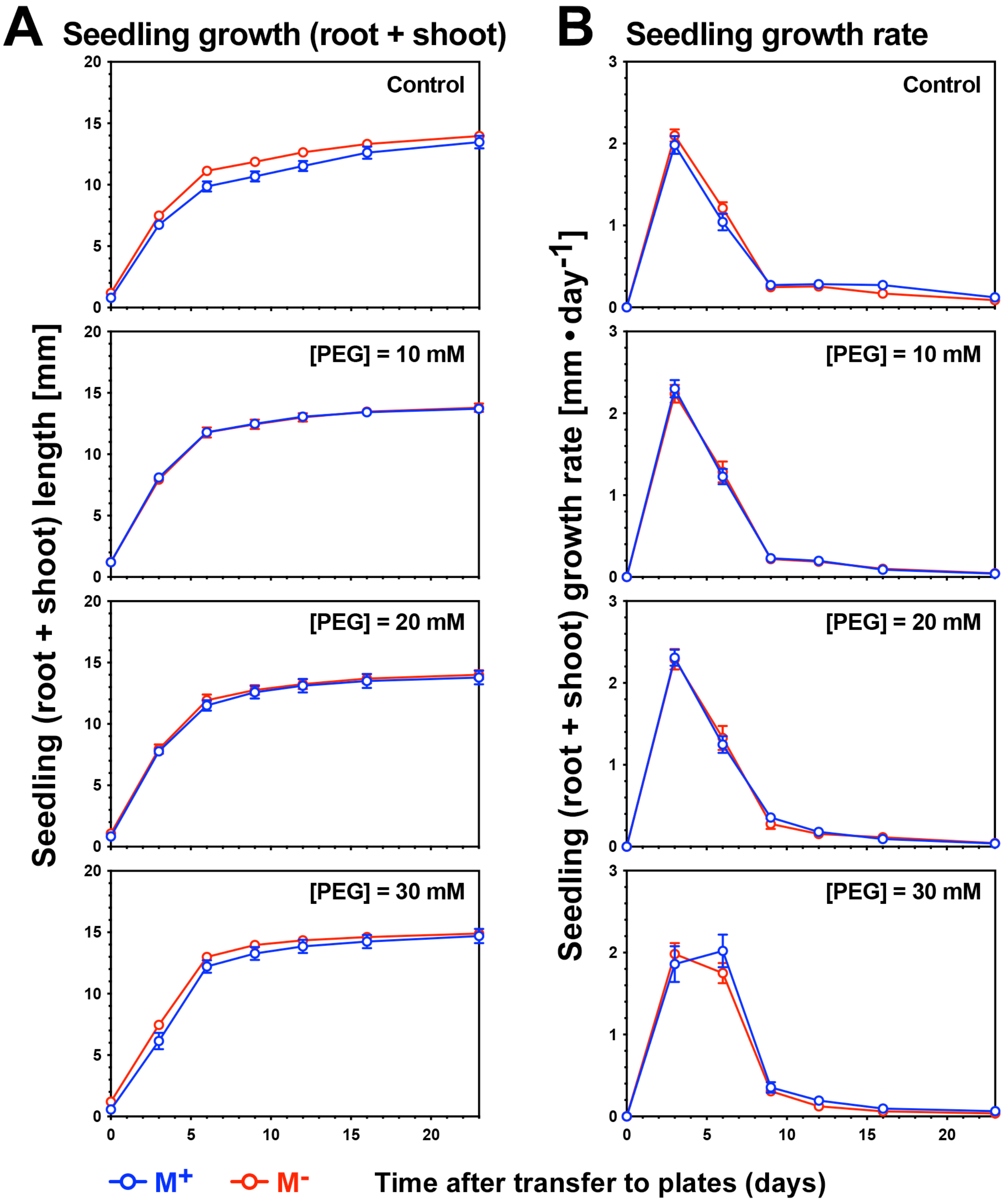

Figure S3 continued next page…

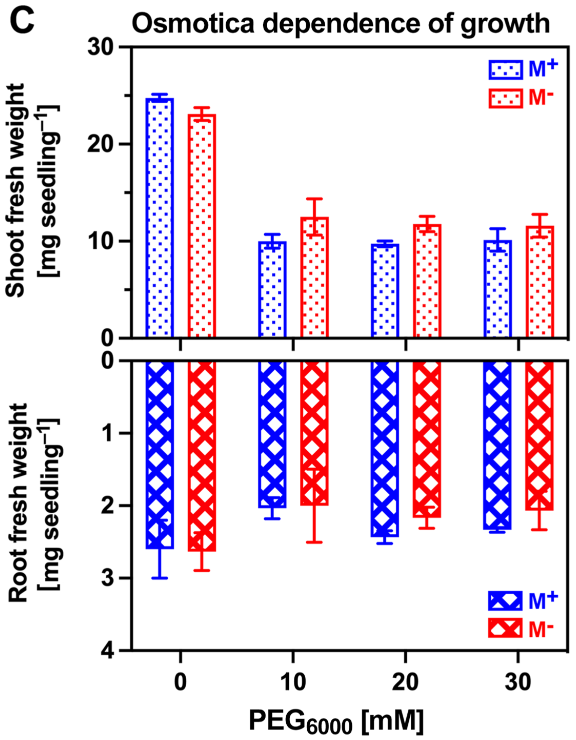

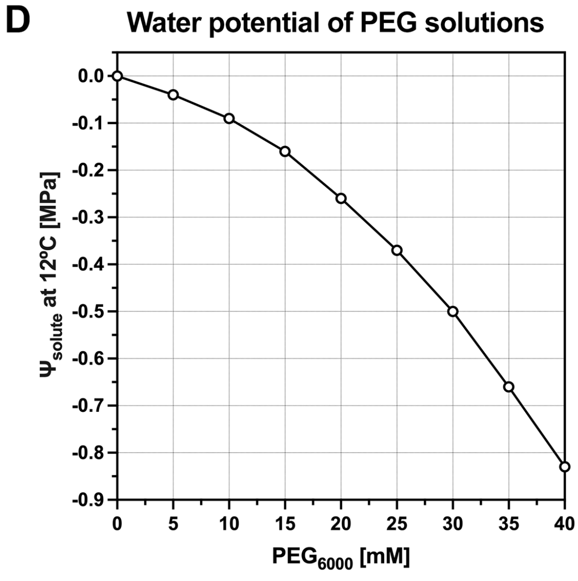

Supplementary Figure S3 | The effect of water potential (osmotica) on the growth of *Aethionema arabicum* M^+ and M^- seedlings. **(A)** Seedlings growth responses on vertical agar plates at 14ºC in continuous white light (170 µmol • m^{-2} • s^{-1}) to increasing concentrations (0, 10, 20 and 30 mM) of polyethylene glycol (PEG). The means of combined root and shoot lengths were calculated over an experimental period of 23 days. At 552 hat, seedling morphs did not differ in their total length and growth rates under three different concentrations of PEG. ANOVA revealed there were no significant morph-specific differences in seedling size at 10 mM (P = 0.838), 20 mM (P = 0.818), or 30 mM (P = 0.157) PEG. Error bars = ± 1 SEM. N = 3, each with 7 seedlings. **(B)** Corresponding seedlings growth rates. ANOVA of the growth rates revealed no differences between M+ and M– seedlings across water potentials. Similar patterns of growth were observed at control (P = 0.548), 10 mM (P = 0.994), 20 mM (P = 0.698), and 30 mM (P = 0.248) conditions. **(C)** Mean fresh weights of separated root and shoot tissue from 31 day-old seedlings grown at 14ºC under different osmotic stress conditions using PEG. ANOVA revealed neither shoot fresh weights (P = 0.145) nor root fresh weights (P = 0.502) had a significant morph-specific difference at each PEG concentration. Error bars = ± 1 SEM. N = 3, each with 7 seedlings. **(D)** For seedling growth assays during osmotic stress, water potentials were lowered by using high-molecular weight polyethylene glycol (PEG_{6000}) and an overlay method as described (van der Weele et al., 2000; Verslues and Bray, 2004). Osmotic potentials (ψ_{solute}) of PEG_{6000} are shown, based on the formula derived by Michel and Kaufmann (1973), as determined under the optimal germination temperature (12ºC) for M^+ and M^- seed diaspores. The osmotic potential exhibited a curvilinear relationship with concentration, serving as a proxy for the extent of osmotic stress during seedling growth.

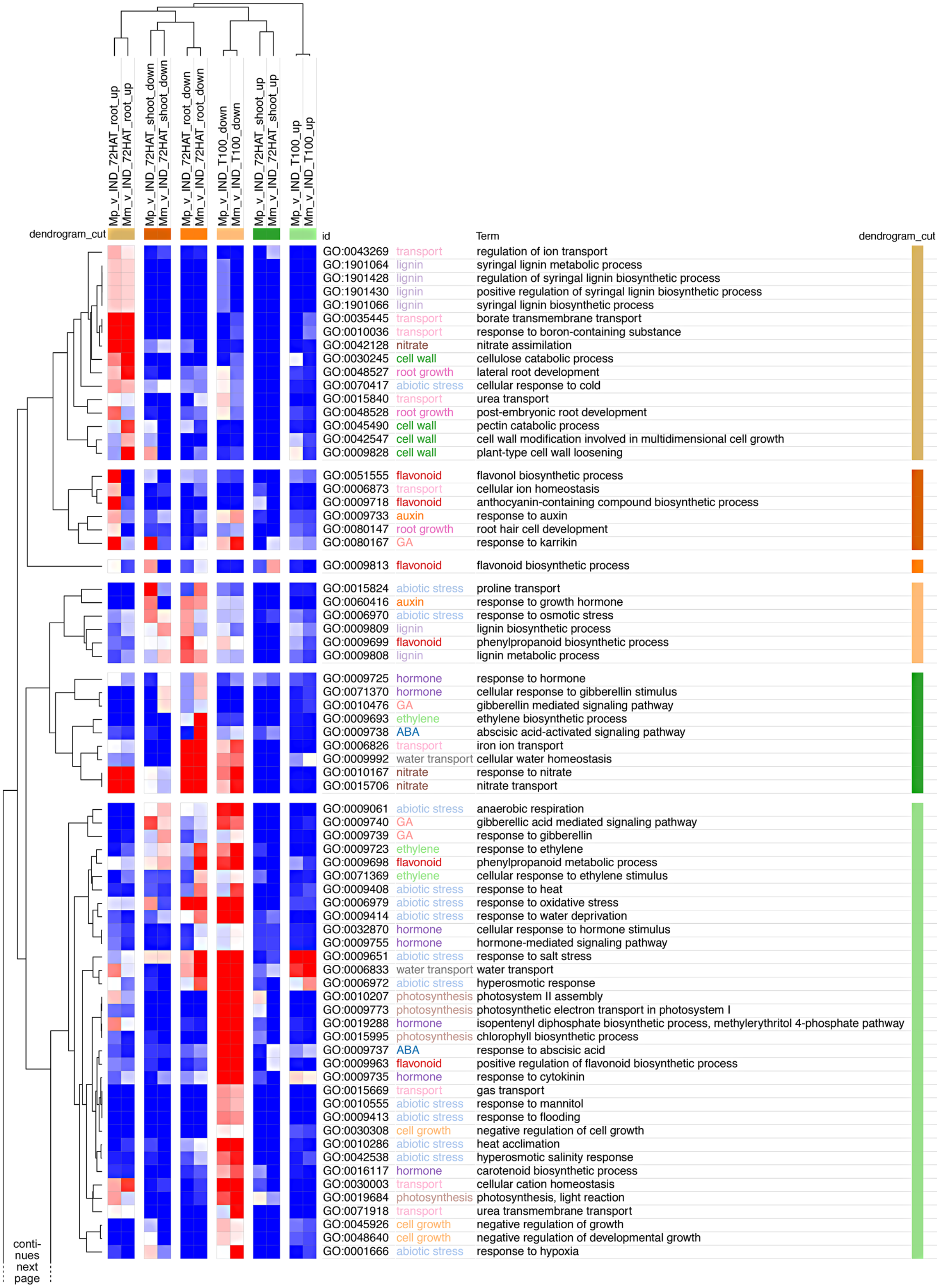
Mp_v_IND_72HAT_root_up
Mm_v_IND_72HAT_root_up
Mp_v_IND_72HAT_shoot_down
Mm_v_IND_72HAT_shoot_down
Mp_v_IND_72HAT_root_down
Mm_v_IND_72HAT_root_down
Mp_v_IND_T100_down
Mm_v_IND_T100_down
Mp_v_IND_72HAT_shoot_up
Mm_v_IND_72HAT_shoot_up
Mp_v_IND_T100_up
Mm_v_IND_T100_up
dendrogram_cut
id
Term
dendrogram_cut
GO:0043269 transport regulation of ion transport
GO:1901064 lignin syringal lignin metabolic process
GO:1901428 lignin regulation of syringal lignin biosynthetic process
GO:1901430 lignin positive regulation of syringal lignin biosynthetic process
GO:1901066 lignin syringal lignin biosynthetic process
GO:0035445 transport borate transmembrane transport
GO:0010036 transport response to boron-containing substance
GO:0042128 nitrate nitrate assimilation
GO:0030245 cell wall cellulose catabolic process
GO:0048527 root growth lateral root development
GO:0070417 abiotic stress cellular response to cold
GO:0015840 transport urea transport
GO:0048528 root growth post-embryonic root development
GO:0045490 cell wall pectin catabolic process
GO:0042547 cell wall cell wall modification involved in multidimensional cell growth
GO:0009828 cell wall plant-type cell wall loosening
GO:0051555 flavonoid flavonol biosynthetic process
GO:0006873 transport cellular ion homeostasis
GO:0009718 flavonoid anthocyanin-containing compound biosynthetic process
GO:0009733 auxin response to auxin
GO:0080147 root growth root hair cell development
GO:0080167 GA response to karrikin
GO:0009813 flavonoid flavonoid biosynthetic process
GO:0015824 abiotic stress proline transport
GO:0060416 auxin response to growth hormone
GO:0006970 abiotic stress response to osmotic stress
GO:0009809 lignin lignin biosynthetic process
GO:0009699 flavonoid phenylpropanoid biosynthetic process
GO:0009808 lignin lignin metabolic process
GO:0009725 hormone response to hormone
GO:0071370 hormone cellular response to gibberellin stimulus
GO:0010476 GA gibberellin mediated signaling pathway
GO:0009693 ethylene ethylene biosynthetic process
GO:0009738 ABA abscisic acid-activated signaling pathway
GO:0006826 transport iron ion transport
GO:0009992 water transport cellular water homeostasis
GO:0010167 nitrate response to nitrate
GO:0015706 nitrate nitrate transport
GO:0009061 abiotic stress anaerobic respiration
GO:0009740 GA gibberellic acid mediated signaling pathway
GO:0009739 GA response to gibberellin
GO:0009723 ethylene response to ethylene
GO:0009698 flavonoid phenylpropanoid metabolic process
GO:0071369 ethylene cellular response to ethylene stimulus
GO:0009408 abiotic stress response to heat
GO:0006979 abiotic stress response to oxidative stress
GO:0009414 abiotic stress response to water deprivation
GO:0032870 hormone cellular response to hormone stimulus
GO:0009755 hormone hormone-mediated signaling pathway
GO:0009651 abiotic stress response to salt stress
GO:0006833 water transport water transport
GO:0006972 abiotic stress hyperosmotic response
GO:0010207 photosynthesis photosystem II assembly
GO:0009773 photosynthesis photosynthetic electron transport in photosystem I
GO:0019288 hormone isopentenyl diphosphate biosynthetic process, methylerythritol 4-phosphate pathway
GO:0015995 photosynthesis chlorophyll biosynthetic process
GO:0009737 ABA response to abscisic acid
GO:0009963 flavonoid positive regulation of flavonoid biosynthetic process
GO:0009735 hormone response to cytokinin
GO:0015669 transport gas transport
GO:0010555 abiotic stress response to mannitol
GO:0009413 abiotic stress response to flooding
GO:0030308 cell growth negative regulation of cell growth
GO:0010286 abiotic stress heat acclimation
GO:0042538 abiotic stress hyperosmotic salinity response
GO:0016117 hormone carotenoid biosynthetic process
GO:0030003 transport cellular cation homeostasis
GO:0019684 photosynthesis photosynthesis, light reaction
GO:0071918 transport urea transmembrane transport
GO:0045926 cell growth negative regulation of growth
GO:0048640 cell growth negative regulation of developmental growth
GO:0001666 abiotic stress response to hypoxia
conti-
nues
next
page

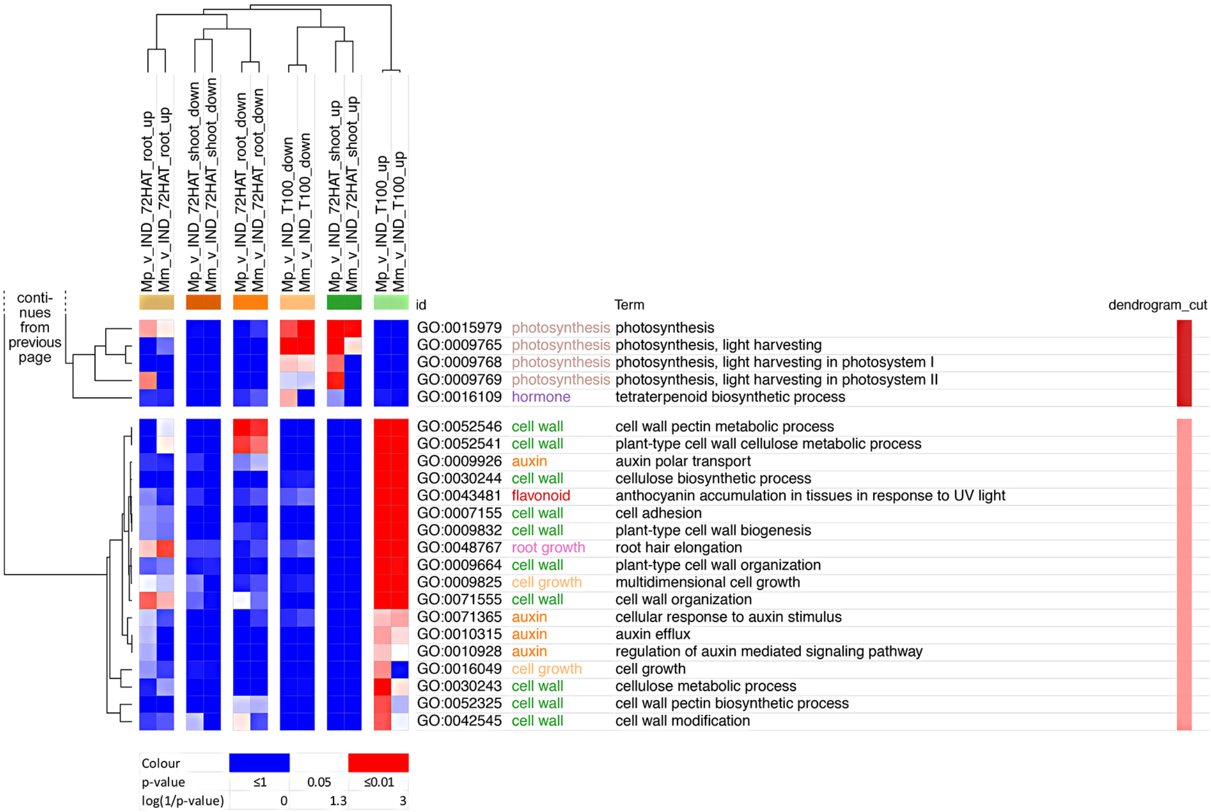

Supplementary Figure S4 | Enrichment scores for selected GO terms significantly enriched in at least one selected DEG list. GO terms were selected, assigned categories, and enrichment scores (log(1/p-value)) were clustered hierarchically by 1-pearson correlation using Morpheus (https://software.broadinstitute.org/morpheus/). Blue indicates non-significantly enriched values ($p > 0.05$), with white representing the significant cutoff ($p = 0.05$) and red indicating where GO terms are significantly enriched in the DEG lists ($p < 0.05$), saturated at $p = 0.001$. Full GO term enrichment p-values for all DEG lists can be found in Supplementary Dataset 2. Selected GO terms are represented in Figure 8. Mp = M^+ seed, Mm = M^- seed, IND = IND fruit; GA = gibberellin.

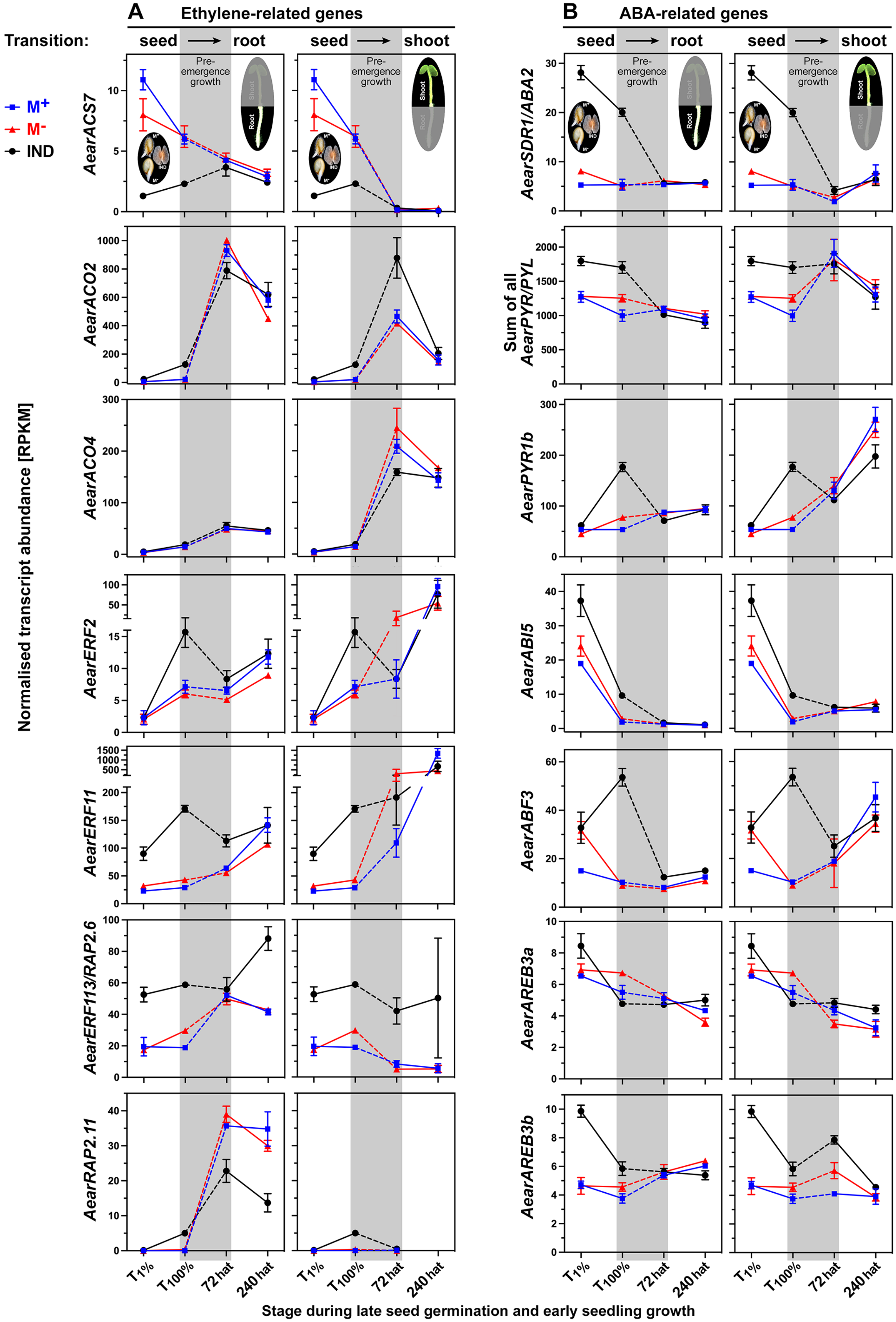
A Ethylene-related genes
B ABA-related genes
Transition:
seed → root
seed → shoot
Pre-emergence growth
M+
M-
IND
AearACS7
AearACO2
AearACO4
AearERF2
AearERF11
AearERF113/RAP2.6
AearRAP2.11
AearSDR1/ABA2
Sum of all AearPYR/PYL
AearPYR1b
AearABI5
AearABF3
AearAREB3a
AearAREB3b
Normalised transcript abundance [RPKM]
T1%
T100%
72 hat
240 hat
Stage during late seed germination and early seedling growth

S5

Supplementary Figure S5 | Comparative spatiotemporal analysis of transcript abundance patterns of selected *Aethionema arabicum* DEGs. (A) Ethylene-related genes. **(B)** Abscisic acid (ABA)-related genes. **(A,B)** M^+ seeds, M^- seeds, and IND fruits were imbibed in dH_2O under darkness at 9ºC, and sampled at harvested at $T_{1\%}$ and $T_{100\%}$. Diaspores which had completed germination (1 mm radicle protrusion) were transferred to plates containing 1⁄10 MS and 1% (w/v) agar, and grown vertically under constant light (170 µmol m^{-2} s^{-1}) at 30ºC. RNA-seq mean ± SEM values of 3 biological replicates are presented, each replicate consisted of 90 seeds or tissue (root or shoot) from 12 seedlings. The pre-emergence growth phase leading from germinated diaspores ($T_{100\%}$) to seedlings at 72 and 240 hours after transfer (hat) is shaded grey; seed-seedling transition RNA-seq values for roots (*left panels*) and shoots (*right panels*) are presented. See main text for details about specific genes and Supplementary Table S2 for gene names and identifier.

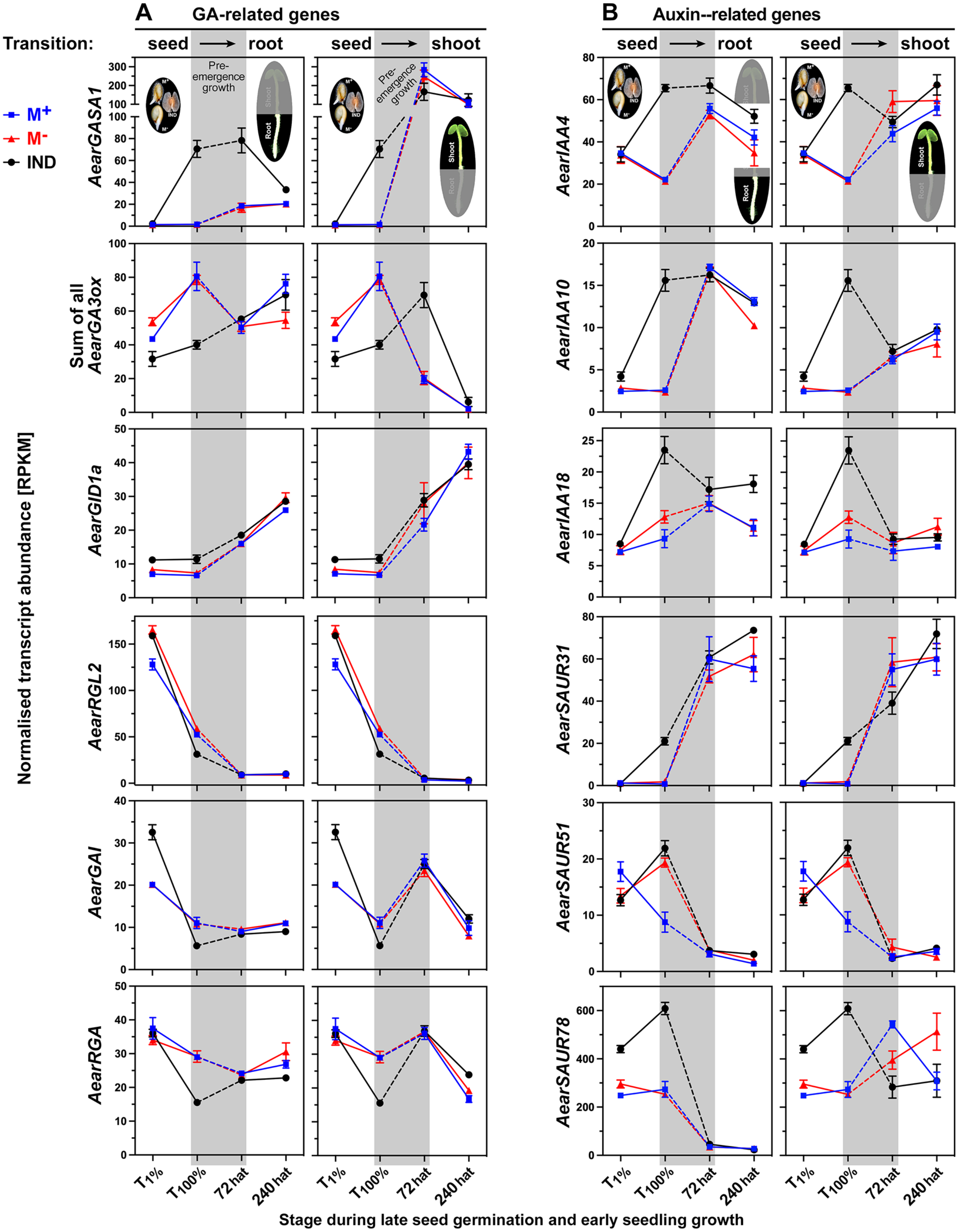

Supplementary Figure S6 | Comparative spatiotemporal analysis of transcript abundance patterns of selected *Aethionema arabicum* DEGs. (A) Gibberellin (GA)-related genes. **(B)** Auxin-related genes. For details see legend Supplementary Figure S5 and Supplementary Table S2 for genes.

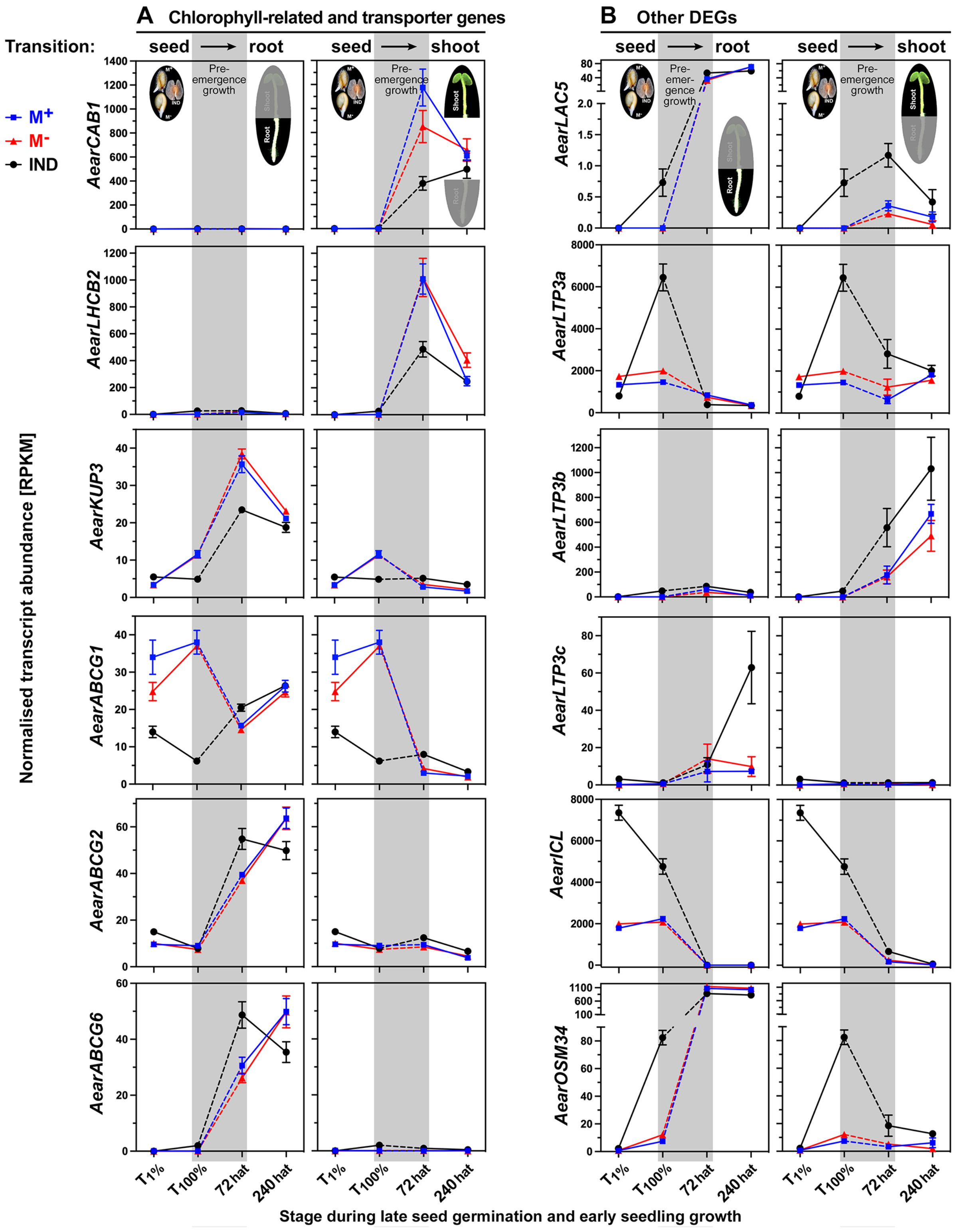

Supplementary Figure S7 | Comparative spatiotemporal analysis of transcript abundance patterns of selected *Aethionema arabicum* DEGs. (A) Chlorophyll-related and transporter genes. **(B)** Other DEGs. For details see legend Supplementary Figure S5 and Supplementary Table S2 for genes. Note that ATP-binding cassette (ABC) transporters ABCG1, ABCG2 and ABCG6 are known for their auxin and ABA controlled involvement in suberin layer formation in during seed and pollen development (Yadav et al., 2014; Liu et al., 2020; Choi et al., 2023). While *AearABCG1* is a DEG

during *Ae. arabicum* germination, *AearABCG2* and *AearABCG6* are DEGs during pre-emergence seedling growth with higher transcript abundances in 72 hat roots derived from IND-fruits. Seed-coat protective neolignans are produced by the laccase AtLAC5 in *A. thaliana* seeds (Yonekura-Sakakibara et al., 2021). During *Ae. arabicum* pre-emergence seedling growth, *AearLAC5* transcript abundances were higher during late germination and in 72 hat shoots derived from IND fruits. Lipid Transfer Protein 3 (AtLPT3) is required for lipid mobilisation during seed germination and seedling growth, and also for mediating freezing and drought stress responses in *A. thaliana* (Guo et al., 2013; Pagnussat et al., 2015) and from as ABA regulated process during the seed-to-seedling transition of pea where they are proposed to be involved in desiccation tolerance loss during seed-to-seedling transition (Smolikova et al., 2022). Differential expression and higher transcript abundances in *Ae. arabicum* seedlings derived from IND fruits was observed for three *LPT3* homologs. Known seed-to-seedling phase transition genes in *A. thaliana* include isocitrate lyase (ICL) which is a marker of seedling vigour (de los Reyes et al., 2003) and *AearOSM34* involved in responses to salinity (Park and Kim, 2021).

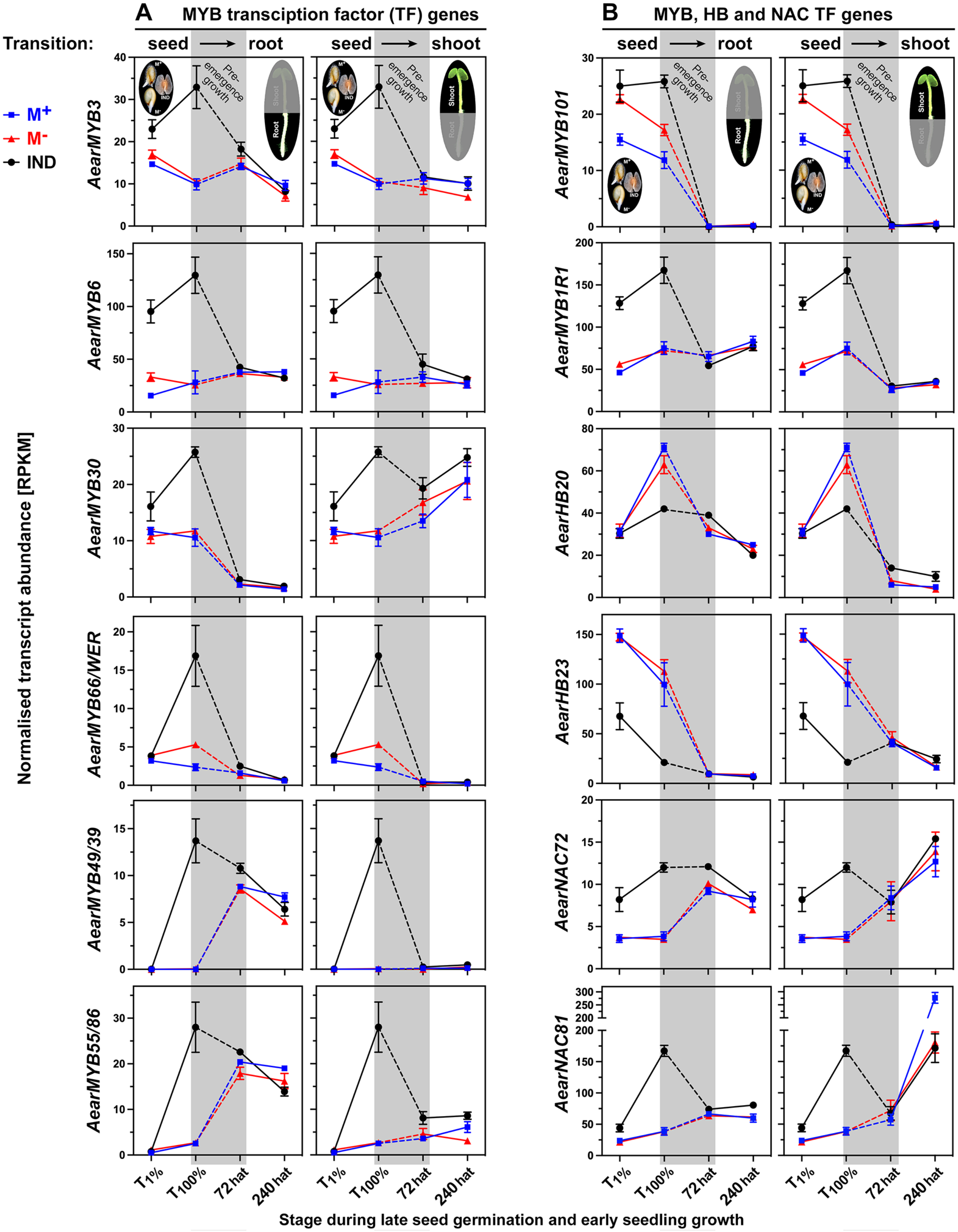

Figure S8 continued next page…

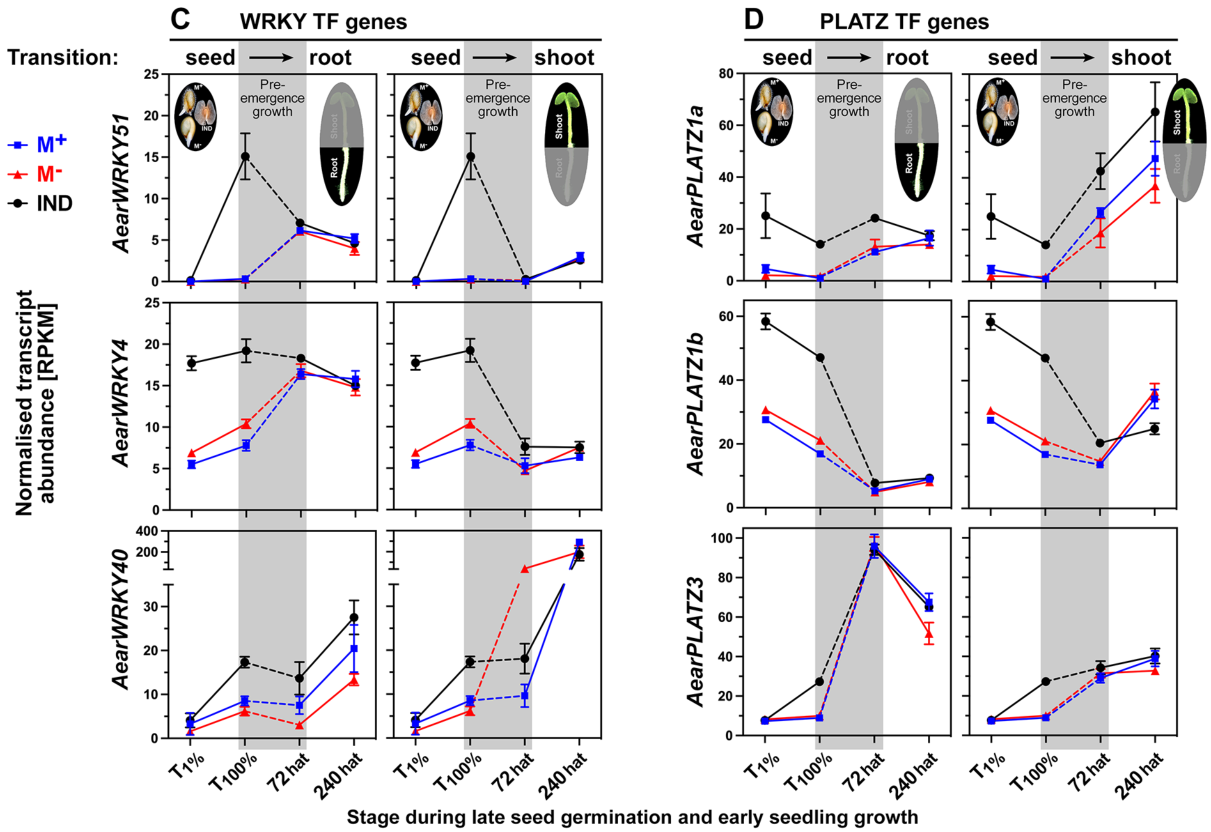

Supplementary Figure S8 | Comparative spatiotemporal analysis of transcript abundance patterns of *Aethionema arabicum* transcription factor (TF) DEGs. (A,B,C,D) MYB, HB, NAC, WRKY and PLATZ TF genes. For details see legend Supplementary Figure S5, the main text for specific genes and Supplementary Table S2 for gene names and identifier. Note that the homeobox TFs HB13, HB20 and HB23 constitute node-regulators within the co-expression network controlling seed-to-seedling phase transition in *A. thaliana* (Silva et al., 2016). HB20 and HB25 also control seed ABA sensitivity, dormancy, longevity and embryo growth (Barrero et al., 2010; Renard et al., 2021). In agreement with our earlier work (Chandler et al., 2023) and their role as seed-seedling transition markers, *AearHB20* and *AearHB25* were DEGs between the IND fruit and M^+ seed diaspores during late germination and resetting occurred during the seed-to-seedling phase transition. The plant-specific AT-rich sequence zinc-binding protein (PLATZ) TFs are known in *A. thaliana* and tomato for their roles in seedling temperature responses, and *AtPLATZ1* (synonym *AtAIN1*, ABA-induced expression 1) is involved in the ABA-mediated inhibition of root elongation by modulating ROS homeostasis (Dong et al., 2021; Wai et al., 2022). In *Ae. arabicum AearPLATZ1a*, *AearPLATZ1b*, and *AearPLATZ3* were identified as DEGs during dimorphic diaspore germination. While *AearPLATZ1b* and *AearPLATZ3* expression exhibited resetting during post-germination pre-emergence growth, *AearPLATZ1a* transcript abundances remained higher also in 72 hat seedling roots and shoots derived from germinated IND fruits.

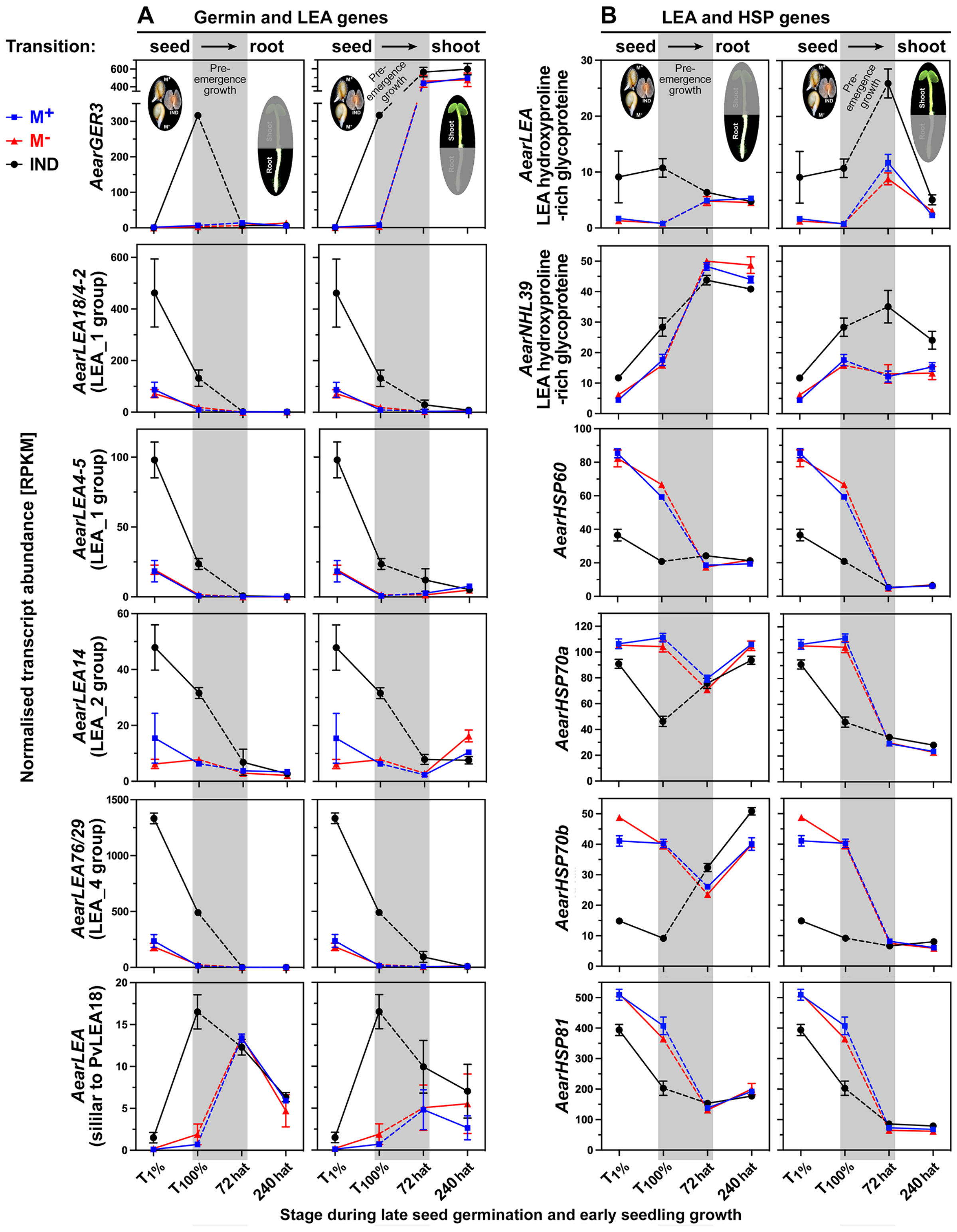

Supplementary Figure S9 | Comparative spatiotemporal analysis of transcript abundance patterns of selected *Aethionema arabicum* DEGs. (A) Germin (GER) and Late Embryogenesis Abundant (LEA) genes. **(B)** LEA and Heat Shock Protein (HSP) genes. For details see legend Supplementary Figure S5, the main text for specific genes and Supplementary Table S2 for gene names and identifier. Note that LEA group 1, 2 and 4 gene trancript abundances declined during imbibition and reached low levels in 72 hat seedlings. The transcript abundances of two LEA hydroxyproline-rich

glycoproteins remained high in shoots of 72 hat seedlings derived from IND fuits when compared to seedlings derived from M^+ and M^- seeds. In contrast to group 1, 2 and 4 LEA genes, genes encoding Heat Shock Proteins (HSPs) were lower expressed in imbibed IND fruits as compared to M^+ and M^- seeds. As for group 1, 2 and 4 LEA genes, the HSP transcript abundances declined and resetting occurred during pre-emergence growth. Many of these genes are ABA regulated and involved in desiccation tolerance loss durimg seed-to-seedling transition (Hundertmark and Hincha, 2008; Zinsmeister et al., 2020; Smolikova et al., 2022).

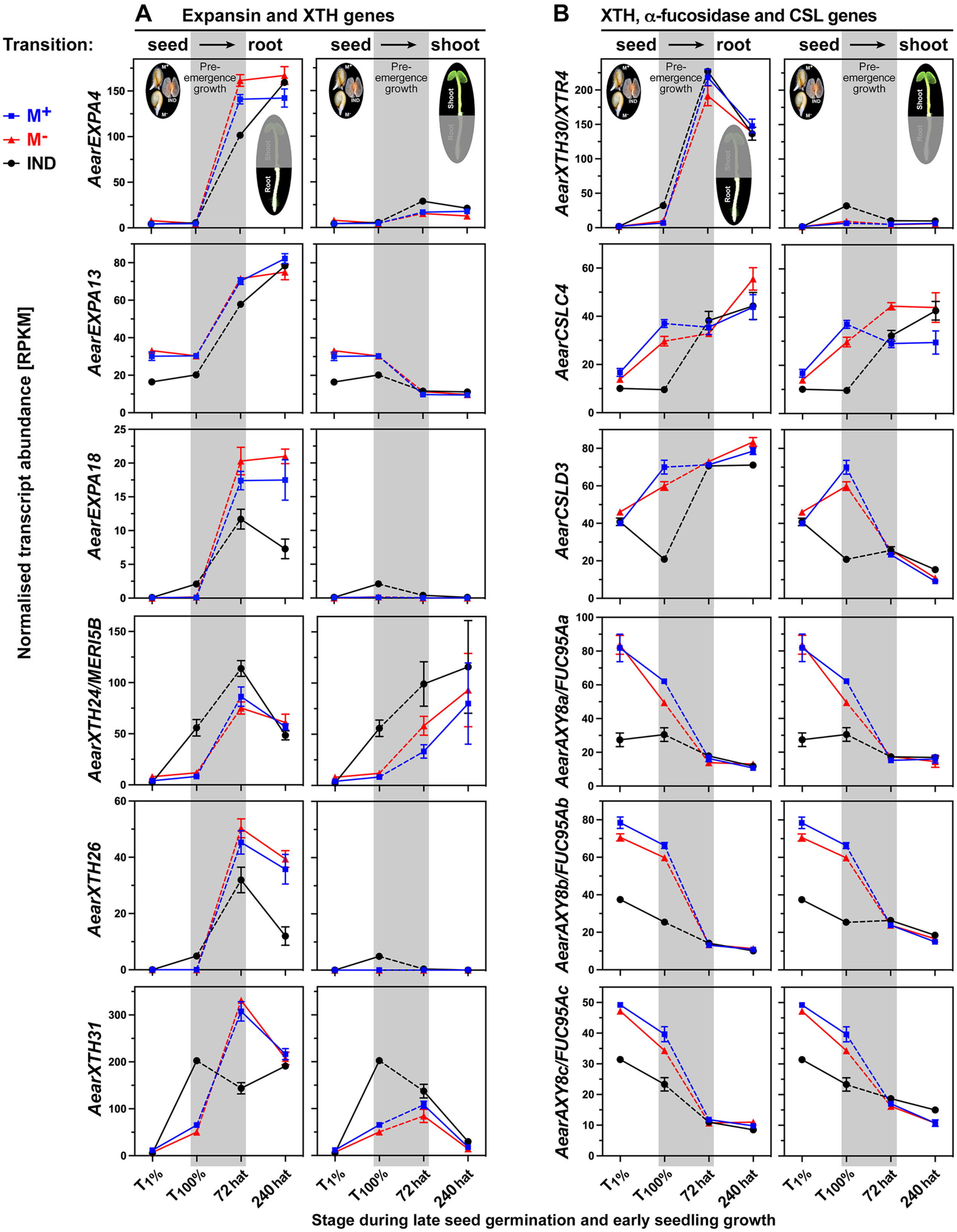

Figure S10 continued next page…

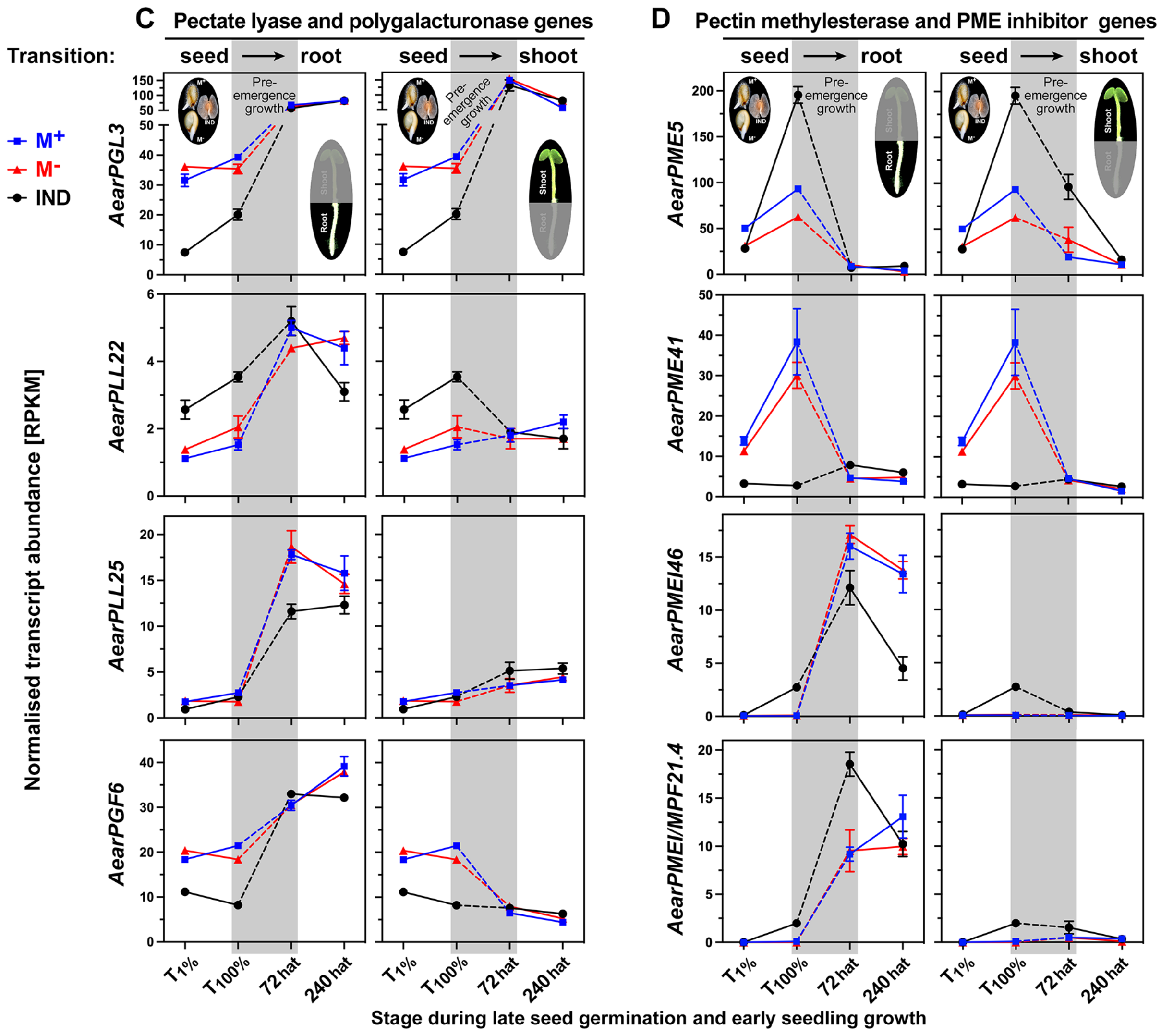

Supplementary Figure S10 | Comparative spatiotemporal analysis of transcript abundance patterns of selected *Aethionema arabicum* DEGs. (A) Expansins and xyloglucan endotrans-glucosylase/hydrolase (XTH) genes. **(B)** XTH, α-fucosidase and glucan synthase (CSLC) genes. **(C)** Pectate lyase (PLL) and polygalacturonase (PG) genes. **(D)** Pectin methylesterase (PME) PME inhibitor (PMEI) genes. Note that α-fucosidases have α-1,2-fucose in xyloglucan sidechains as targets, and glucan synthase (CSLC) the β-1,4-glucose backbone of xyloglucan to control germination and seedling growth (Steinbrecher and Leubner-Metzger, 2017; Moneo-Sanchez et al., 2019; Steinbrecher and Leubner-Metzger, 2022). In *Ae. arabicum* resetting during pre-emergence growth was observed for *AearCSLC4* and *AearFUC*. Pectins are complex cell wall matrix polysaccharides characterized by α-1,4-linked galacturonic acid and a dymamic degree of methylesterification (Cao, 2012; Daher and Braybrook, 2015; Scheler et al., 2015). Several PLL, PG, PME and PMEI genes are DEGs in *Ae. arabicum* dimorphic diaspores and most of them exhibited resetting during pre-emergence growth. For details see legend Supplementary Figure S5, the main text for specific genes and Supplementary Table S2 for gene names and identifier.

Supplementary Table S1 | Numbers of identified *Aethionema arabicum* DEGs based on a p-value cut off set to 0.001 as previously described by Wilhelmsson et al. (2019). Shown are DEGs up- (↑) and down- (↓) regulated based on pairwise comparisons of M^+ seed vs. IND fruit (M^+/IND), $M^–$ seed vs. IND fruit (M^+/IND) and M^+ seed vs. M^- seed (M^+/M^-).

**Diaspore comparison M^+ versus IND**			**Number DEGs based on p-value**		
			Total	UP in M^+	DOWN in M^+
M^+ versus IND	Seed	$T_{1\%}$	2041	867	1174
M^+ versus IND	Seed	$T_{100\%}$	2682	932	1750
M^+ versus IND	Root	72 hat	277	126	151
M^+ versus IND	Shoot	72 hat	164	36	128
M^+ versus IND	Root	240 hat	60	41	19
M^+ versus IND	Shoot	240 hat	10	5	5

**Pericarp effect M^- versus IND**			**Number DEGs based on p-value**		
			Total	UP in M^-	DOWN in M^-
M^- versus IND	Seed	$T_{1\%}$	1938	865	1073
M^- versus IND	Seed	$T_{100\%}$	3228	1220	2008
M^- versus IND	Root	72 hat	347	152	195
M^- versus IND	Shoot	72 hat	64	10	54
M^- versus IND	Root	240 hat	131	62	69
M^- versus IND	Shoot	240 hat	8	4	4

**Seed comparison M^+ versus bare M^-**			**Number DEGs based on p-value**		
			Total	UP in M^+	DOWN in M^+
M^+ versus M^-	Seed	$T_{1\%}$	180	62	118
M^+ versus M^-	Seed	$T_{100\%}$	55	32	23
M^+ versus M^-	Root	72 hat	3	3	0
M^+ versus M^-	Shoot	72 hat	2	2	0
M^+ versus M^-	Root	240 hat	11	10	1
M^+ versus M^-	Shoot	240 hat	12	12	0

Supplementary Table S2 | *Aethionema arabicum* gene names and IDs and *Arabidopsis thaliana* homologs for the presented expression results in Figures 8-14, and Supplemental Figures S5-S10. For further details about individual genes and gene IDs and link to the v3.1 genome (Fernandez-Pozo et al., 2021) see the Gene Expression Atlas (https://plantcode.cup.uni-freiburg.de/easy_gdb/tools/expression/expression_input.php).

Figure	Symbol	*Aethionema arabicum* gene ID (v2.5 genome)	*Arabidopsis thaliana* homolog (AGI)	TAIR (short description)
8A	AearADH1a	AA39G00353	AT1G77120	Alcohol dehydrogenase 1
8A	AearERF71/73	AA21G00481	AT1G72360	Integrase-type DNA-binding superfamily protein
8A	AearAC102	AA4G00115	AT5G63790	NAC domain containing protein 102
8A	AearPCO1	AA40G00576	AT5G15120	Protein of unknown function (DUF1637)
8A	AearPCO2	AA87G00177	AT5G39890	Protein of unknown function (DUF1637)
8A	AearACO1	AA33G00241	AT2G19590	1-Amino-cyclopropane-1-carboxylate (ACC) oxidase 1
8A	AearETR2	AA17G00111	AT3G23150	Signal transduction histidine kinase, hybrid-type, ethylene sensor
8B	AearCYP707A3a	AA6G00060	AT5G45340	Cytochrome P450, family 707, subfamily A, polypeptide 3
8B	AearNCED6	AA78G00012	AT3G24220	nine-cis-Epoxycarotenoid dioxygenase 6
8B	AearPYR1a	AA57G00057	AT4G17870	Polyketide cyclase/dehydrase and lipid transport superfamily protein
S5B	AearPYR1b	AA56G00008	AT4G17870	Polyketide cyclase/dehydrase and lipid transport superfamily protein
S5B	AearPYL2	AA32G01060	AT2G26040	PYR1-like 2
S5B	AearPYL3	AA31G00648	AT1G73000	PYR1-like 3
S5B	AearPYL4	AA805G00002	AT2G38310	PYR1-like 4
S5B	AearPYL6	AA29G00245	AT2G40330	PYR1-like 6
S5B	AearPYL7	AA32G00133	AT4G01026	PYR1-like 7
S5B	AearPYL11a	AA57G00145	AT5G45860	PYR1-like 11
S5B	AearPYL11b	AA6G00016	AT5G45860	PYR1-like 11
S5B	AearPYR/PYL	AA11G00152	AT5G53160	Regulatory components of ABA receptor 3
S5B	AearPYR/PYL	AA1G00061	AT1G01360	Regulatory component of ABA receptor 1
S5B	AearPYR/PYL	AA35G00054	AT5G05440	Polyketide cyclase/dehydrase and lipid transport superfamily protein
S5B	AearPYR/PYL	AA61G00520	AT1G01360	Regulatory component of ABA receptor 1
S5B	AearSDR1/ABA2	AA32G01008	AT1G52340	NAD(P)-binding Rossmann-fold superfamily protein
S5B	AearABI5	AA60G00170	AT2G36270	Basic-leucine zipper (bZIP) transcription factor family protein
S5B	AearAREB3a	AA93G00015	AT3G56850	ABA-responsive element binding protein 3

S5B	AearAREB3b	AA61G00149	AT3G56850	ABA-responsive element binding protein 3
S5B	AearABI5	AA60G00170	AT2G36270	Basic-leucine zipper (bZIP) transcription factor family protein
S5B	AearABF3	AA118G00053	AT4G34000	Abscisic acid responsive elements-binding factor 3
8B	AearGID1B	AA61G00628	AT3G63010	alpha/beta-Hydrolases superfamily protein (Gibberellin receptor GID1B)
8B	AearGASA7	AA123G00098	AT2G14900	Gibberellin-regulated family protein
S6A	AearGASA1	AA31G00380	AT1G75750	GAST1 protein homolog 1
S6A	AearGA3ox1	AA37G00176	AT1G15550	Gibberellin 3-oxidase 1
S6A	AearGA3ox2	AA31G00895	AT1G80340	Gibberellin 3-oxidase 2
S6A	AearGA3ox4	AA31G00896	AT1G80330	Gibberellin 3-oxidase 4
S6A	AearGID1A	AA10G00111	AT3G05120	alpha/beta-Hydrolases superfamily protein (Gibberellin receptor GID1A)
S6A	AearGAI	AA14G00090	AT1G14920	GRAS family transcription factor family protein (DELLA protein GAI)
S6A	AearRGA2	AA53G00639	AT2G01570	GRAS family transcription factor family protein (DELLA protein RGA2)
S6A	AearRGL2	AA10G00264	AT3G03450	RGA-like 2 (DELLA protein RGL2)
S5A	AearACS7	AA81G00052	AT4G26200	1-Amino-cyclopropane-1-carboxylate (ACC) synthase 7
S5A	AearACO2	AA53G01428	AT1G62380	1-Amino-cyclopropane-1-carboxylate (ACC) oxidase 2
S5A	AearACO4	AA19G00046	AT1G05010	1-Aminocyclopropane-1-carboxylate (ACC) oxidase 4 (ethylene-forming enzyme)
S5A	AearERF2	AA245G00010	AT5G47220	Ethylene responsive element binding factor 2
S5A	AearERF11	AA127G00014	AT1G28370	ERF domain protein 11
S5A	AearERF113/RAP2.6	AA44G00541	AT5G13330	Related to AP2 6l
S5A	AearRAP2.11	aa809g00001	AT5G19790	Related to AP2 11
9A	AearARF6	AA54G00428	AT1G30330	Auxin response factor 6
9A	AearARF11/19	AA39G00205	AT1G19220	Auxin response factor 19
9A	AearIAA2	AA17G00100	AT3G23030	Indole-3-acetic acid inducible 2
9A	AearIAA13	AA32G00458	AT2G33310	Auxin-induced protein 13
9A	AearIAA14	AA87G00261	AT4G14550	Indole-3-acetic acid inducible 14
9A	AearIAA17	AA19G00124	AT1G04250	AUX/IAA transcriptional regulator family protein
S6B	AearIAA4	AA32G00413	AT5G43700	AUX/IAA transcriptional regulator family protein
S6B	AearIAA10	AA19G00140	AT1G04100	Indoleacetic acid-induced protein 10
S6B	AearIAA18	AA15G00002	AT1G51950	Indole-3-acetic acid inducible 18
9B	Dormancy/auxin associated protein	AA184G00005	AT1G28330.5	Dormancy-associated protein-like 1
9B	AearSAUR11	AA8G00101	AT5G66260	SAUR-like auxin-responsive protein family
9B	AearSAUR20	AA10G00219	AT3G03820	SAUR-like auxin-responsive protein family

S6B	AearSAUR31	AA32G00176	AT4G00880	SAUR-like auxin-responsive protein family
S6B	AearSAUR51	AA31G00395	AT1G75580	SAUR-like auxin-responsive protein family
9B	AearSAUR72	AA26G00009	AT3G12830	SAUR-like auxin-responsive protein family
S6B	AearSAUR78	AA31G00698	AT1G72430	SAUR-like auxin-responsive protein family
9B	AearAUX1	AA385G00003	AT2G38120	Transmembrane amino acid transporter family protein
9B	AearABCG36	AA57G00466	AT1G59870	ABC-2 and Plant PDR ABC-type transporter family protein
10	AearPILS3	AA31G00310	AT1G76520	Auxin efflux carrier family protein
10	AearPILS7	AA8G00126	AT5G65980	Auxin efflux carrier family protein
10	AearPILS5	AA33G00056	AT2G17500	Auxin efflux carrier family protein
10	AearPILS6	AA32G01215	AT5G01990	Auxin efflux carrier family protein
11A	AearPIP3A/2;7	AA33G00001	AT4G35100	Plasma membrane intrinsic protein 3
11A	AearPIP1E/1;4	AA54G00312	AT4G00430	Plasma membrane intrinsic protein 1;4
11A	AearTIP1/1;1	AA60G00108	AT2G36830	gamma-Tonoplast intrinsic protein
11A	AearTIP4;1	AA32G01078	AT2G25810	Tonoplast intrinsic protein 4;1
11A	AearTIP2;3a	AA129G00005	AT5G47450	Tonoplast intrinsic protein 2;3
11A	AearTIP2;3b	AA129G00004	AT5G47450	Tonoplast intrinsic protein 2;3
11B	AearNTR2a	AA1187G00001	AT1G08090	Nitrate transporter 2:1
11B	AearNTR2b	AA233G00003	AT1G08090	Nitrate transporter 2:1
11B	AearNTR3.1	AA748G00001	AT5G50200	Nitrate transmembrane transporters
11B	AearNR1	AA31G00195	AT1G77760	Nitrate reductase 1
11B	AearNR2	AA54G00065	AT1G37130	Nitrate reductase 2
11B	AearNIR1	AA123G00071	AT2G15620	Nitrite reductase 1
S7A	AearCAB1	aa54g00401	AT1G29930	Chlorophyll A/B binding protein 1
S7A	AearLHCB2	aa53g01019	AT2G05100	Photosystem II light harvesting complex gene 2.1
S7A	AearKUP3	AA32G01007	AT2G26650	K+ transporter 1
S7A	AearABCG1	AA60G00279	AT2G39350	ABC-2 type transporter family protein
S7A	AearABCG2	aa60g00044	AT2G37360	ABC-2 type transporter family protein
S7A	AearABCG6	aa161g00007	AT5G13580	ABC-2 type transporter family protein
S7B	AearLAC5	aa29g00243	AT2G40370	Laccase 5
S7B	AearLTP3a	AA97G00013	AT5G59320	Lipid transfer protein 3
S7B	AearLTP3b	AA97G00014	AT5G59320	Lipid transfer protein 3
S7B	AearLTP3c	AA97G00016	AT5G59320	Lipid transfer protein 3
S7B	AearICL	AA26G00814	AT3G21720	Isocitrate lyase
S7B	AearOSM34	AA3G00018	AT4G11650	Osmotin 34
12	AearCHS/TT4	aa32g01209	AT5G13930	Chalcone and stilbene synthase family protein
12	AearCHI/TT5	aa70g00027	AT3G55120	Chalcone-flavanone isomerase family protein

12	AearCHI-like	aa35g00019	AT5G05270	Chalcone-flavanone isomerase family protein
12	AearFH3/TT6	aa13g00009	AT3G51240	Flavanone 3-hydroxylase
12	AearFLS1	aa44g00190	AT5G08640	Flavonol synthase 1
12	AearGSTF12/TT19	AA40G00386	AT5G17220	Glutathione S-transferase phi 12
12	AearTTG1	AA13G00129	AT5G24520	Transducin/WD40 repeat-like superfamily protein
13A	AearDOG1	AA6G00020	AT5G45830	Delay of germination 1
13A	AearSDR4L	AA39G00804	AT1G27461	Seed dormancy-4-like
13A	AearDLY1	AA184G00005	AT1G28330	Dormancy-associated protein-like 1
13A	AearNYE1/SGR1	AA57G00491	AT4G22920	Non-yellowing 1 (Protein STAY-GREEN 1, chloroplastic)
13B	AearNAC87	AA40G00281	AT5G18270	Arabidopsis NAC domain containing protein 87
13B	AearNAC3/59	AA53G01090	AT3G29035	NAC domain containing protein 3
13B	AearNAC2	AA1G00095	AT1G01720	NAC (No Apical Meristem) domain transcriptional regulator superfamily protein
13B	AearNAC32	AA31G00236	AT1G77450	NAC domain containing protein 32
S8B	AearNAC72	AA11G00017	AT4G27410	NAC (No Apical Meristem) domain transcriptional regulator superfamily protein
S8B	AearNAC81	AA44G00202	AT5G08790	NAC (No Apical Meristem) domain transcriptional regulator superfamily protein
13B	AearWRKY9	AA1278G00001	AT1G68150	WRKY DNA-binding protein 9
13B	AearWRKY56	AA53G01333	AT1G64000	WRKY DNA-binding protein 56
S8C	AearWRKY51	AA4G00214	AT5G64810	WRKY DNA-binding protein 51
S8C	AearWRKY4	AA14G00003	AT1G13960	WRKY DNA-binding protein 4
S8C	AearWRKY40	AA31G00850	AT1G80840	WRKY DNA-binding protein 40
S8A	AearMYB3	AA39G00452	AT1G22640	Myb domain protein 3
S8A	AearMYB6	AA9G00191	AT4G09460	Myb domain protein 6
S8A	AearMYB30	AA53G01079	AT3G28910	Myb domain protein 30
S8A	AearMYB66/WER	AA32G01148	AT5G14750	Myb domain protein 66
S8A	AearMYB49/39	AA11G00037	AT5G54230	Myb domain protein 49
S8A	AearMYB55/86	AA32G00209	AT4G01680	Myb domain protein 55
S8B	AearMYB101/GAMYB	AA32G00526	AT2G32460	Myb domain protein 101
S8B	AearMYB1R1	AA38G00086	AT1G70000	Myb-like transcription factor family protein
S8B	AearHB20	AA10G00418	AT3G01220	Homeobox protein 20
S8B	AearHB23	AA9G00043	AT5G39760	Homeobox protein 23
S8B	AearPLATZ1a	AA31G00305	AT1G21000	PLATZ transcription factor family protein
S8B	AearPLATZ1b	AA39G00329	AT1G21000	PLATZ transcription factor family protein
S8B	AearPLATZ3	AA53G00253	AT1G32700	PLATZ transcription factor family protein

S9A	AearGER3	AA8G00168	AT5G20630	Germin 3
S9A	AearLEA18/4-2	AA9G00144	AT2G35300	Late embryogenesis abundant protein, group 1 protein
S9A	AearLEA4-5	AA83G00024	AT5G06760	Late Embryogenesis Abundant 4-5
S9A	AearLEA14	AA1G00071	AT1G01470	Late embryogenesis abundant protein
S9A	AearLEA76/29	AA26G00277	AT3G15670	Late embryogenesis abundant protein (LEA) family protein
S9A	AearLEA (similar to PvLEA18)	AA3G00318	AT2G33690	Late embryogenesis abundant protein, group 6
S9B	AearLEA hydroxyproline-rich glycoproteine	AA15G00035	AT1G52330	Late embryogenesis abundant (LEA) hydroxyproline-rich glycoprotein family
S9B	AearNHL39 hydroxyproline-rich glycoproteine	AA592G00003	AT3G54200	Late embryogenesis abundant (LEA) hydroxyproline-rich glycoprotein family
S9B	AearHSP60	AA32G00466	AT2G33210	Heat shock protein 60-2
S9B	AearHSP70a	AA89G00021	AT1G79920	Heat shock protein 70 (Hsp 70) family protein
S9B	AearHSP70b	AA53G00294	AT4G16660	Heat shock protein 70 (Hsp 70) family protein
S9B	AearHSP81	AA10G00464	AT5G56000	Heat shock protein 81.4
14A	AearEXPA2	AA35G00022	AT5G05290	Expansin A2
14A	AearEXPA15	AA53G00740	AT2G03090	Expansin A15
14A	AearEXPB3	AA33G00348	AT4G28250	Expansin B3
S10A	AearEXPA4	AA29G00317	AT2G39700	Expansin A4
S10A	AearEXPA13	AA10G00293	AT3G03220	Expansin A13
S10A	AearEXPA18	AA53G01393	AT1G62980	Expansin A18
14B	AearbGAL4	AA44G00698	AT5G56870	beta-Galactosidase 4
14A	AearaXYL1	AA32G01046	AT1G68560	alpha-Xylosidase 1
14A	AearbXYL1	AA58G00011	AT5G49360	beta-Xylosidase 1
14A	AearbXYL2	AA7G00012	AT1G02640	beta-Xylosidase 2
14B	AearXTH2	AA69G00015	AT4G13090	Xyloglucan endotransglucosylase/hydrolase 2
14B	AearXTH32	AA60G00103	AT2G36870	Xyloglucan endotransglucosylase/hydrolase 32
14B	AearXTH12	AA44G00633	AT5G57530	Xyloglucan endotransglucosylase/hydrolase 12
14B	AearXTH23/XTR6	AA81G00019	AT4G25810	Xyloglucan endotransglycosylase 6
14B	AearXTH6	AA4G00292	AT5G65730	Xyloglucan endotransglucosylase/hydrolase 6
S10A	AearXTH24/MERI5B	AA102G00174	AT4G30270	Xyloglucan endotransglucosylase/hydrolase 24
S10A	AearXTH26	AA102G00042	AT4G28850	Xyloglucan endotransglucosylase/hydrolase 26
S10A	AearXTH31	AA72G00007	AT3G44990	Xyloglucan endo-transglycosylase-related 8
S10B	AearXTH30/XTR4	AA53G00205	AT1G32170	Xyloglucan endotransglucosylase/hydrolase 30
S10B	AearCSLC4	AA53G01041	AT3G28180	Cellulose-synthase-like C4
S10B	AearCSLD3	AA10G00310	AT3G03050	Cellulose synthase-like D3

S10B	AearAXY8a/FUC95Aa	AA118G00010	AT4G34260	1,2-alpha-L-Fucosidase
S10B	AearAXY8b/FUC95Ab	AA118G00011	AT4G34260	1,2-alpha-L-Fucosidase
S10B	AearAXY8c/FUC95Ac	AA118G00012	AT4G34260	1,2-alpha-L-Fucosidase
S10C	AearPLL22	AA109G00012	AT3G24670	Pectin lyase-like superfamily protein
S10C	AearPLL25	AA87G00376	AT4G13710	Pectin lyase-like superfamily protein
S10C	AearPGL3	AA38G00058	AT1G70370	Polygalacturonase 2
S10C	AearPGF6	AA74G00020	AT3G42950	Pectin lyase-like superfamily protein
S10D	AearPME5	AA271G00003	AT5G47500	Pectin lyase-like superfamily protein
S10D	AearPME41	AA93G00256	AT4G02330	Plant invertase/pectin methylesterase inhibitor superfamily
S10D	AearPMEI46	AA12G00202	AT5G04960	Plant invertase/pectin methylesterase inhibitor superfamily
S10D	AearPMEI/MPF21.4	AA151G00005	AT5G50030	Plant invertase/pectin methylesterase inhibitor superfamily protein

Supplemental References

Barrero, J.M., Millar, A.A., Griffiths, J., Czechowski, T., Scheible, W.R., Udvardi, M., Reid, J.B., Ross, J.J., Jacobsen, J.V., and Gubler, F. (2010). Gene expression profiling identifies two regulatory genes controlling dormancy and ABA sensitivity in *Arabidopsis* seeds. *Plant Journal* 61, 611-622.

Cao, J. (2012). The pectin lyases in *Arabidopsis thaliana*: Evolution, selection and expression profiles. *Plos One* 7, ARTN e46944.

Chandler, J.O., Wilhelmsson, P.K.I., Fernandez-Pozo, N., Graeber, K., Arshad, W., Pérez, M., Steinbrecher, T., Ullrich, K.K., Nguyen, T.-P., Mérai, Z., Mummenhoff, K., Theißen, G., Strnad, M., Mittelsten Scheid, O., Schranz, M.E., Petřík, I., Tarkowská, D., Novák, O., Rensing, S.A., and Leubner-Metzger, G. (2023). The dimorphic diaspore model *Aethionema arabicum* (Brassicaceae): Distinct molecular and morphological control of responses to parental and germination temperatures. *Plant Cell* revised manuscript under review, bioRxiv doi: https://doi.org/10.1101/2023.12.14.571707.

Choi, J., Kim, H., and Suh, M.C. (2023). Disruption of the ABA1 encoding zeaxanthin epoxidase caused defective suberin layers in Arabidopsis seed coats. *Frontiers in Plant Science* 14. https://doi.org/10.3389/fpls.2023.1156356

Daher, F.B., and Braybrook, S.A. (2015). How to let go: pectin and plant cell adhesion. *Frontiers in Plant Science* 6, ARTN 523.

De Los Reyes, B.G., Myers, S.J., and Mcgrath, J.M. (2003). Differential induction of glyoxylate cycle enzymes by stress as a marker for seedling vigor in sugar beet (*Beta vulgaris*). *Mol Genet Genomics* 269, 692-698.

Dong, T., Yin, X.M., Wang, H.T., Lu, P.Y., Liu, X., Gong, C.Y., and Wu, Y. (2021). ABA-INDUCED expression 1 is involved in ABA-inhibited primary root elongation via modulating ROS homeostasis in Arabidopsis. *Plant Science* 304, ARTN 110821.

Guo, L., Yang, H.B., Zhang, X.Y., and Yang, S.H. (2013). *Lipid transfer protein 3* as a target of MYB96 mediates freezing and drought stress in. *Journal of Experimental Botany* 64, 1755-1767.

Hundertmark, M., and Hincha, D.K. (2008). LEA (late embryogenesis abundant) proteins and their encoding genes in *Arabidopsis thaliana*. *BMC Genomics* 9, ARTN 118.

Liu, L.P., Zhao, L.H., Chen, P.J., Cai, H.Y., Hou, Z.M., Jin, X.Y., Aslam, M., Chai, M.N., Lai, L.Y., He, Q., Liu, Y.H., Huang, X.Y., Chen, H.H., Chen, Y.Z., and Qin, Y. (2020). ATP binding cassette transporters ABCG1 and ABCG16 affect reproductive development via auxin signalling in Arabidopsis. *Plant Journal* 102, 1172-1186.

Michel, B.E., and Kaufmann, M.R. (1973). Osmotic potential of polyethylene-glycol 6000. *Plant Physiology* 51, 914-916.

Moneo-Sanchez, M., Alonso-Chico, A., Knox, J.P., Dopico, B., Labrador, E., and Martin, I. (2019). beta-(1,4)-Galactan remodelling in Arabidopsis cell walls affects the xyloglucan structure during elongation. *Planta* 249, 351-362.

Pagnussat, L.A., Oyarburo, N., Cimmino, C., Pinedo, M.L., and De La Canal, L. (2015). On the role of a lipid-tansfer protein. Arabidopsis ltp3 mutant is compromised in germination and seedling growth. *Plant Signaling & Behavior* 10, ARTN e1105417.

Park, E.J., and Kim, T.H. (2021). Arabidopsis *OSMOTIN 34* functions in the ABA signaling pathway and is regulated by proteolysis. *International Journal of Molecular Sciences* 22, ARTN 7915.

Renard, J., Martinez-Almonacid, I., Castillo, I.Q., Sonntag, A., Hashim, A., Bissoli, G., Campos, L., Munoz-Bertomeu, J., Ninoles, R., Roach, T., Sanchez-Leon, S., Ozuna, C.V., Gadea, J., Lison, P., Kranner, I., Barro, F., Serrano, R., Molina, I., and Bueso, E. (2021). Apoplastic lipid barriers regulated by conserved homeobox transcription factors extend seed longevity in multiple plant species. *New Phytologist* 231, 679-694.

Scheler, C., Weitbrecht, K., Pearce, S.P., Hampstead, A., Buettner-Mainik, A., Lee, K., Voegele, A., Oracz, K., Dekkers, B., Wang, X., Wood, A., Bentsink, L., King, J., Knox, P., Holdsworth, M., Müller, K., and Leubner-Metzger, G. (2015). Promotion of testa rupture during garden cress germination involves seed compartment-specific expression and activity of pectin methylesterases. *Plant Physiology* 167, 200-215.

Silva, A.T., Ribone, P.A., Chan, R.L., Ligterink, W., and Hilhorst, H.W.M. (2016). A predictive coexpression network identifies novel genes controlling the seed-to-seedling phase transition in *Arabidopsis thaliana*. *Plant Physiology* 170, 2218-2231.

Smolikova, G., Strygina, K., Krylova, E., Vikhorev, A., Bilova, T., Frolov, A., Khlestkina, E., and Medvedev, S. (2022). Seed-to-seedling transition in *Pisum sativum* L.: a transcriptomic approach. *Plants (Basel)* 11, ARTN 1686.

Steinbrecher, T., and Leubner-Metzger, G. (2017). The biomechanics of seed germination. *Journal of Experimental Botany* 68, 765-783.

Steinbrecher, T., and Leubner-Metzger, G. (2022). Xyloglucan remodelling enzymes and the mechanics of plant seed and fruit biology. *Journal of Experimental Botany* 73, 1253-1257.

Van Der Weele, C.M., Spollen, W.G., Sharp, R.E., and Baskin, T.I. (2000). Growth of *Arabidopsis thaliana* seedlings under water deficit studied by control of water potential in nutrient-agar media. *Journal of Experimental Botany* 51, 1555-1562.

Verslues, P.E., and Bray, E.A. (2004). LWR1 and LWR2 are required for osmoregulation and osmotic adjustment in Arabidopsis. *Plant Physiology* 136, 2831-2842.

Wai, A.H., Rahman, M.M., Waseem, M., Cho, L.H., Naing, A.H., Jeon, J.S., Lee, D.J., Kim, C.K., and Chung, M.Y. (2022). Comprehensive genome-wide analysis and expression pattern profiling of PLATZ gene family members in *Solanum Lycopersicum* L. under multiple abiotic stresses. *Plants (Basel)* 11, ARTN 3112.

Wilhelmsson, P.K.I., Chandler, J.O., Fernandez-Pozo, N., Graeber, K., Ullrich, K.K., Arshad, W., Khan, S., Hofberger, J., Buchta, K., Edger, P.P., Pires, C., Schranz, M.E., Leubner-Metzger, G., and Rensing, S.A. (2019). Usability of reference-free transcriptome assemblies for detection of differential expression: a case study on *Aethionema arabicum* dimorphic seeds. *BMC Genomics* 20, ARTN 95.

Yadav, V., Molina, I., Ranathunge, K., Castillo, I.Q., Rothstein, S.J., and Reed, J.W. (2014). ABCG Transporters Are Required for Suberin and Pollen Wall Extracellular Barriers in. *Plant Cell* 26, 3569-3588.

Yonekura-Sakakibara, K., Yamamura, M., Matsuda, F., Ono, E., Nakabayashi, R., Sugawara, S., Mori, T., Tobimatsu, Y., Umezawa, T., and Saito, K. (2021). Seed-coat protective neolignans are produced by the dirigent protein AtDP1 and the laccase AtLAC5 in Arabidopsis. *Plant Cell* 33, 129-152.

Zinsmeister, J., Leprince, O., and Buitink, J. (2020). Molecular and environmental factors regulating seed longevity. *Biochemical Journal* 477, 305-323.
